# Multigene phylogenetics of *Sargassum* (Phaeophyceae) revealed low molecular diversity in contrast to high morphological variability in the NE Atlantic Ocean

**DOI:** 10.1111/jpy.13517

**Published:** 2024-10-26

**Authors:** Daniel Álvarez‐Canali, Marta Sansón, Carlos Sangil, Ana Tronholm

**Affiliations:** ^1^ Departamento de Botánica, Ecología y Fisiología Vegetal Universidad de La Laguna La Laguna Spain; ^2^ Department of Biological and Environmental Sciences University of Gothenburg Göteborg Sweden; ^3^ Gothenburg Global Biodiversity Centre Göteborg Sweden

**Keywords:** Fucales, molecular diversity, morphology, multigene phylogenetics, Phaeophyceae, *Sargassum*

## Abstract

*Sargassum* species play a key role in habitat formation in tropical and subtropical regions; however, species identification has been hampered by the phenological plasticity exhibited in response to environmental conditions and life history. Molecular phylogenetics has challenged taxa circumscriptions and proven critical in delimiting species in this genus. Yet, the Atlantic species of *Sargassum* remain poorly understood, and recent studies have shown low molecular diversity between the species in the NW Atlantic. Here, we expand the taxon sampling to the NE Atlantic to assess the diversity of *Sargassum* in the Atlantic basin, based on a comprehensive morphological and multigene approach. We selected genes commonly used in delineating species in this genus (ITS2, *rbc*LS, *cox*3, *mtsp*) and explored additional markers (*cox*2, *nad*6, *psb*C, *clp*C, *atp*B) to infer the phylogenetic relationships between the morphospecies observed in the NE Atlantic. Phylogenetic analyses using single‐gene and multigene alignments including 185 new sequences confirmed the low molecular diversity and supported the distinction of a single clade in *Sargassum* section *Sargassum* of N Atlantic benthic species. In contrast, morphological analysis resulted in the identification of 10 species and three new morphospecies that we described here but opt not to equate to species until further molecular evidence is available. Our results were congruent with previous findings from the NW Atlantic and highlight the morphological and ecological diversity of *Sargassum* in the Atlantic. These results suggest a recent colonization and incipient speciation of *Sargassum* in the Atlantic basin and showcase the need of further high‐throughput analyses.

**Abbreviations**
*atp*B: ATP synthase CF1 beta subunit
BI: Bayesian inference
*clp*C: Clp protease ATP‐binding subunit
*cox*2: cytochrome *C* oxidase subunit 2
*cox*3: cytochrome *C* oxidase subunit 3
ITS2: internal transcribed spacer 2
ML: maximum likelihood
*mtsp*
: 23S rRNA‐trnK intergenic spacer
mya: million years ago
*nad*6: NADH dehydrogenase subunit 6
*psb*C: photosystem II CP43 apoprotein
RAD: restricted‐site associated DNA
*rbc*LS: *rbc*L‐*rbc*S intergenic spacer

## INTRODUCTION

*Sargassum* is one of the richest genera of brown algae, with 356 accepted species to date (Guiry & Guiry, 2024; Stiger‐Pouvreau et al., 2023) and accounting for almost one‐sixth of the species in the class Phaeophyceae. *Sargassum* species are distributed worldwide and are especially well represented in tropical and subtropical regions, where they can form dense submarine forests and where they play key roles as habitat‐forming species, providing ecological services equivalent to the temperate *Fucus* and *Cystoseira* forests (Cheung‐Wong et al., 2022; Gouvêa et al., 2020; Hinojosa‐Arango et al., 2014; Stiger‐Pouvreau et al., 2023; Thiriet et al., 2016). Holopelagic *Sargassum* species can form prominent blooms along the tropical and subtropical N Atlantic, which have occurred almost yearly since 2010 and have spanned from W Africa to the Caribbean Sea (Fidai et al., 2020), becoming a nuisance in several countries as the biomass accumulates in the coasts, causing ecological, economic, and public health issues (Devault et al., 2021).

The classification of *Sargassum* species has been challenging since early phycological studies, mainly due to a high morphological variability of key characters (i.e., holdfast, main axes, phylloids, air bladders, and receptacles) at individual and population levels due to a distinct seasonal growth and in response to environmental conditions (Abbott et al., 1988; Endo et al., 2013; Kilar et al., 1992; Kim et al., 2022). From the early classification of five subgenera by Agardh (1889), and later modifications by Grunow (1915, 1916a, 1916b) and Yoshida (1983), *Sargassum* has been subjected to numerous nomenclatural changes. Moreover, during the last 3 decades, molecular data have revolutionized our understanding of the phylogenetic relationships in this genus, resulting in the resurrection of the two genera, *Sargassopsis* and *Phyllotricha*, and a broad reorganization of subgenera and sections, including the synonymizing of many species and a general trend of reducing the number of accepted species (Andrade‐Sorcia et al., 2014; Dixon et al., 2012; Draisma et al., 2010; Mattio et al., 2008, 2009, 2015). Currently, the genus *Sargassum* is comprised of two monophyletic subgenera: subgenus *Bactrophycus*, with sections *Halochloa*, *Hizikia*, *Spongocarpus*, and *Teretia* and subgenus *Sargassum*, including sections *Binderiana*, *Ilicifolia*, *Polycystae*, *Zygocarpicae*, *Sargassum* and a clade including several species grouped in sections not clearly resolved (including sections *Cladophyllum*, *Horridum*, *Herporhizum*, and *Lapazeanum* and the species *S. platycarpum* and *S. xochitliae*; Andrade‐Sorcia et al., 2014; Camacho et al., 2015; Dixon et al., 2014; González‐Nieto et al., 2020; Mattio & Payri, 2011; Yip et al., 2020). The use of molecular markers, such as the internal transcripted spacer 2 (ITS2) rRNA region and *rbc*LS and *cox*3 genes, has proven crucial for understanding the phylogenetic relationships of *Sargassum* species in the Pacific and Indian oceans (Cho et al., 2012; Huang et al., 2017; Mattio et al., 2013; Stiger et al., 2003), whereas these markers have not been as valuable for species delimitation in the Atlantic *Sargassum* (i.e., subgenus *Sargassum*, section *Sargassum*). Recent molecular studies based on the ITS2 rRNA region, *rbc*LS gene, and *cox*3 gene have attempted to evaluate the diversity of this genus in the W Atlantic with inconclusive results, as species showed little to no genetic diversity. Camacho et al. (2015) observed that all morphospecies within section *Sargassum* from Caribbean Colombia and adjacent areas (including seven recognized species and the newly described *S. giganteum*, as well as *S. scabridum* from New Zealand and *S. vulgare* from the Mediterranean) formed a polytomous clade, and they considered the morphological differences enough evidence for delineating species‐level boundaries. Later, González‐Nieto et al. (2020) obtained similar results for the Gulf of Mexico (including nine species and one variety), but they proposed the lumping of all sampled species into *Sargassum* cf. *cymosum*, despite morphological, ecological, and distributional differences. Complete mitogenomes and partial plastomes of the Atlantic holopelagic species *S. fluitans* and *S. natans* VIII revealed few genetic differences. The mitochondrial genomes only accounted for 93 nucleotide differences, distributed along 41 genes (ranging from one to 14 differences depending on the gene), and polymorphic sites in 19 chloroplastic genes, with the highest nucleotide changes located in the *cox*2, *nad*6, *psb*C, *clp*C, and *atp*B genes (Amaral‐Zettler et al., 2017). In the same study, the use of *cox*2 and *cox*3 genes was enough to distinguish between Atlantic holopelagic species, a distinction which has been confirmed in recent studies (Dibner et al., 2022; Siuda et al., 2024), in which authors were able to delimit the morphotypes of holopelagic *Sargassum* in the N Atlantic.

More than 30 species of *Sargassum* are currently recognized in the North Atlantic Ocean (Aouissi et al., 2018; John et al., 2004; Wynne, 2017), and within this basin, the Canary Islands stand out as a species diversity hotspot, in which the environmental conditions allow the coexistence of species from tropical and warm‐temperate affinities (Francisco‐Ortega et al., 2009). A vast number of species of *Sargassum* have been reported for this archipelago, and nine are currently accepted (Gallardo et al., 2016), including the morphologically distinct *S. desfontainesii*, which is mostly distributed in the NE Atlantic; the exclusively Atlantic‐Mediterranean *S. flavifolium*; the endemic *S. orotavicum*; and the widely distributed *S. acinarium*, *S. cymosum*, *S. filipendula*, *S. furcatum*, *S. natans*, and *S. vulgare*. Yet, only three species (*S. desfontainesii*, *S. orotavicum*, and *S. flavifolium*) have been morphologically assessed in the NE Atlantic (Díaz‐Villa et al., 2004, 2007; Sangil et al., 2015). Here, we conducted an analysis of the *Sargassum* morphospecies in the Canary Islands using a combined morphological and multigene approach using traditional markers for delineating species in this genus (the ITS2 rRNA region and the *rbc*LS, *cox*3, and *mtsp* genes) and additional genes (*cox*2, *nad*6, *psb*C, *clp*C, *atp*B), in order to: (1) assess the morphological and genetic diversity of *Sargassum* in the NE Atlantic; (2) infer the phylogenetic relationships of the species within the Atlantic basin; and (3) evaluate biogeographical species distribution.

## MATERIALS AND METHODS

### Field collections

Over 900 specimens of *Sargassum* were collected along the coasts of the Canary Islands over the span of 3 years (2019–2022), from the intertidal to 30 m depth, by scuba diving or snorkeling. Subsamples of apical fragments (young phylloids free of epiphytes) were preserved in silica gel for molecular analysis. All collected material was pressed as herbarium voucher specimens, and some fragments were preserved in 4% formalin seawater for further morphological studies. A total of 616 *exsiccata* were deposited in Herbarium TFC of the University of La Laguna, Spain (TFC Phyc 16449–16649; 16781–16785; 16790–16805; 16844–16854; 16865–16905; 16923–16926; 17000–17314; see Results for specific codes).

### Morphological analysis

Morphological examination was made on freshly collected specimens, herbarium‐dried material, and formalin‐preserved samples from the Canary Islands. To provide detailed descriptions of each morphospecies (sensu Lincoln et al., 1998), the most developed individuals of each species were selected, ranging from four to over 20 specimens per species, depending on material availability. For each selected specimen, habit, total length, and detailed measurements (length, width, and/or diameter) of holdfast, main axis (or stem), primary branches, phylloids (at least 10 per specimen), cryptostomata (at least 15 per specimen, from several phylloids), vesicles (up to 50 per specimen), and receptacles were measured. Gametangia were examined from transverse sections of receptacles that were made by hand with a razor blade and analyzed in a Leica DM50 microscope (Leica Microsystems, Germany). Specimens were assigned to species according to the morphological observations and identified to species level using specialize literature. Additionally, all *Sargassum* material collected prior to this study and deposited in Herbarium TFC, as well, a total of 179 *exsiccata* deposited in Herbarium BCM (University of Las Palmas de Gran Canaria, Spain), were analyzed to confirm records and for morphological comparison.

### DNA extraction and sequencing

Total genomic DNA was extracted from at least two individuals of each morphospecies (except the holopelagic species *Sargassum fluitans* and *S. natans* due to material availability) using DNeasy PowerPlant Pro Kit (Qiagen, Germany) according to manufacturer's instructions and subsequently purified with DNeasy PowerClean Pro Cleanup Kit (Qiagen, Germany). The nuclear ITS2 rRNA region, chloroplast‐encoded partial *rbc*L gene + spacer + partial *rbc*S (*rbc*LS) gene, and cytochrome c oxydase subunit 3 (*cox*3) gene were amplified following Camacho et al. (2015), Huang et al. (2017), Mattio et al. (2015), and Yip et al. (2018). An additional set of mitochondrial and chloroplastic genes were selected and amplified, as they have shown a certain level of diversity among closely related species of *Sargassum* (Amaral‐Zettler et al., 2017; Dibner et al., 2022; Mattio & Payri, 2010): cytochrome c oxydase subunit 2 (*cox*2), the cytochrome c oxydase subunit 3 proposed by Dibner et al. (2022; extended *cox*3), NADH dehydrogenase 6 (*nad*6), 23S rRNA‐trnK intergenic spacer (*mtsp*), ATP synthase CF1 beta subunit (*atp*B), clp protease ATP binding subunit (*clp*C), and photosystem II CP43 reaction center protein (*psb*C). All primers and polymerase chain reaction (PCR) conditions used in this study are listed in Tables 1 and 2, respectively. The PCR amplifications were performed on an S1000 Thermal Cycler (Bio‐Rad, UK) with a total volume of 25 μL containing 1 μL of genomic DNA, 12.5 μL of DreamTaq Green PCR Master Mix (2×; Thermo Scientific), 0.5 μL of each forward and reverse primers (10 μM), and molecular grade water. The PCR products were purified using QIAquick PCR Purification Kit (Qiagen, Germany) following manufacturer's recommendations and sequenced at Eurofins Genomics Europe.

**TABLE 1:** List of primers used in this study for the amplification of selected genes in *Sargassum*.

Gene	Primer	Sequence	References
*cox*3	*CAF4A*	ATGTTTACTTGGTGRAGRGA	Mattio et al. (2013)
	*CAR4A*	CCCCACCARTAWATNGTNAG	Mattio et al. (2013)
ITS‐2	*5.8S‐BF*	CGATGAAGAACGCAGCGAAATGCGAT	Mattio et al. (2013)
	*25BR‐2*	TCCTCCGCTTAGTATATGCTTAA	Mattio et al. (2013)
*rbc*LS	*3F*	CATCGTGCTGGTAACTCTAC	Mattio et al. (2013)
	*S97R*	CATCTGTCCATTCWACACTAAC	Mattio et al. (2013)
*cox*2	*cox2 507F*	GTAGATTATTTGACCCCTAG	This study
	*cox2 1098R*	CAATATAGTCCAACCAATCT	This study
	*cox2 948F*	GATCCTAGTTTGACGATAAA	This study
	*cox2 1634R*	TCTTAAAAATGGAACTGCTA	This study
	*cox2_1498F*	AGGGTGATTCCTCTATATTT	This study
	*cox2_2609R*	TTTTATTTTCGTGGAGGTAT	This study
	*cox2_2435F*	ACAATTAAAATCAGAGCATG	This study
	*cox2_3718R*	AGAATTTAAGAGCATAACCT	This study
*cox*3	*Sarg cox3 F*	GTTCGAATCCTATCCCCTTCTTAA	Dibner et al. (2022)
	*Sarg cox3 R*	GGCCAAACCCCTCCAATATTA	Dibner et al. (2022)
*mtsp*	*mtsp‐F*	GCTACATCGAGAAGAGATAA	This study
	*mtsp‐R*	GATACTATAACGGACCACTT	This study
*nad*6	*Sarg nad6 F* (*External*)	TATGATTCTTGGGGCTGGT	Dibner et al. (2022)
	*Sarg nad6 R* (*External*)	GGGATCATTCAAAGCAGAAGA	Dibner et al. (2022)
*atp*B	*atpB_105F*	TAATGCTCTCAAAGTAGAAG	This study
	*atpB_1400R*	TTGATATCACCAACCAAATA	This study
*clp*C	*clpC_97F*	ATACTCTTAGGTCTATTAGGA	This study
	*clpC_1295R*	CTTTATCAAGAATATAGGCAG	This study
*psb*C	*psbC_34F*	TGTTTGACGATTTATACCTT	This study
	*psbC_1301R*	TATCGGGTAAGTTATTAGGT	This study

**TABLE 2:** Polymerase chain reaction primer pairs and conditions for the amplification of selected genes in *Sargassum*.

Gene	Forward primer	Reverse primer	Initial denaturation	# cycles	Denaturation	Annealing	Extension	Final extension
*cox*3	*CAF4A*	*CAR4A*	94°C 1 min	40 cycles	94°C 40 s	42°C 30 s	72°C 45 s	72°C 7 min
ITS‐2	*5.8S‐BF*	*25BR‐2*	94°C 1 min	40 cycles	94°C 40 s	55°C 30 s	72°C 45 s	72°C 7 min
*rbc*LS	*3F*	*S97R*	94°C 1 min	40 cycles	94°C 40 s	44°C 30 s	72°C 45 s	72°C 7 min
*cox2*(a)	*cox2_507F*	*cox2_1098R*	95°C 2 min	40 cycles	95°C 40 s	45°C 30 s	72°C 45 s	72°C 5 min
*cox2*(b)	*cox2_948F*	*cox2_1634R*	95°C 2 min	40 cycles	95°C 40 s	45°C 30 s	72°C 45 s	72°C 5 min
*cox2*(c)	*cox2_1498F*	*cox2_2609R*	95°C 2 min	40 cycles	95°C 40 s	45°C 30 s	72°C 50 s	72°C 5 min
*cox2*(d)	*cox2_2435F*	*cox2_3718R*	95°C 2 min	40 cycles	95°C 40 s	45°C 30 s	72°C 50 s	72°C 5 min
*cox*3	*Sarg cox3 F*	*Sarg cox3 R*	94°C 4 min	5 cycles	94°C 60 s	50°C 30 s	68°C 60 s	68°C 7 min
				5 cycles	94°C 60 s	48°C 30 s	68°C 60 s	
				10 cycles	94°C 60 s	46°C 30 s	68°C 60 s	
				10 cycles	94°C 60 s	44°C 30 s	68°C 60 s	
				10 cycles	94°C 60 s	42°C 30 s	68°C 60 s	
*mtsp*	*mtsp_F*	*mtsp_R*	95°C 2 min	40 cycles	95°C 40 s	45°C 30 s	72°C 45 s	72°C 5 min
*nad*6	*Sarg nad6F* (*external*)	*Sarg nad6R* (*external*)	94°C 4 min	5 cycles	94°C 60 s	50°C 30 s	68°C 60 s	68°C 7 min
				5 cycles	94°C 60 s	48°C 30 s	68°C 60 s	
				10 cycles	94°C 60 s	46°C 30 s	68°C 60 s	
				10 cycles	94°C 60 s	44°C 30 s	68°C 60 s	
				10 cycles	94°C 60 s	42°C 30 s	68°C 60 s	
*atp*B	*atpB_105F*	*atpb_1400R*	95°C 2 min	40 cycles	95°C 40 s	45°C 30 s	72°C 45 s	72°C 5 min
*clp*C	*clpC_97F*	*clpC_1295R*	95°C 2 min	40 cycles	95°C 40 s	45°C 30 s	72°C 45 s	72°C 5 min
*psb*C	*psbC_34F*	*psbC_1301R*	95°C 2 min	40 cycles	95°C 40 s	45°C 30 s	72°C 45 s	72°C 5 min

### Taxon sampling and phylogenetic analyses

A dataset of newly generated sequences and additional sequences retrieved from GenBank was created for each gene (see Tables [Link], [Link] in the Supporting Information), including species of the subgenera *Sargassum* and *Bactrophycus*, as well as all sections within the subgenus *Sargassum*, in particular those generated from Atlantic samples. *Sargassopsis decurrens*, *Turbinaria gracilis*, and *T. ornata* were used as outgroups and, additionally, *Sargassum fulvelum*, depending on sequence availability. Sequences were aligned using Clustal Omega and individual gene alignments were revised manually. All subsequent analyses were performed via the CIPRES Science gateway web server (Miller et al., 2010) and the CIPRES REST API (Miller et al., 2015). For each gene, the best‐fitting nucleotide substitution model was selected using jModelTest 2.1.6 (Darriba et al., 2012) with Akaike information criterion (Akaike, 1973). Maximum likelihood (ML) analyses of single‐gene and concatenated *cox*3‐ITS2‐*rbc*LS alignments were performed using RAxML 8.2.12 set as follows: a rapid bootstrap analysis and search for the best‐scoring ML tree in one single run with 1000 bootstrap replicates under GTR + I + G model. Bayesian inference (BI) analyses were performed for both individual and concatenated alignments using MrBayes 3.2.7. Two independent runs of four Markov chain Monte Carlo iterations were run simultaneously for 5 × 10^6^ generations, sampling every 1000 generations and with a 10% burn‐in, under the GTR + G + I model.

## RESULTS

### Nuclear and cytoplasmic markers variability

A total of 74 new sequences—22 *cox*3, 26 ITS2, and 26 *rbc*LS—were obtained from 27 individuals collected in the Canary Islands (Table S1), representing 11 morphospecies. Single‐gene alignments were built and comprised a total of 80, 95, and 87 sequences and 500, 606, and 757 nt total lengths, respectively. The concatenated *cox*3‐ITS2‐*rbc*LS alignment consisted of a matrix of 97 sequences. For the additional genes, a total of 101 new sequences—35 *mtsp*, 8 *atp*B, 8 *clp*C, 12 *nad*6, 14 *psb*C, 12 *cox*2, and 12 extended *cox*3—were obtained from the most morphologically distinct morphospecies (Tables [Link], [Link]) and alignments comprised 82, 30, 30, 41, 37, 41, and 54 sequences, and 512, 1437, 1174, 804, 1425, 3275, and 819 nt lengths, respectively.

All newly generated sequences for the ITS2 rRNA region and the *rbc*LS, *atp*B, *clp*C, *psb*C, extended *cox*3, and *nad*6 genes were identical for each individually. In contrast, the *cox*2 gene sequence OR786537 had a duplication of six nucleotides at position 2167 and the *cox*2 gene sequence OR783540 had a substitution at 2499. Most *cox*3 gene sequences shared one ambiguous position (R) at position 142. The *mtsp* gene showed the highest variability: a substitution at position 82 (OR786562 and OR786566), a substitution at position 85 (OR786561), a substitution at position 255 (OR786569 and OR786573), a substitution at position 241 (OR786577), and a six‐nucleotide mutation (GATTAA/TTATAC) between positions 137 and 142. The frequency of this mutation varied between sequences: 18 shared haplotype GATTAA and 16 TTATAC, and one showed both haplotypes simultaneously. A closer scrutiny of the mitochondrial genes' alignments used in the phylogenetic analysis revealed a set of unique mutations shared in all Canarian sequences and not present in either the Atlantic *Sargassum fluitans* or *S. natans*: four mutations in *cox*2 (three substitutions at positions 2373, 2606, and 2713 and a deletion of 12 nucleotides at positions 1240–1251) and three in *nad*6 (substitutions at positions 351, 401, and 408). For the *mtsp* gene, we observed a mutation at position 298 that was unique to the Canarian samples, and the six‐nucleotide mutation (at position 177–183 in the alignment) was shared with either *S. fluitans* (GATTAA) or *S. natans* (TTAATC). In addition, three ambiguous positions in the *mts*p gene were also present in all Canarian samples (R at 268, W at 386, and R at 348) and was shared with some of the sequences retrieved from GenBank, the first with HQ416095, HQ416108, and HQ416118 and the second with FM993055 and FM993074. All new sequences for each chloroplastic gene were identical to either *S. fluitans*, *S. natans*, or both.

### Single‐gene and concatenated phylogenies

**FIGURE 1:**
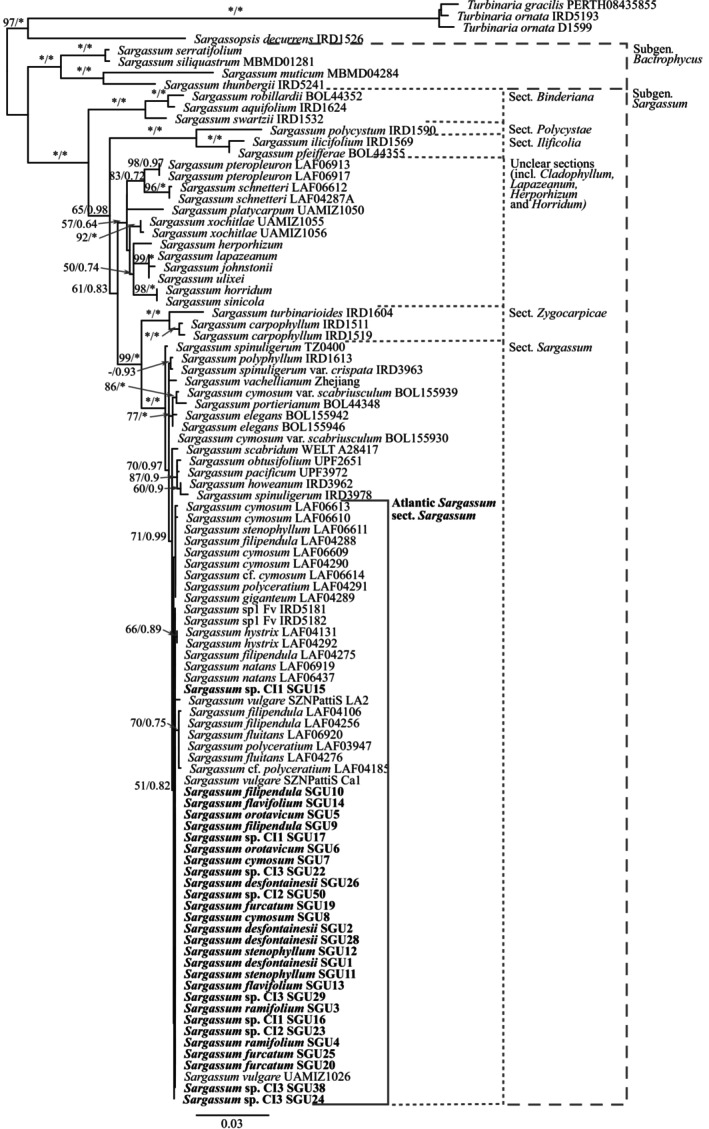
Maximum likelihood phylogenetic tree of the concatenated dataset (*cox*3 + ITS2 + *rbc*LS) showing both subgenus and accepted sections within subgenus *Sargassum*. Values at the nodes indicate bootstrap support (left) and posterior probability (right). Values below 60/0.6 are not shown. Asterisk (*) indicates full support. Sequences generated in this study in bold.

Maximum likelihood and BI single‐gene and concatenated phylogenies of *cox*3 gene, ITS2 rRNA region, and *rbc*LS gene recovered both subgenera *Bactrophycus* and *Sargassum*, as well as the sections within the subgenus. *Sargassum* with high support (Figure 1; Figures [Link], [Link] in the Supporting Information). In contrast, the putative sections *Cladophyllum*, *Horridum*, *Herporhizum*, *Lapazeanum* and the species *S. platycarpum* and *S. xochitliae* were resolved in a polytomous clade with low support, except for with the *cox*3 gene, as only two species from section *Cladophyllum* were included. Section *Binderiana* was the first diverging clade within the subgenus. *Sargassum*; section *Polycystae* and *Ilicifolia* were resolved as sister clades; and section *Zygocarpicae* was recovered as sister of section *Sargassum* in the *cox*3 and *rbc*LS gene phylogenies and concatenated phylogeny. The ITS2 rRNA region phylogeny (Figure S2) only resolved with high support the relationship among *Zygocarpicae*‐*Sargassum* and *Polycystae‐Ilicifolia*, while both clades, along with all other sequences of the unresolved sections, fell within the same low‐resolution polytomous branch.

All newly generated sequences representing 11 morphospecies clustered within section *Sargassum* in a polytomous branch (Figure 1), but some groups of sequences clustered with low support (e.g., the pair *S. hyxtrix* LAF04131 and LAF04292 and the group consisting of *S. filipendula* LAF04106 and LAF04256, *S. fluitans* LAF06920 and LAF04276, and *S. polyceratium* LAF03947); however, these clusters were not consistent among different genes (Figures [Link], [Link], [Link]). Most of the Canarian sequences grouped in polytomous clades that included specimens morphologically assigned to different morphospecies. All Atlantic sequences fell within a single unsupported polytomy in the *cox*3 gene phylogeny and concatenated analysis.

**FIGURE 2:**
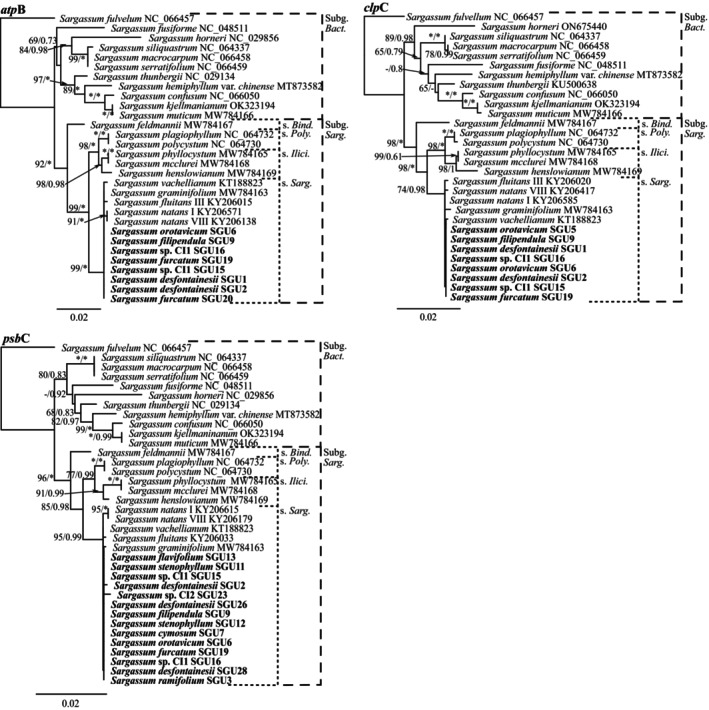
Maximum likelihood phylogenetic tree of *Sargassum* for the chloroplastic protein‐coding genes *atp*B, *clp*C, and *psb*C, showing both subgenus and accepted sections within subgenus *Sargassum* (abbreviated). Values at the nodes indicate bootstrap support (left) and posterior probability (right). Values below 60/0.6 are not shown. Asterisk (*) indicates full support. Sequences generated in this study in bold.

**FIGURE 3:**
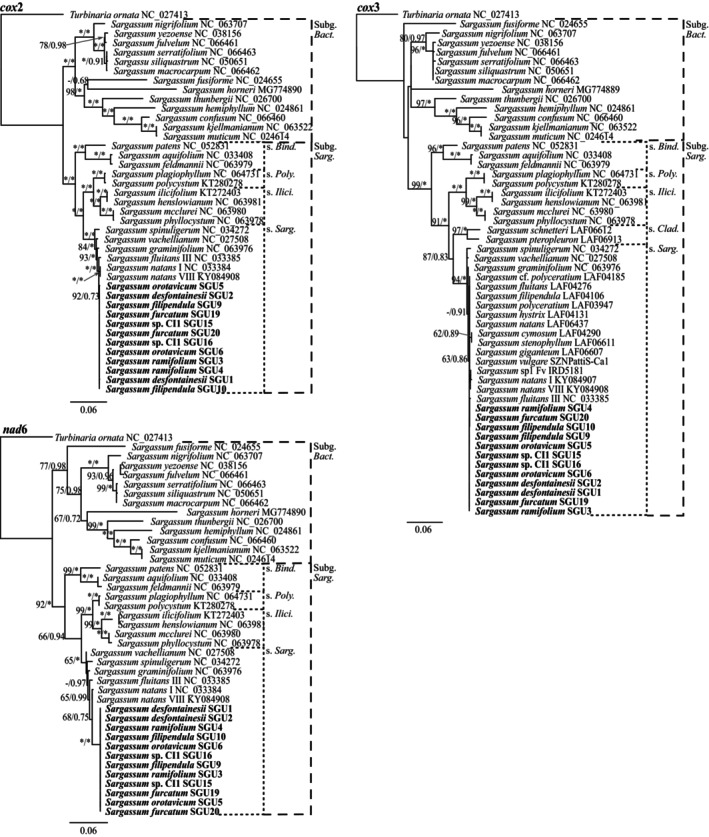
Maximum likelihood phylogenetic tree of *Sargassum* for the mitochondrial protein‐coding genes *cox*2, extended *cox*3, and *nad*6, showing both subgenus and accepted sections within subgenus *Sargassum* (abbreviated). Values at the nodes indicate bootstrap support (left) and posterior probability (right). Values below 60/0.6 are not shown. Asterisk (*) indicates full support. Sequences generated in this study in bold.

**FIGURE 4:**
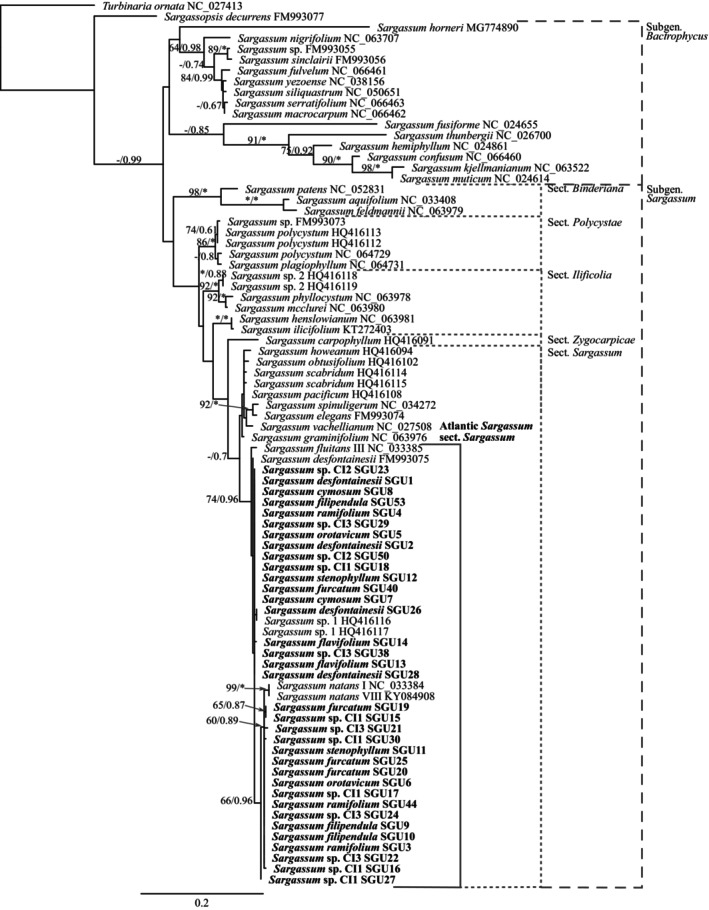
Maximum likelihood phylogenetic tree of *Sargassum* based in *mtsp* sequences. Values at the nodes indicate bootstrap support (left) and posterior probability (right). Values below 60/0.6 are not shown. Asterisk (*) indicates full support. Sequences generated in this study in bold.

For the additional protein‐coding genes phylogenies (i.e., *cox*2, extended *cox*3, *nad*6, *atp*B, *clp*C, and *psb*C), both subgenera and all sections were recovered with very high support in the ML and BI analyses (Figures 2 and 3). Topologies were almost identical for the six genes, except for *Sargassum horneri*, *S. fusiforme*, and *S. fulvelum* (subgenus. *Bactrophycus*), as they had very divergent sequences. Likewise, the Canarian sequences clustered within section *Sargassum*, including all morphospecies and the Atlantic species *S. fluitans* and *S. natans*, forming a polytomous clade with high support (Figures 2 and 3). The analysis of the intergenic spacer *mtsp* resulted in highly polytomous phylogenies with very low support at all levels for both BI and ML analyses (Figure 4).

### Morphology and taxonomy

A thorough examination of *Sargassum* specimens from the Canary Islands (see details in Materials and Methods) led to the identification of 13 distinct morphospecies, 10 of which were consistent with previously described species in the Atlantic, and all displayed the morphological traits of *Sargassum* subgenus *Sargassum*. Of these, six were regarded as currently present in the Canary Islands (*S. cymosum*, *S. desfontainesii*, *S. filipendula*, *S. flavifolium*, *S. furcatum*, and *S. orotavicum*). Two had been previously recorded but had been regarded as doubtful for the region or included as synonyms under other names (*S. natans* and *S. ramifolium*), and two constituted new records (*S. fluitans* and *S. stenophyllum*). The remaining three could not be assigned to any described species; however, we took the conservative approach and regarded them as *Sargassum* sp. 1, *Sargassum* sp. 2, and *Sargassum* sp. 3 until further molecular evidence is available. Taxonomic implications and morphological descriptions of *Sargassum* from the Canary Islands are detailed for each morphospecies separately below.

### *Sargassum cymosum* C. Agardh, 1820 (Figure 5a–c)

**FIGURE 5:**
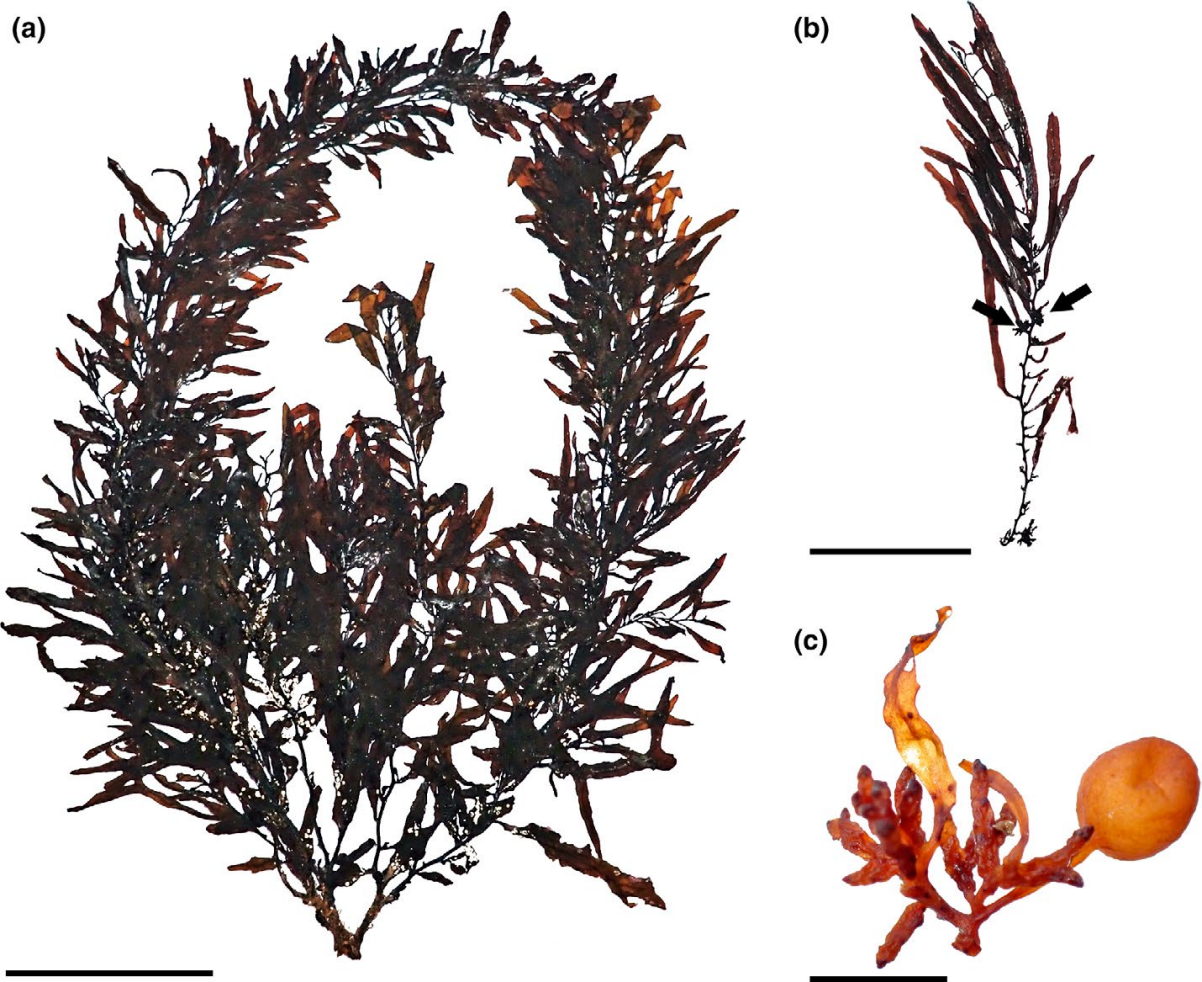
*Sargassum cymosum* TFC Phyc 16530. (a) Habit of a mature individual. Scale bar = 5 cm. (b) Detail of a branch with linear‐lanceolate phylloids with entire margins, bearing receptacles (arrows). Scale bar = 3 cm. (c) Detail of an axillary vesicle and receptacle. Scale bar = 3 mm. [Color figure can be viewed at wileyonlinelibrary.com]

**Type specimen.** LD 2979 (Lund University: Lund, Sweden; Camacho et al., 2015)

**Type locality.** Brazil (*In mari Atlantico*, *ad littora Brasiliae*; C. Agardh, 1820, p. 20)

**Representative material.** TFC Phyc 16455–56, TFC Phyc 16530–31, 16531dupl

**Morphology.** Thalli up to 20 cm high, dark brown. Holdfast discoid‐conical up to 30 mm in diameter, bearing one or rarely two main axes, up to 28 mm high and 4.4 mm in diameter, simple, usually with scars of fallen branches. Primary branches are coriaceous, smooth throughout, terete, up to 190 mm long and 2.1 mm in diameter, sometimes producing secondary branches. Phylloids crowded, oval to linear‐lanceolate, simple, rarely once forked, up to 40 mm long and 3.6–5(–5.6) mm wide tapering toward the base and apices, margin entire, frequently undulated, obtuse tips, percurrent midrib, and a short smooth stalk 1–2 mm long. Vesicles are scarce, spherical, in the axils of phylloids, 3.5–4.5 mm in diameter, smooth, with a terete stalk, 2–4 mm long, and 0.2 mm in diameter. Cryptostomata are frequent, scattered on phylloid surfaces, elliptical 26–99 μm long, and 17–63 μm wide. Thalli monoecious, receptacles arranged in dense racemose groups in the axils of phylloids on secondary branches, often two to three times irregularly branched, rarely simple, terete with wart‐like surface, up to 6.1 mm long and 0.9 mm wide, with a very short or rarely 2.3 times branched sterile stalk.

**Distribution.** Widely distributed in tropical and subtropical waters of the Atlantic Ocean. Records from outside the Atlantic are dubious (Camacho et al., 2015)

**Habitat.** Infrequent species, observed in tidal pools and on rocky platforms of the lower intertidal at wave‐exposed localities, mixed with *Gongolaria* and *Cystoseira* species. In the field, phylloids feel thicker and stiffer than the other species observed in the Canary Islands.

**Remarks.** This species was first reported in the archipelago in 1972 (Afonso‐Carrillo et al., 1983), but the revision of the original vouchers and others deposited under this name in TFC or BCM revealed that they do not correspond with the modern concept of *Sargassum cymosum*, as they have more slender, mostly forked, and several times furcate, linear phylloids that fit better with the concepts of *S. ramifolium* and *S. stenophyllum*. A note by M.C. Gil‐Rodríguez on voucher TFC Phyc 4210 (originally identified as *S. cymosum*, later revised as *S. ramifolium*) helps to explain this confusion, as it confirms that the identification was based on Schnetter (1976), who regarded individuals with simple and furcate phylloids as *S. cymosum*.

### *Sargassum desfontainesii* (Turner) C. Agardh, 1820 (Figure 6a–c)

**FIGURE 6:**
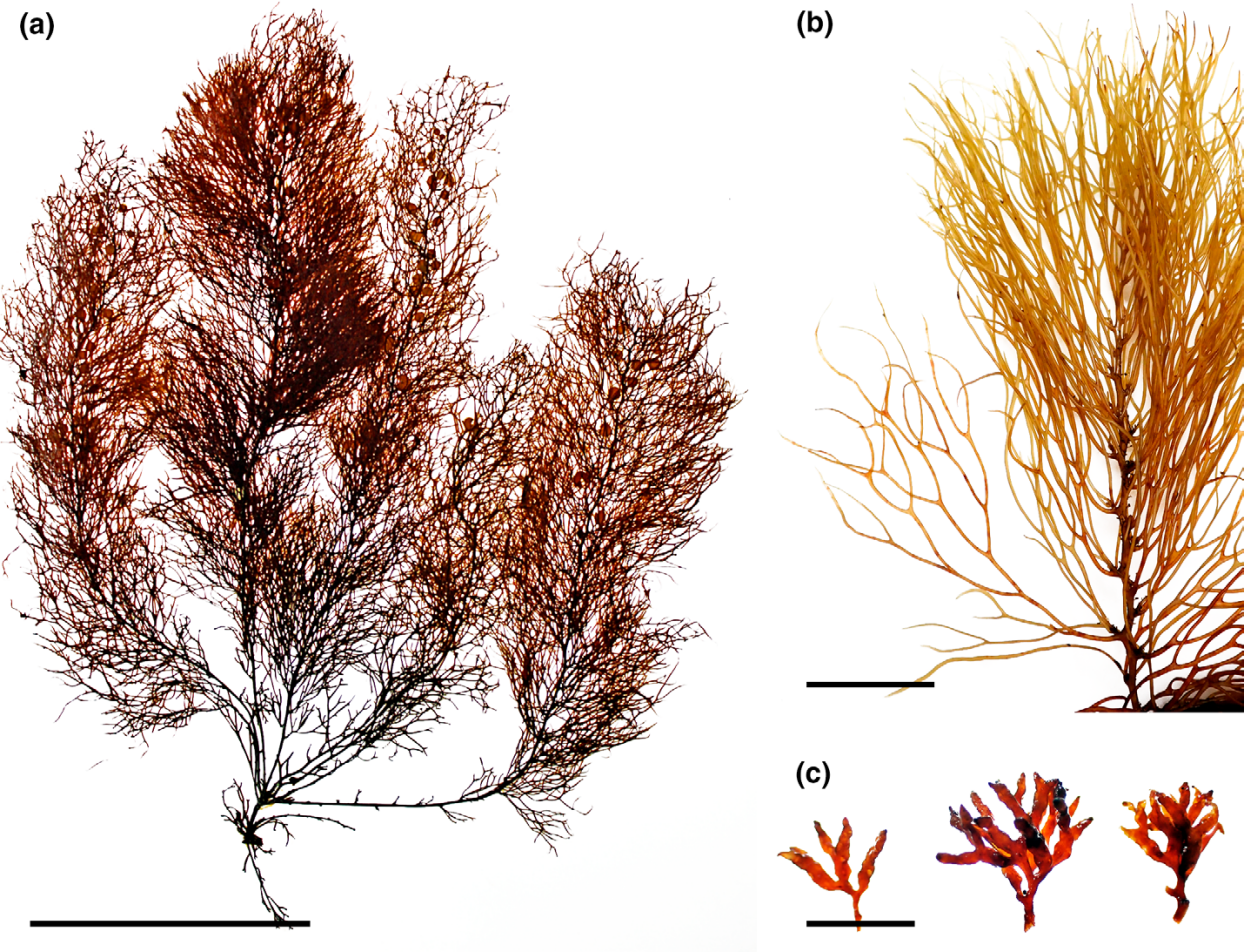
*Sargassum desfontainesii* TFC Phyc 16506. (a) Habit of a mature individual with abundant vesicles. Scale bar = 10 cm. (b) Detail of a branch with cylindrical, several times bifurcated phylloids. Scale bar = 2.5 cm. (c) Detail of densely branched, racemose receptacles. Scale bar = 4 mm. [Color figure can be viewed at wileyonlinelibrary.com]

**Type specimen.** BM 000562938 or BM 000519479 (Natural History Museum: London, UK)

**Type locality.** Canary Islands (*On the shores of the Canary Islands*; Turner, 1811, p. 130)

**Synonymy.** *Fucus desfontainesii* Turner, 1811, p. 130, pl. 190

**Representative material.** TFC Phyc 13110, TFC Phyc 15827–36, TFC Phyc 16505–08, TFC Phyc 16537–40, TFC Phyc 16557–59

**Morphology.** Thalli up to 50 cm high, yellowish brown. Holdfast discoid to irregular, up to 38 mm in diameter, sometimes confluent in dense populations, bearing up to 15 main axes, up to 51 mm high and 7 mm in diameter, simple to several times branched, usually with scars of fallen branches. Primary branches are coriaceous, smooth, terete to slightly compressed, up to 480 mm long and 2 mm in diameter, producing secondary and rarely tertiary branches. Phylloids filiform, up to eight times regularly forked with wide branching angles, 86 mm long and 2 mm wide, slightly flattened at each bifurcation point, lacking midrib, sessile, and ending in acute apices. Vesicles in the axils of phylloids, spherical to slightly oblong, 2–6 mm in diameter, some with one or two apical spine‐like mucro or rarely a short terminal phylloid; stalk 2–5(–6) mm long and 1 mm in diameter, rarely branched and bearing two vesicles. Cryptostomata are scarce to absent, irregularly scattered on the phylloid surfaces, elliptical, with a central pore 60–214 μm long and 24–143 μm wide. Thalli androdioecious. Receptacles arranged solitary or in short branchlets in the axils of phylloids or vesicles on secondary or tertiary branches with up to 12 receptacles in alternate‐spiral sequence. Receptacles terete with somewhat wart‐like surface, (2–) 4 (–7) times branched in cymose clusters with a short stalk; androgynous receptacles verrucose, (3–) 4 (–7) mm long and (0.5–) 1.0 (–1.5) mm wide decreasing in diameter toward the apices, bearing male and female conceptacles; male receptacles less verrucose, slightly longer, and slender, (4–)5(–6) mm long and (0.5–) 0.7(–1.2) mm wide.

**Distribution.** Northeastern Atlantic: Canary Islands, Madeira, Azores. Mediterranean: Melilla (González‐García & Conde, 1992)

**Habitat.** Common in rocky intertidal, growing in mid‐ to low‐intertidal pools and subtidal to 6 m depth. It can form very dense populations on the edges of pools, with densities of over 100 individuals · m^−2^. More frequent in wave‐exposed localities.

**Remarks.** This species is readily identified by its filiform, highly ramified phylloids without midrib, which is a unique character in the genus. This species has been reported for the Caribbean, but Taylor (1960) considered the early records from Guadalupe uncertain, and examination of collections from Trinidad (MICH 610261; Richardson, 1975) and Venezuela (WNC‐A‐002388; Ganesan, 1990) depicted flat phylloids with a marked midrib, which do not match the morphology of this species but rather resemble the widely distributed *Sargassum ramifolium*. Similarly, some of the material from the Canary Islands examined in this study and originally identified as *S. desfontainesii* corresponded to *S. ramifolium*. Other records outside the Lusitanian province (Spalding et al., 2007) or adjacent regions should be taken with caution, as they are probably misidentifications.

### *Sargassum filipendula* C. Agardh, 1824 (Figure 7a–c)

**FIGURE 7:**
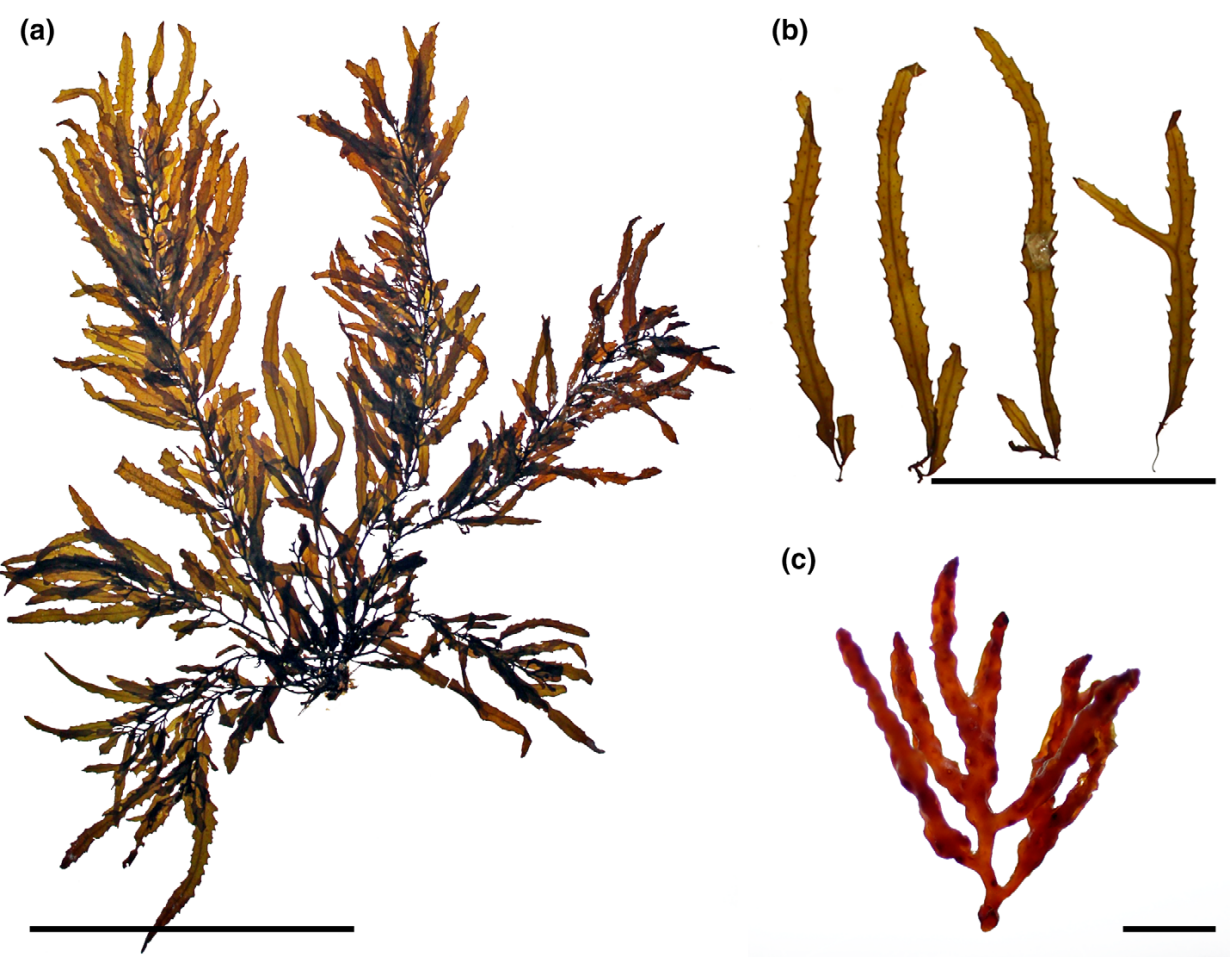
*Sargassum filipendula*. (a) Habit of a mature individual (TFC Phyc 16524). Scale bar = 10 cm. (b) Detail of the long, serrated phylloids with a short stalk (TFC Phyc 16457). Scale bar = 5 cm. (c) Detail of a loose racemose receptacle. Scale bar = 2 mm. [Color figure can be viewed at wileyonlinelibrary.com]

**Type specimen.** LD 3253 (Lund University: Lund, Sweden); lectotype designated from the syntype collection by Hanisak and Kilar (1990)

**Type locality.** West Indies “India Occidentalis, Aspegren” (Hanisak & Kilar, 1990; *In sinu mexicano?*; C. Agardh, 1824, p. 300)

**Synonymy.** *Sargassum affine* J. Agardh, 1848, p. 343. *Sargassum filipendula* f. *subcirerea* Grunow, 1916b, p. 171

**Representative material.** TFC Phyc 10, TFC Phyc 6833, 6833dupl., TFC Phyc 16457–58, TFC Phyc 16524–27

**Morphology.** Thalli up to 60 cm high, yellowish brown. Holdfast discoid‐conical up to 20 mm in diameter, bearing from one to three main axes, up to 10 mm high and 3 mm in diameter, with scars of fallen branches. Primary branches are coriaceous, smooth, terete to slightly compressed, up to 590 mm long and 1.2 mm in diameter, producing secondary and tertiary branches. Phylloids linear‐lanceolate, simple, very rarely forked near the tip, up to 86 mm long and 8 mm wide, margins markedly serrate, with slightly asymmetrical base, acute tips, and percurrent midrib. Vesicles in the axils of phylloids, spherical to slightly oblong, 4–8 mm in diameter, some with an apical spine‐like mucro; stalk terete to slightly flattened, smooth, 3–6 mm long. Cryptostomata are numerous and scattered over the phylloid surfaces, round to elliptical, 109–209 μm long, and 90–171 μm wide. Thalli monoecious (androgynous). Receptacles are solitary or forming loose racemose or cymose groups in the axils of phylloids. Receptacles terete, somewhat warty, up to 8.9 mm long and 0.9 mm wide, simple to two to three times branched, born on a single to two to three times ramified sterile stalk.

**Distribution.** Widely distributed in tropical and subtropical waters of both sides of the Atlantic Ocean. Reported from the Northeastern Atlantic: Canary Islands, Salvage Islands, and Madeira. Also documented from some localities in the Middle East, Southwest, and Southeast Asia (Guiry & Guiry, 2024), although records outside the Atlantic Ocean should be taken with caution.

**Habitat.** Infrequent species, growing with other *Sargassum* in low‐intertidal pools in semi‐exposed to exposed localities. The largest individuals are observed in subtidal habitats as usual with other *Sargassum* species in the archipelago.

**Remarks.** The type specimen has very narrow phylloids, less than 2 mm wide; however, later descriptions of this species included individuals having phylloids up to 8(–15) mm wide (Camacho et al., 2015; Schneider & Searles, 1991; Taylor, 1960). Furthermore, the majority of identifications seem to omit phylloid width when assigning specimens to this species, and the two most used characters to identify this species are the simple, long, and regularly serrated phylloids and the presence of large vesicles with long stalks, usually longer than the diameter of the vesicle.

### *Sargassum flavifolium* Kützing, 1849 (Figure 8a–c)

**FIGURE 8:**
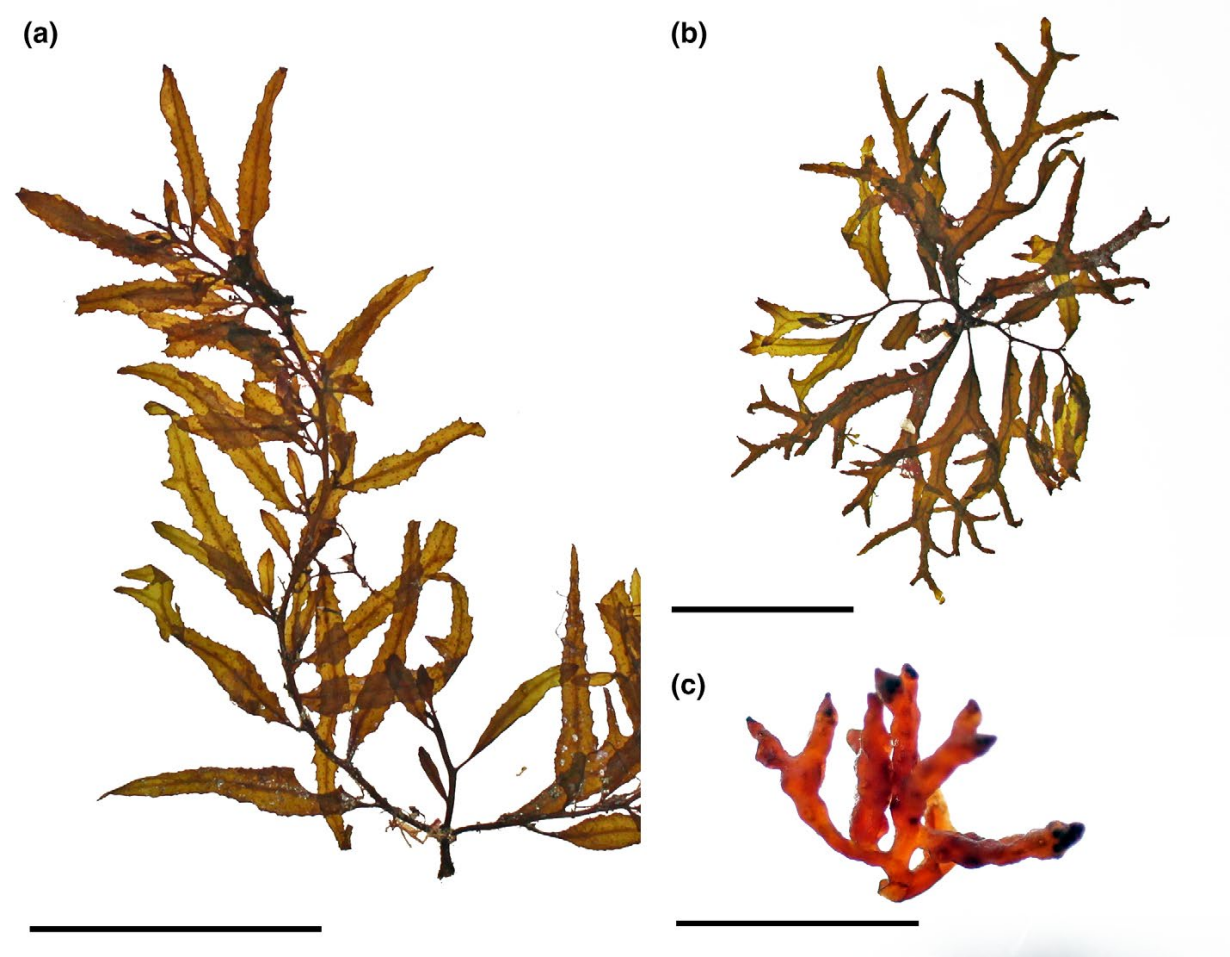
*Sargassum flavifolium*. (a) Habit of an immature individual with mostly entire to furcate phylloids (TFC Phyc 16495). Scale bar = 5 cm. (b) Detail of a young individual with a very short stalked and several times pinnate‐furcate phylloids (TFC Phyc 16492). Scale bar = 5 cm. (c) Detail of the short, thick receptacles; scale bar = 4 mm. [Color figure can be viewed at wileyonlinelibrary.com]

**Type specimen.** Unknown.

**Type locality.** Biarritz, France (*Ad* Antillas; *in sinu* Biscayense *ad* Biaritz *legit* Fleischer; Kützing, 1849, p. 615)

**Synonymy.** *Sargassum vulgare* var. *flavifolium* (Kützing) Grunow, 1916a, p. 40.

**Representative material.** TFC Phyc 2651, TFC Phyc 16461–62, TFC Phyc 16492–95, TFC Phyc 16535

**Morphology.** Thalli up to 65 cm high, yellowish brown. Holdfast discoid‐conical up to 30 mm in diameter, bearing up to 11 main axes, up to 27 mm high and 3.6 mm in diameter, simple, with scars of fallen branches. Primary branches are coriaceous, smooth, terete, up to 635 mm long and 1.3 mm in diameter, producing secondary branches. Phylloids linear‐lanceolate, from simple to up to six to seven times pinnate‐furcate, up to 50 mm long and 3.5–5(–6.1) mm wide, margins serrate, acute tips, percurrent midrib, and a short stalk 1–2 mm long. Vesicles in the axils of phylloids, spherical to slightly oblong, 3.5–6.8 mm in diameter, rarely with an apical spine‐like mucro; stalk terete, 2–5 mm long, and 0.2 mm in diameter. Cryptostomata are frequent and scattered over the phylloid surfaces, elliptical, 56–149 μm long, and 36–148 μm wide. Thalli monoecious, receptacles are arranged solitary in the axils of phylloids or vesicles on the secondary branches. Receptacles are terete, up to five times irregularly dichotomously branched, with wart‐like surface, up to 6.8 mm long and 1 mm wide, stalk extremely short to absent.

**Distribution.** NE Atlantic, from France to Morocco, including the Canary Islands and the Mediterranean (Aouissi et al., 2018)

**Habitat.** This species is observed almost exclusively in the subtidal. Common from 10 to 20 m depth, although it can also be observed in shallower waters, growing on rocks covered with a thin layer of sand, usually in wave‐sheltered habitats.

**Remarks.** This species reaches the farthest north in the NE Atlantic, excluding the invasive *Sargassum muticum* (Gruet, 1984). Its presence in the western Atlantic seems to be an error, as indicated by the original diagnosis, and it has not been recorded in the area since (Aouissi et al., 2018; Littler & Littler, 2000; Taylor, 1960; Wynne, 2017).

### *Sargassum fluitans* (Børgesen) Børgesen, 1914a (Figure 9a–c)

**FIGURE 9:**
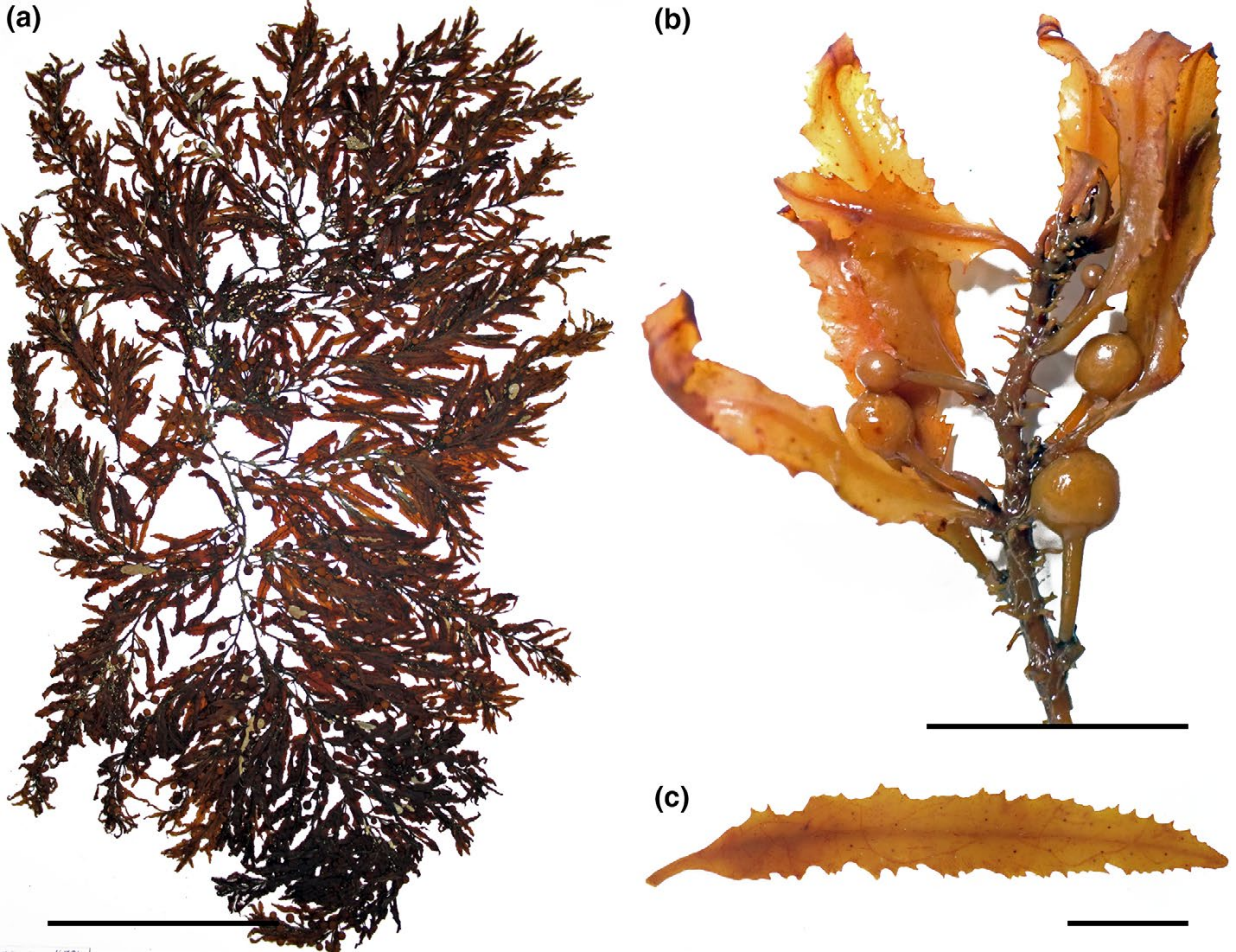
*Sargassum fluitans*. (a) Habit of a developed individual (TFC Phyc 16784). Scale bar = 10 cm. (b) Detail of an apex, showing the abundant spines in the branches and long pedicelate spherical air bladders. Scale bar = 2 cm. (c) Detail of a phylloid, with minute yet frequent cryptostomata. Scale bar = 1 cm. [Color figure can be viewed at wileyonlinelibrary.com]

**Type specimen.** C‐A‐92173 (University of Copenhagen: Copenhagen, Denmark)—Lectotype (Camacho et al., 2015)

**Type locality.** North Atlantic “Sargasso Sea” “which I have collected in the Sargasso‐Sea”; Børgesen (1914b).

**Synonymy.** *Sargassum hystrix* var. *fluitans* Børgesen.

**Representative material.** TFC Phyc 16790–95, TFC Phyc 16797–805.

**Morphology.** Thallus yellowish‐brown, pelagic, without a holdfast or main axis. Primary branches are coriaceous, with abundant spines especially near the apices, terete to slightly compressed, more than 500 mm long and 2.2 mm in diameter, frequently producing secondary branches. Phylloids linear‐lanceolate, simple, up to 52 mm long and 9.1 mm wide, margins markedly serrate, with symmetrical base, somewhat rounded tips, and percurrent midrib. Vesicles either in the axils of phylloids or forming directly from the branches, spherical to slightly oblong, 4–6.6 mm in diameter, smooth or rarely with a lateral mucro‐like projection, a small phylloid or a vesicle; stalk terete to slightly flattened, rarely alate, smooth, up to 6.3 mm long, and 1.4 mm diameter. Cryptostomata from abundant to absent in most phylloids, randomly scattered on the surface, round, up to 180 μm diameter. Receptacles not observed

**Distribution.** Widely distributed in the warm temperate to tropical Atlantic Ocean. Together with *Sargassum natans*, it is one of the species historically present in the Sargasso Sea and both form the Great Atlantic Sargassum Belt (Wang et al., 2019).

**Habitat.** Pelagic

**Remarks.** This study represents the first record of *Sargassum fluitans* in the Canary Islands. Morphology is in accordance with most descriptions from the western Atlantic (e.g., Børgesen, 1914a, 1914b; Parr, 1939; Taylor, 1960), except for the presence of cryptostomata, which some authors have not recorded (Ballantine et al., 2021; Camacho et al., 2015; Chapman, 1963; Kergosien et al., 2024; Rosado‐Espinosa et al., 2020; Siuda et al., 2024). All *S. fluitans* specimens collected in the Canary Islands have come from an unprecedented stranding event that begun in March 2024. During March–May 2024, rafts of pelagic *Sargassum*, including both *S. fluitans* and *S. natans*, were observed throughout the archipelago, and many ended up stranded along the coast of the islands.

### *Sargassum furcatum* Kützing, 1843 (Figure 10a–c)

**FIGURE 10:**
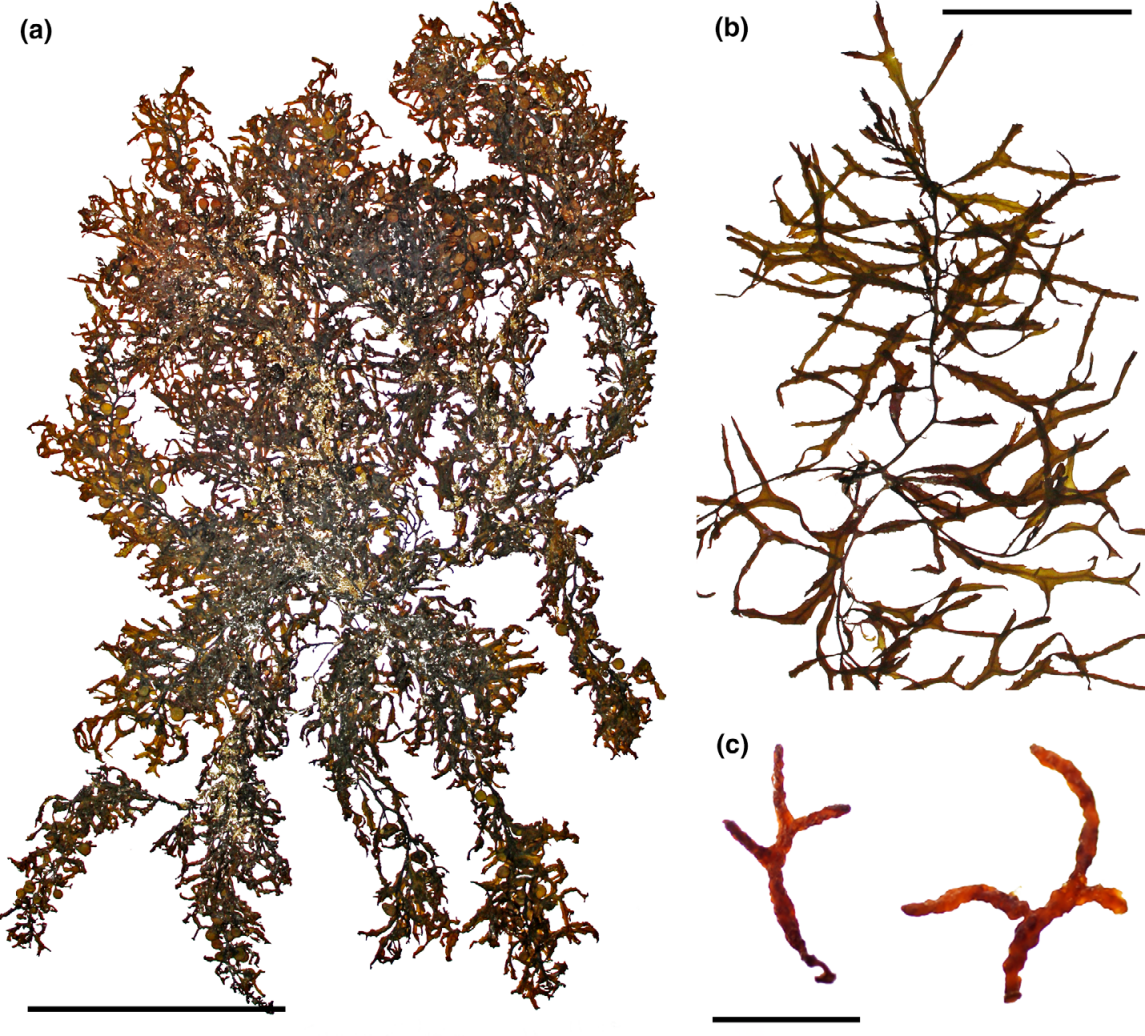
*Sargassum furcatum*. (a) Habit of a mature, well‐developed individual with several main branches, crowded phylloids and abundant vesicles (TFC Phyc 16495). Scale bar = 10 cm. (b) Detail of an individual with sparser, furcate phylloids (TFC Phyc 16492). Scale bar = 5 cm. (c) Detail of irregularly dichotomous ramified receptacles. Scale bar = 5 mm. [Color figure can be viewed at wileyonlinelibrary.com]

**Type specimen.** L 005639 (Naturalis Biodiversity Center: Leiden, The Netherlands)

**Type locality.** St. Thomas (*St. Thomas: Ehrenberg!*; Kützing, 1843, p. 362)

**Synonymy.** *Sargassum vulgare* f. *furcatum* (Kützing) J. Agardh, 1889

**Representative material.** TFC Phyc 475, TFC Phyc 3368, TFC Phyc 4871, TFC Phyc 6405, TFC Phyc 16467–68, TFC Phyc 16532–34

**Morphology.** Thalli up to 35 cm high, dark brown. Holdfast discoid‐conical up to 35 mm in diameter, bearing up to three main axes, up to 13 mm high and 3.45 mm in diameter, simple, with scars of fallen branches. Primary branches are coriaceous, smooth in the proximal third, and bearing spines in the distal portion, terete, up to 305 mm long and 1.20 mm in diameter, producing secondary branches. Phylloids linear‐lanceolate, rarely simple, usually five to six times furcate, up to 53 mm long and 1.8–3.9(–5.5) mm wide tapering toward the base and apices, margins serrate, acute tips, percurrent midrib, and a short stalk 1–2 mm long. Vesicles in the axil of phylloids, spherical, 3.5–5.5 mm in diameter, rarely with an apical spine‐like mucro; stalk terete, 2–5 mm long and 0.2 mm in diameter. Cryptostomata frequent, scattered on the phylloid surfaces, round to elliptical, 108–232 μm long, and 78–170 μm wide.

Thalli monoecious, receptacles are solitary or arranged in loose racemose clusters in the axils of phylloids or vesicles of the secondary branches. Receptacles are terete, once or twice irregularly dichotomously branched, with wart‐like surfaces, up to 10 mm long and. 0.9 mm wide, with a very short sterile stalk.

**Distribution.** Widely distributed in tropical and subtropical waters of the North Atlantic, including the Mediterranean (Guiry & Guiry, 2024)

**Habitat.** Rarely observed. This species grows in tide pools in sympatry with other *Sargassum* species, and in the shallow subtidal, usually less than 5 m depth, in semi‐exposed localities.

### *Sargassum natans* (Linnaeus) Gaillon, 1828 (Figure 11a,b)

**FIGURE 11:**
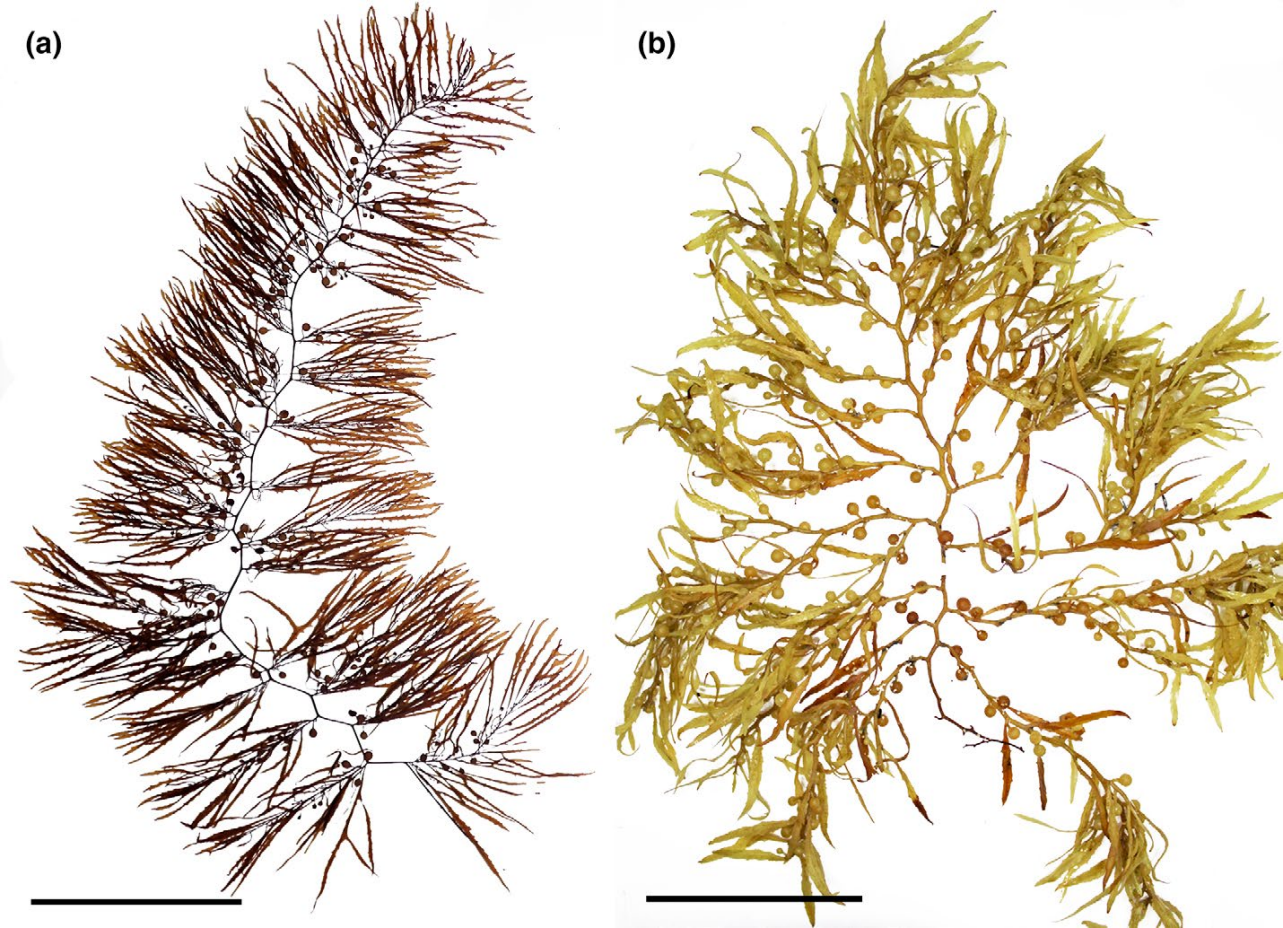
*Sargassum natans*. (a) Habit of morphotype I, with characteristic mucronate air bladders (TFC Phyc 15838). (b) Habit of morphotype VIII, with a generally more robust aspect and smooth air bladders (TFC Phyc 16885). Scale bars = 10 cm. [Color figure can be viewed at wileyonlinelibrary.com]

**Type specimen.** LINN 1274.35 (Linnean Society: London, UK); lectotype designated by Børgesen (1914b)

**Type locality.** North Atlantic “Sargasso sea” (Børgesen, 1914b; *in Pelago libere natans*, Linnaeus, 1753, p. 1160). Lectotype locality: “Indica” (probably Jamaica) (Børgesen, 1914b; Silva et al., 1996)

**Synonymy.** *Fucus natans* Linnaeus, 1753. *Baccalaria natans* (Linnaeus) S.F. Gray, 1821

**Representative material.** TFC Phyc 15838, TFC Phyc 16854, TFC Phyc 16865–94.

**Morphology.** Thallus yellowish‐brown, pelagic, without a holdfast or main axis. Primary branches are coriaceous, smooth, terete to slightly compressed, up to 472 mm long and 3 mm in diameter, producing secondary branches. Phylloids linear, simple, rarely forked once or twice, up to 80 mm long and 7.9 mm wide, margins markedly serrate, with slightly asymmetrical base, acute to rounded tips, and percurrent midrib. Vesicles either in the axils of phylloids or forming directly from the branches, spherical to slightly oblong, 2–8.5 mm in diameter, in some individuals often with an apical spine‐like mucro or a terminal phylloid, in others smooth; stalk terete to slightly flattened, rarely alate, smooth, 2.8–7.7 mm long. Cryptostomata are absent, but some rounded dark spots can appear on the phylloids. Receptacles not observed.

**Distribution.** Widely distributed in the warm temperate to tropical Atlantic Ocean. *Sargassum natans* has been recorded in the past in the Atlantic coasts of Europe as well as Azores, Madeira, Canary Islands, and Cape Verde archipelagos (Guiry, 2001; Guiry & Guiry, 2024).

**Habitat.** Pelagic

**Remarks.** The arrival of *Sargassum natans* to the Canary Islands coasts has seemed to occur quite sporadically based on the lack of any previous material, and until this work, no specimen was deposited in either TFC or BCM that could confirm its presence in the Canary Islands. Even though it has been repeatedly documented in the area (Agardh, 1848; Bory de Saint‐Vincent, 1828; de Toni, 1895; de Viera y Clavijo, 1869; Piccone, 1884), these early records were considered dubious as early naturalists frequently referred this species to any floating *Sargassum* (Gil‐Rodríguez et al., 1986; Martín Aguado, 1957). According to the commonly reported morphotypes for this species (Machado et al., 2022; Parr, 1939), the single individual collected in 2005 (TFC Phyc 15838) corresponds to *S. natan*s I, and in the extensive collections from the ongoing stranding event starting in March 2024, both *S. natans* I and VIII were collected.

### *Sargassum orotavicum* Díaz‐Villa, Afonso‐Carrillo, & Sansón 2004 (Figure 12a–c)

**FIGURE 12:**
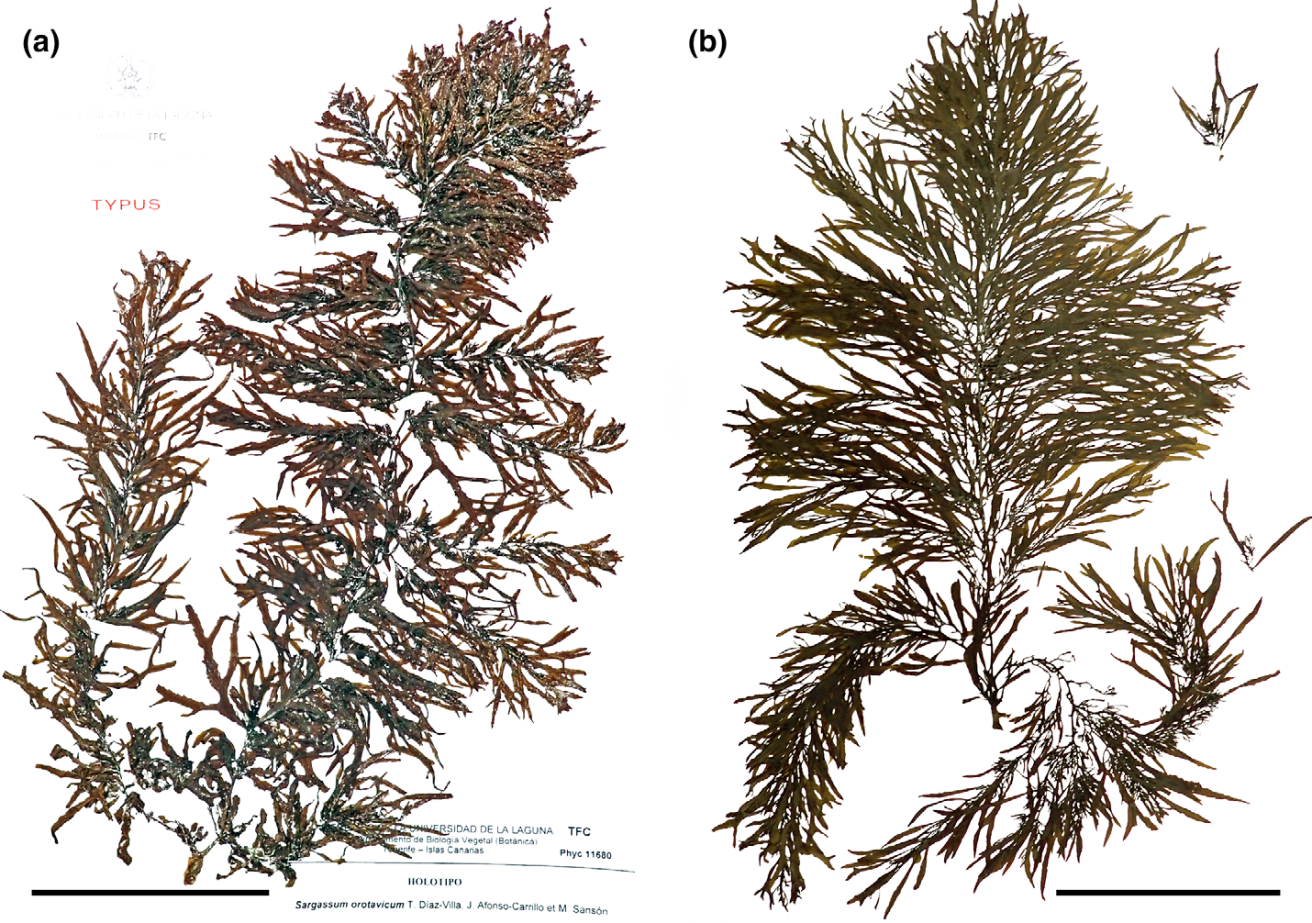
*Sargassum orotavicum*. (a) HOLOTYPE (TFC Phyc 11680). (b) TFC Phyc 16564. Scale bars = 10 cm. [Color figure can be viewed at wileyonlinelibrary.com]

**Type specimen.** TFC Phyc 11680 (Universidad de La Laguna: Tenerife, Spain)

**Type locality.** Puerto de la Cruz, Tenerife, Canary Islands: wave‐exposed tide pool (Díaz‐Villa et al., 2004, p. 479)

**Representative material.** TFC Phyc 11675–85, TFC Phyc 15838, TFC Phyc 16453–54, TFC Phyc 16521–23

**Morphology.** Thalli up to 62 cm high, greenish brown. Holdfast discoid‐conical up to 25 mm in diameter, sometimes confluent in dense populations, bearing a single or rarely up to six main axes, up to 42 mm high and 8 mm in diameter, with scars of fallen branches. Primary branches are coriaceous, smooth on the proximal portions but densely covered with spines at the distal ends, terete to slightly compressed, up to 462 mm long and 3 mm in diameter, forming secondary branches. Phylloids dimorphic between primary and secondary branches. Primary phylloids lanceolate to linear‐lanceolate, sometimes forked three times, up to 65 mm long and 11 mm wide, narrower in the proximal portions of the thallus, margins serrate to nearly entire, with asymmetrical base, acute tips, and percurrent midrib; secondary phylloids simple, linear‐lanceolate, up to 50 mm long and 8 mm wide, margins slightly dentate‐serrate to entire, with asymmetrical bases; stalks short, slightly flattened, 1–2 mm long, and often with two opposite spines parallel to the phylloid plane. Vesicles are spherical to slightly oblong, 2–9 mm in diameter, some with an apical spine‐like mucro or rarely a short terminal phylloid; stalk terete to slightly flattened, smooth, or occasionally spinous, 3–6 mm long. Cryptostomata are numerous and scattered over the phylloid surfaces and occasionally on vesicles, round to elliptical, 24–190 μm long, and 14–148 μm wide. Thalli monoecious, receptacles are arranged solitary or more often in short branchlets in the axils of phylloids or vesicles of the secondary branches, up to 25 mm long and bearing up to nine receptacles in alternate‐spiral sequence. Receptacles terete, somewhat warty, and rarely furnished with acute spines, up to 17 mm long and 2 mm in diameter, one to seven times branched in dense clusters, branches one to two times forked, with a very short stalk.

**Distribution.** Currently only known from the Canary Islands

**Habitat.** In rocky mid‐ to low‐intertidal eulittoral pools of wave‐exposed localities, and sometimes in the shallow sublittoral to 4 m depth. It can form very dense populations in low intertidal pools, with densities over 100 individuals · m^−2^. Only a handful of populations of this species are known, on the north coast of Tenerife and possibly in Gran Canaria.

**Remarks.** The species was described from a tide pool in Puerto de la Cruz (north of Tenerife) by Díaz‐Villa et al. (2004) that no longer hosts any *Sargassum*; however, during this study, a new population was observed a few kilometers from the type locality with individuals that fit the original diagnosis. Young specimens are difficult to distinguish from other *Sargassum*, but when fertile, the morphological features of receptacles and general morphology make it easily distinguishable from the other species present in the Canaries.

### *Sargassum ramifolium* Kützing, 1843 (Figure 13a–c)

**FIGURE 13:**
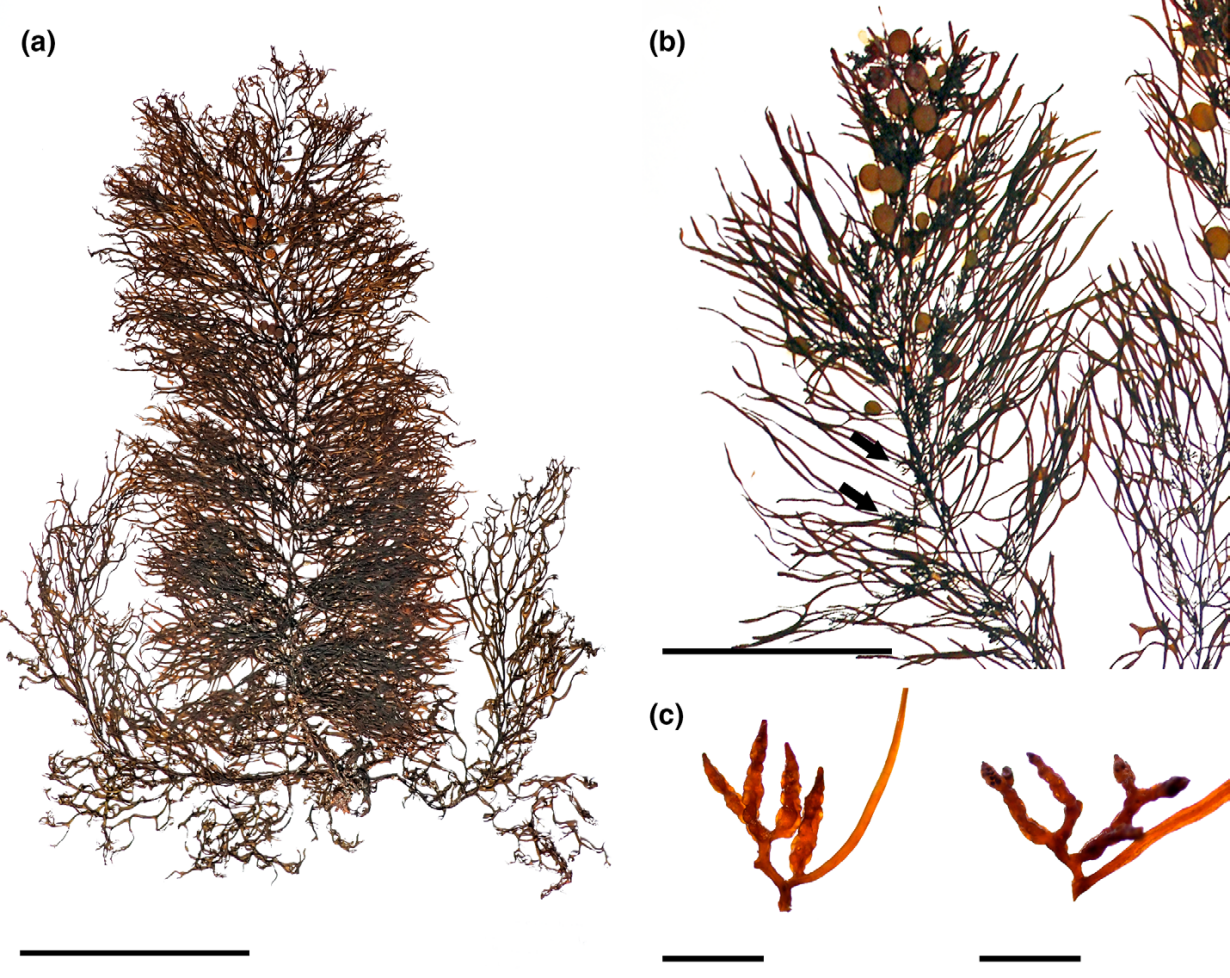
*Sargassum ramifolium*. (a) Habit of a well‐developed individual with three primary branches and secondary branches (TFC Phyc 16509). Scale bar = 10 cm. (b) Detail of a mature individual with abundant vesicles and receptacles (arrows; TFC Phyc 16518). Scale bar = 5 cm. (c) Detail simple to several times ramified receptacles. Scale bar = 3.5 mm. [Color figure can be viewed at wileyonlinelibrary.com]

**Type specimen.** L 4025287 (Naturalis Biodiversity Center: Leiden, The Netherlands)

**Type locality.** Brazil (*Ad oram brasiliae: Sellow*; Kützing, 1843, p. 362)

**Synonymy.** *Sargassum cymosum* var. *ramifolia* (Kützing) Grunow, 1916b, p. 141

**Representative material.** TFC Phyc 632, TFC Phyc 785, TFC Phyc 1070, TFC Phyc 2056, TFC Phyc 2250, TFC Phyc 4209–10, TFC Phyc 4628, TFC Phyc 16451–52, TFC Phyc 16481, TFC Phyc 16509–20

**Morphology.** Thalli up to 40 cm high, yellowish brown. Holdfast discoid‐conical up to 23 mm in diameter, bearing up to 10 main axes, up to 20 mm high and 3 mm in diameter, simple, with scars of fallen branches. Primary branches are coriaceous, smooth, terete, up to 375 mm long and 1 mm in diameter, producing secondary branches. Phylloids linear, up to six to seven times pinnate‐furcate, rarely simple, up to 75 mm long and 0.6–2(–3) mm wide, margins usually entire, rarely with a few scattered teeth, rounded to acute tips, percurrent midrib, and a short stalk 1–2 mm long. Vesicles spherical to slightly oblong, 3.5–7.2 mm in diameter, some with an apical spine‐like mucro; stalk terete, 2–5 mm long and 0.4 mm in diameter. Cryptostomata are scarce to absent, scattered over the surface of phylloids, often paired at both sides of the midrib, round to elliptical, 105–172 μm long, and 70–139 μm wide. Thalli monoecious, receptacles arranged in loose cymes on the secondary branches, terete, rarely simple, often two to three times irregularly forked, with wart‐like surface, up to 6.2 mm long and 0.8 mm wide, with a short, simple, or once branched sterile stalk.

**Distribution.** Widely distributed in tropical and subtropical waters of the NW Atlantic, with a few records in the tropical Atlantic coasts of Africa (Guiry & Guiry, 2024), and Canary Islands (John et al., 2004).

**Habitat.** Rare species, on rocky coasts, from low‐intertidal pools to the shallow subtidal up to 3 m depth, in wave‐exposed localities, growing in sympatry with other *Sargassum* species

**Remarks.** All previous collections of this species from the Canary Islands deposited in TFC and BCM are regarded as either *Sargassum cymosum* or *S. desfontainesii*. Originally described by Kützing (1843), *S. ramifolium* was synonymized to a variety of *S. cymosum* by Grunow (1916b), and later reinstated as a species by Taylor (1960). It has generally been regarded as a valid species since then. A note by Taylor on the type of *S. ramifolium* reinforces the idea that *S. cymosum*, *S. ramifolium*, and *S. stenophyllum* have historically been confused: “This surely is what I have been calling *S. cymosum stenophyllum*, incorrect because var. and sp. gbased on the same type; perhaps this is the better name to use. WRT” [sic]. Interestingly, John et al. (2004) included the Canary Islands in the distribution of *S. ramifolium* based on the first record of *S. cymosum* for the Canaries by Afonso‐Carrillo et al. (1983; TFC Phyc 632 and TFC Phyc 785); however, this species has not been included in recent checklists (Gallardo et al., 2016).

### *Sargassum stenophyllum* Martius, 1828 (Figure 14a–c)

**FIGURE 14:**
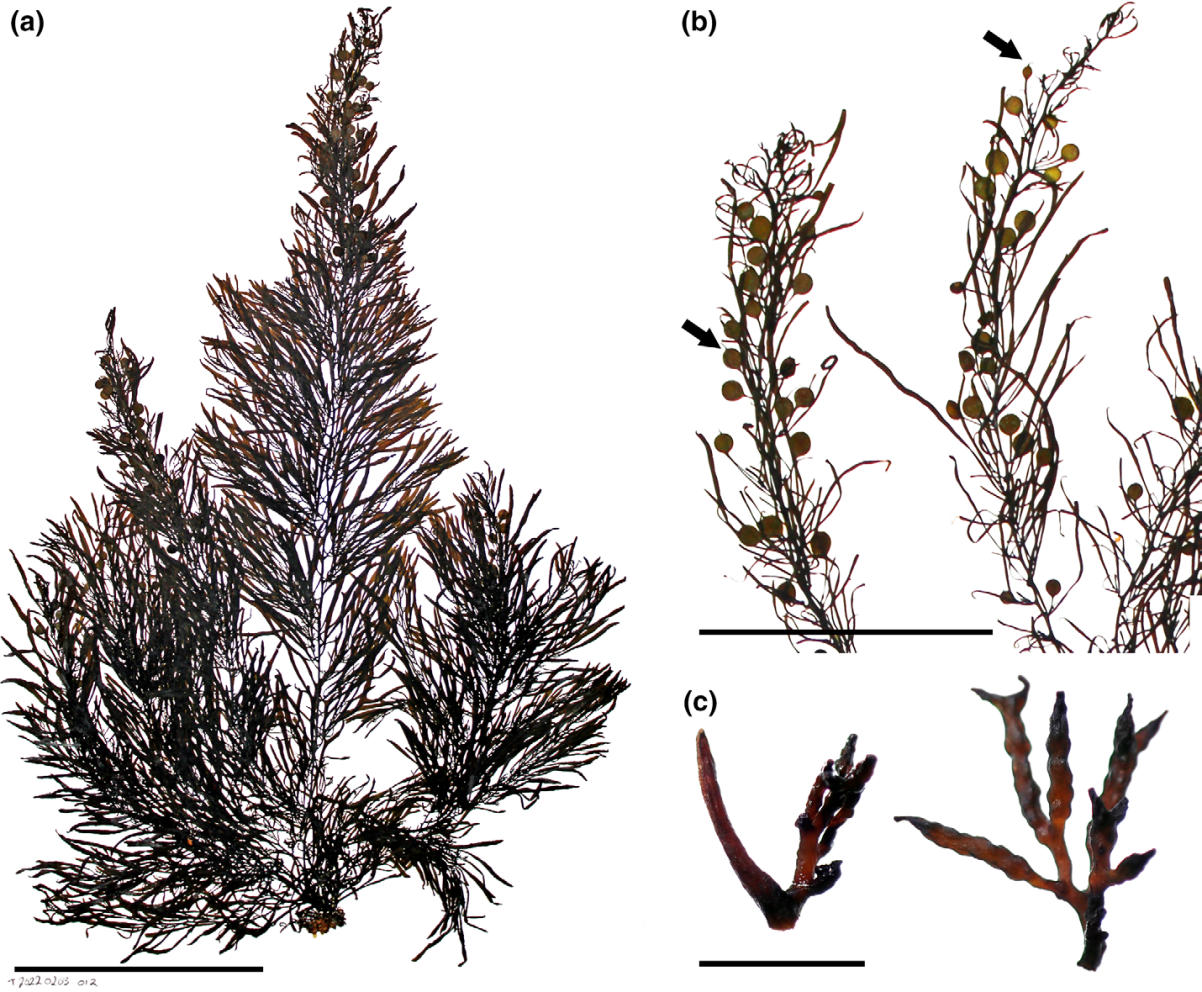
*Sargassum stenophyllum*. (a) Habit of a well‐developed individual with several primary branches, secondary branches, vesicles, and receptacles (TFC Phyc 16529). Scale bar = 10 cm. (b) Detail of a branch with vesicles terminated in apical spine‐like mucro (arrows; TFC Phyc 16460). Scale bar = 5 cm. (c) Detail of immature (left) and mature (right) receptacles. Scale bar = 3 mm. [Color figure can be viewed at wileyonlinelibrary.com]

**Type specimen.** Unknown

**Type locality.** São Paulo, Brazil (*Crescit in Oceano atlantico ad oras Provinciarum Bahiensis*, *Sebastianopolitanae et S. Pauli*; Martius, 1828, p. 8).

**Synonymy.** *Sargassum cymosum* var. *stenophyllum* (Martius) Grunow, 1916b, p. 138

**Representative material.** TFC Phyc 15459–60, TFC Phyc 16528–29

**Morphology.** Thalli up to 38 cm high, greenish brown. Holdfast discoid‐conical up to 25 mm in diameter, bearing up to six main axes, up to 12 mm high and 4 mm in diameter, simple, with scars of fallen branches. Primary branches are coriaceous, smooth, terete to slightly compressed, up to 353 mm long and 1.3 mm in diameter, producing secondary branches. Phylloids linear, rarely forked near the apices, up to 60 mm long and 0.6–1.5 (–2.4) mm wide, margins entire, acute to rounded tips, percurrent midrib, and a short stalk 1–2 mm long. Vesicles spherical to slightly oblong, 2.6–4.6 mm in diameter, some with an apical spine‐like mucro; stalk terete, 2–6(–11) mm long and 0.5 mm in diameter. Cryptostomata scarce to absent, scattered on the phylloid surfaces, often very close to the margins, elliptical to elongated 63–136 μm long and 30–98 μm wide. Thalli monoecious, receptacles arranged in loose cymes on the secondary branches, terete, rarely simple, often two to three times irregularly forked, with wart‐like surface, up to 5.7 mm long and 0.6 mm wide, with a short, simple, or once branched sterile stalk.

**Distribution.** Tropical western Atlantic (Camacho et al., 2015) and Canary Islands (this study)

**Habitat.** Rare, on rocky coasts, from low‐intertidal pools to the shallow subtidal up to 3 m depth, in wave‐exposed localities, growing sympatrically with other *Sargassum* species

**Remarks.** *Sargassum stenophyllum* was originally described by Martius (1828), but it has been historically regarded as a synonym of *S. cymosum*, similar to *S. ramifolium*, until it was recognized as an independent species by De Paula (1988). Examined material from the Canary Islands agrees with descriptions from the W Tropical Atlantic (Camacho et al., 2015) and the type locality in Brazil (De Paula, 1988), except for the dioecious character of the thalli. Some dubious records exist for the Eastern Atlantic (as *S. cymosum* var. *stenophylla*) attributed to Grunow (1916b), which were later regarded as a synonym of *S. vulgare* (Price et al., 1978). Also, Montagne's (1841) *S. fissifolium* could be related to this species, as he notes that it is extremely similar to some forms of *S. stenophyllum*.

### *Sargassum* sp. 1 (Figure 15a–d)

**FIGURE 15:**
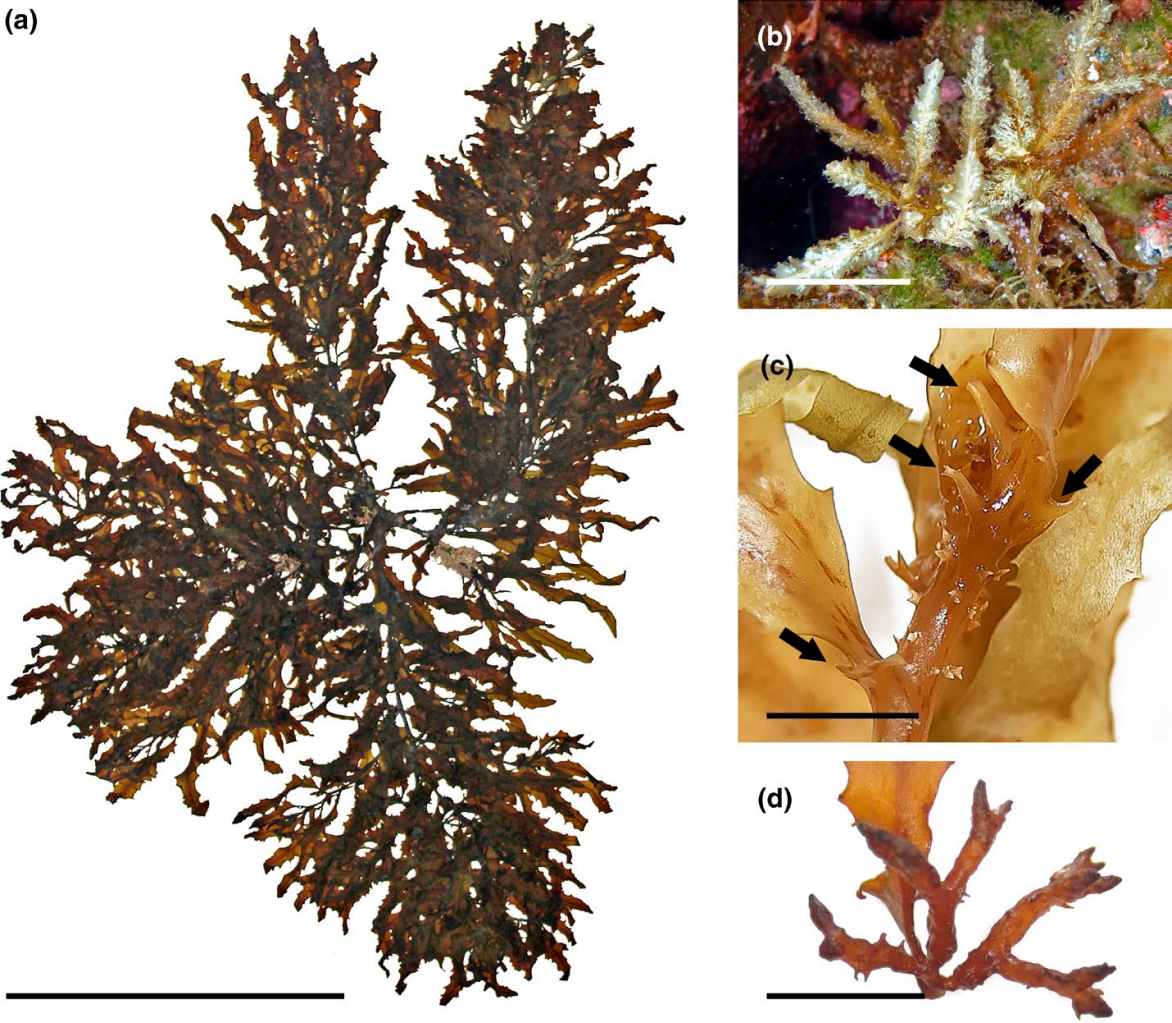
*Sargassum* sp. 1. (a) Habit of a well‐developed individual with crowded, serrate phylloids (TFC Phyc 16497). Scale bar = 10 cm. (b) Young individual in its natural environment, with dark and light markings on its phylloids (Loc. Las Teresitas, Tenerife, 05 May 2022). Scale bar = 3 cm (approx.). (c) Detail of a formalin‐preserved primary branch covered with spur‐like protuberances, and bases of phylloids with spines (arrows). Some dark markings can be seen in the phylloids and along the main branch. Scale bar = 1 cm (approx.). (d) Detail of a dichotomously branched receptacle with spines and spur‐like protuberances. Scale bar = 5 mm. [Color figure can be viewed at wileyonlinelibrary.com]

**Representative material.** TFC Phyc 845, TFC Phyc 4518, TFC Phyc 4761, TFC Phyc 5402, TFC Phyc 5596, TFC Phyc 5619, TFC Phyc 8626, TFC Phyc 11765, TFC Phyc 16463–66, TFC Phyc 16496–504, TFC Phyc 16541–44

**Morphology.** Thalli up to 30 cm high, light to dark brown, with a faint grayish iridescence underwater. Holdfast discoid‐conical up to 35 mm in diameter, bearing up to seven main axes, up to 17.5 mm high and 3.2 mm in diameter, simple, with scars of fallen branches. Primary branches are coriaceous, terete, up to 270 mm long and 2.6 mm in diameter, sometimes producing secondary branches, with spines and spur‐like protuberances on their surface, especially abundant in the distal third of the branches, smoother near the base. Phylloids crowded, with a stiff‐crisp texture when fresh, usually contorted or spirally twisted and with small dark and light marks on its surface, oval to lanceolate, simple to several times furcate, up to 60(–72) mm long and 3–6(–7.2) mm wide, tapering toward the base and apices, bases asymmetrical with one to four short spines, margins from entire to regularly dentate, obtuse to acute tips, percurrent midrib, sessile, or in very short stalks. Vesicles are rare, only on the uppermost parts of branches, spherical to oblong, 5–8 mm in diameter, smooth; stalk terete, 4–7.5 mm long and 0.6 mm in diameter. Cryptostomata are frequent, scattered on the phylloid surfaces, round to elliptical, 70–177 μm long, and 51–170 μm wide. Thalli monoecious, receptacles solitary in the axils of phylloids or vesicles of the secondary branches, terete, several times irregularly dichotomously branched, with abundant spines and spur‐like proliferations on its surface, up to 8.6 mm long and 1.1 mm wide, with a short sterile stalk.

**Distribution.** Canary Islands (this study)

**Habitat.** Abundant in wave‐exposed localities, growing in the lower intertidal and shallow subtidal, up to 6 m depth, living in sympatry with other *Sargassum* species

**Remarks.** This species somewhat resembles descriptions of *Sargassum polyceratium* Montagne (Ballantine et al., 2021; Chapman, 1963; Schnetter, 1976; Taylor, 1960); however, some diagnostic characters such as the presence of iridescence or the light/dark markings on the phylloids are not mentioned in W Atlantic material. A *Sargassum* species recently reported in the Mediterranean and regarded as *S. furcatum* showed these two characters but differed from the Canarian specimens in the branched (never simple) phylloids (Marletta et al., 2024). Chapman's (1963) description of *S. lendigerum* (Linnaeus) C. Agardh also bears similarity with the Canarian material, and it has been previously reported for the Canary Islands and other locations in the E Atlantic, for example, Gabon and Cape Verde (Price et al., 1978), yet most of these records have been merged under *S. vulgare* in recent checklists (John et al., 2004).

### *Sargassum* sp. 2 (Figure 16a,b)

**FIGURE 16:**
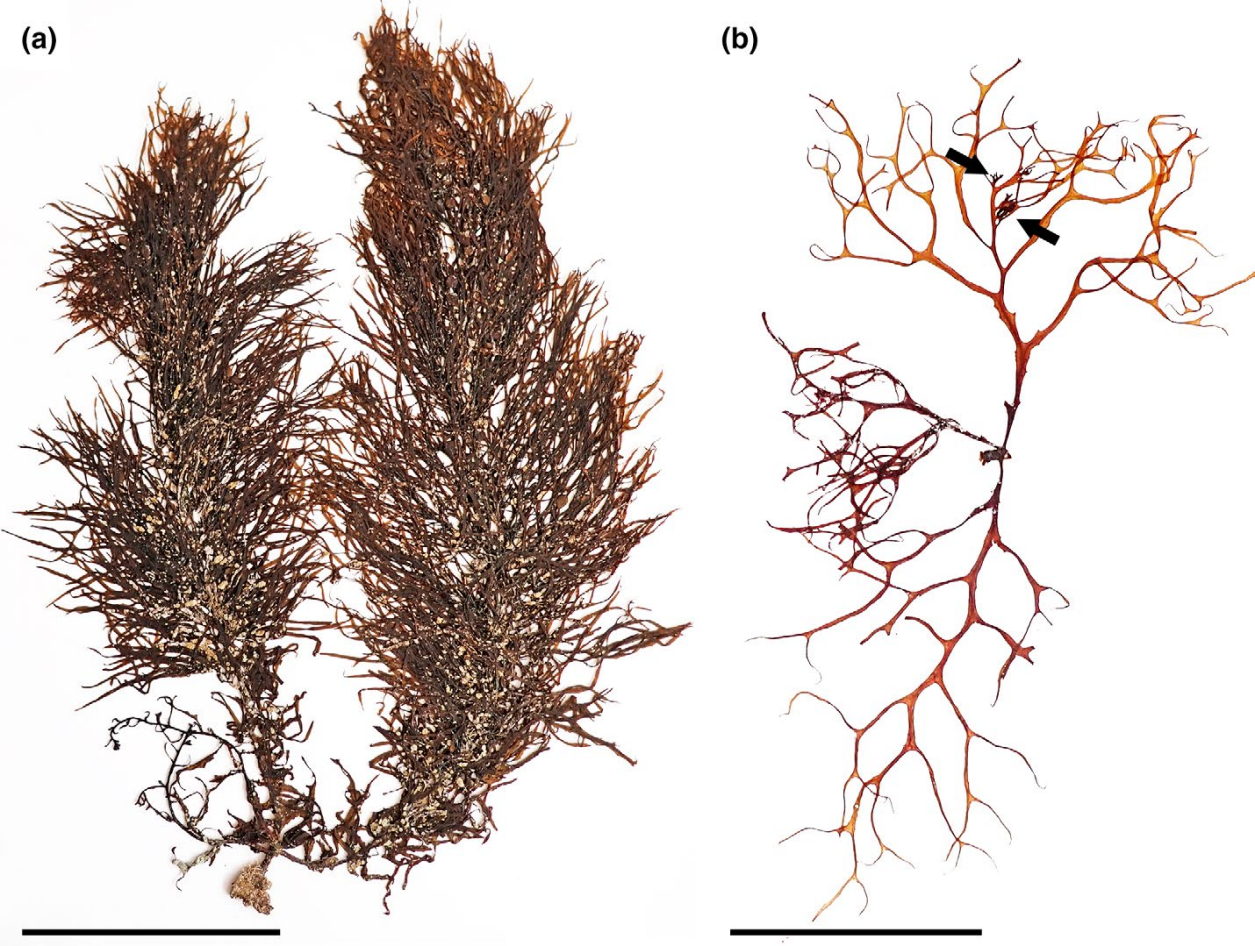
*Sargassum* sp. 2. (a) Habit of a developed individual (TFC Phyc 16563). Scale bar = 10 cm. (b) Young individual with three phylloids. Note the pinnate branching and progressive narrowing of the phylloids to hair‐like apices. Some aberrant undeveloped receptacles are borne in one of the phylloids (arrows; TFC Phyc 16489). Scale bar = 5 cm. [Color figure can be viewed at wileyonlinelibrary.com]

**Representative material.** TFC Phyc 16471, TFC Phyc 16483, TFC Phyc 16485–86, TFC Phyc 88–90, TFC Phyc 545.

**Morphology.** Thalli up to 30 cm high, light brown to brown. Holdfast discoid‐conical up to 20 mm in diameter, usually bearing one main axis, up to 25.9 mm high and 4.0 mm in diameter, simple, with scars of fallen branches. Primary branches are coriaceous, terete, up to 235 mm long and 1.4 mm in diameter, sometimes producing secondary branches, smooth throughout. Phylloids sparse, linear to linear‐lanceolate, narrow, several times furcate, with entire margins or with a few teeth in the proximal third of its length, up to 75(–100) mm long and 1.2–2.5(–3.2) mm wide, gradually tapering from the base to very thin, almost hair‐like apices, sessile or in very short stalks. Vesicles are rare, only present in well‐developed individuals, spherical to oblong, 5–6 mm in diameter, smooth; stalk terete, 3.2–4.6 mm long and 0.3 mm in diameter. Cryptostomata are frequent, scattered on the phylloid surfaces, elliptical, 74–163 μm long and 47–113 μm wide. Fertile thalli were not observed.

**Distribution.** Canary Islands (this study)

**Habitat.** Common in the subtidal, usually growing below 10 m depth, and often observed at shallower habitats, such as shaded crevices or on rocks partially covered with sediment

**Remarks.** Young individuals are abundant year round, characterized by primary branches 2–3 cm long and just a few phylloids longer than the branches, with densities of over 50 individuals · m^−2^ in some localities. These small individuals seem to have difficulty becoming adults, since well‐developed individuals are very rare throughout the year even in the same localities where young individuals are abundant.

### *Sargassum* sp. 3 (Figure 17a–c)

**FIGURE 17:**
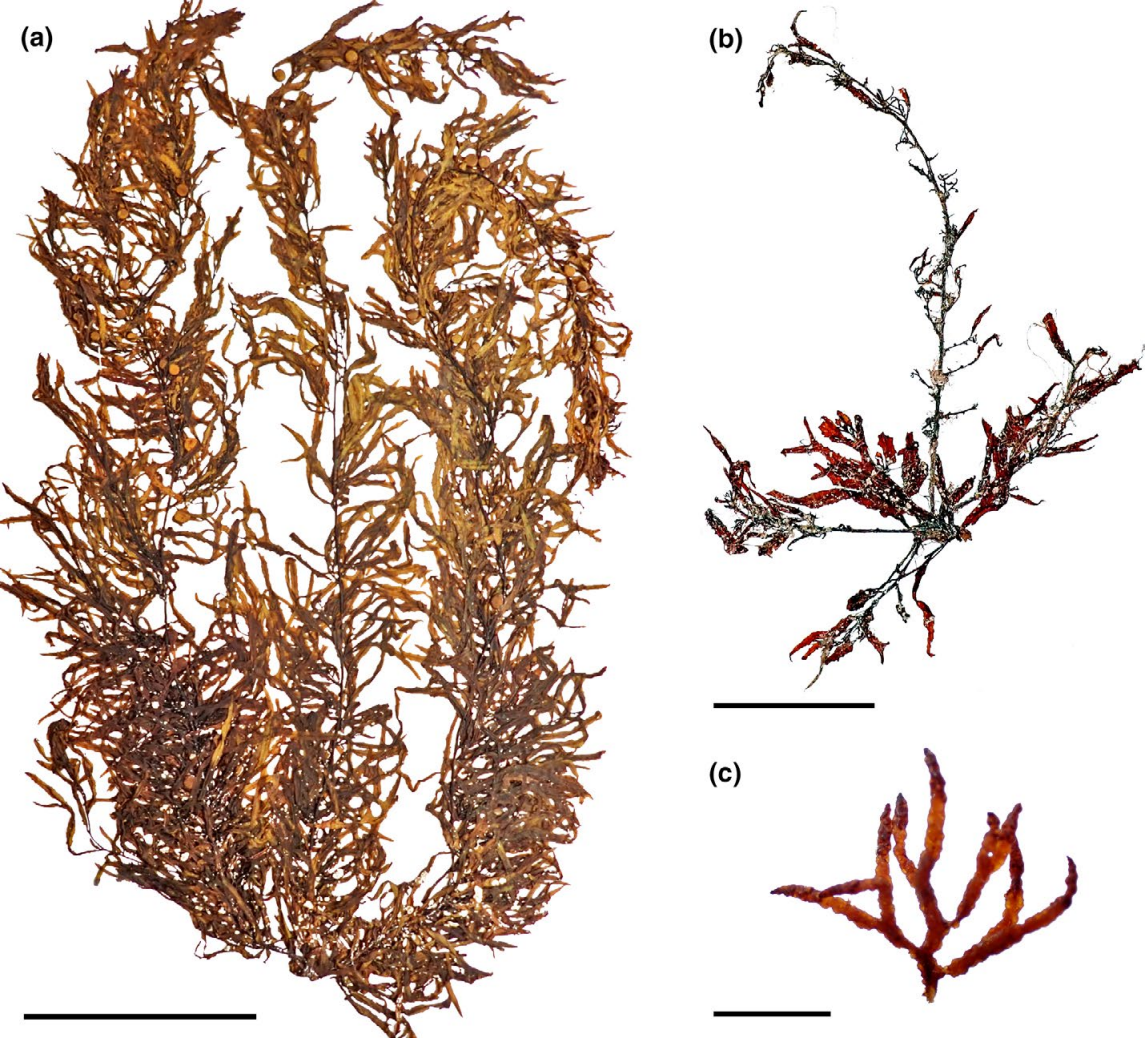
*Sargassum* sp. 3. (a) Habit of a well‐developed individual with several primary branches, secondary branches, abundant vesicles, and receptacles (TFC Phyc 16472). Scale bar = 10 cm. (b) Senescent individual with denuded primary branches, retaining some receptacles in the apical portion (TFC Phyc 16549). Scale bar = 5 cm. (c) Detail of long, furcate receptacles fertile from the base. Scale bar = 5 mm. [Color figure can be viewed at wileyonlinelibrary.com]

**Representative material.** TFC Phyc 16469–70, TFC Phyc 16472, TFC Phyc 16479, TFC Phyc 16546–55

**Morphology.** Thalli up to 60 cm high, brown. Holdfast discoid‐conical up to 25 mm in diameter, bearing one to three main axes, up to 19 mm high and 3 mm in diameter, simple, with scars of fallen branches. Primary branches are coriaceous, terete, up to 190 mm long and 1.4 mm in diameter, sometimes producing secondary branches, smooth throughout. Phylloids sparse, linear‐lanceolate, wide, simple to several times furcate, margins irregularly dentate, up to 55(–70) mm long and 2.5–3.9(–4.2) mm wide, gradually tapering to rounded apices, sessile or in very short stalks. Vesicles absent in all collected individuals. Cryptostomata frequent, scattered on the phylloid surfaces, round to elliptical 62–140 μm long and 45–102 μm wide. Thalli monoecious, receptacles arranged in the axils of phylloids or vesicles, solitary or clustered in loose racemes, terete, rarely simple, often several times irregularly dichotomously branched with a warty surface, up to 11 mm long and 1.1 mm wide, fertile throughout, without a sterile stalk.

**Habitat.** Frequent on rocky habitats at the shallow sublittoral, usually growing at <15 m depth

**Remarks.** This species somewhat resembles *Sargassum* sp. 2, but the phylloids of *Sargassum* sp. 3 are more regularly toothed along the entire margins and end in rounded apices. Additionally, *Sargassum* sp. 3 usually grows in shallower environments, although they can be observed sympatrically in a few sites. To some extent, specimens match morphologically those from the Mediterranean endemic species *S. trichocarpum* J.Agardh (Gómez‐Garreta, 2000; Hamel, 1939; Rodríguez‐Prieto et al., 2013), especially the characteristic long filiform receptacles, fertile from the base. Further analyses are needed to consider whether both entities are conspecific.

### Dubious records

Two other species of *Sargassum* have been reported for the Canary Islands, *Sargassum acinarium* (Linnaeus) Setchell and *Sargassum vulgare* C. Agardh, neither of which could be confirmed during this study. Both species carry taxonomic uncertainties beyond the scope of this work, and records can be partly explained by their nomenclatural complexity.

*Sargassum acinarium* has an extremely complex history as described in detail by Setchell (1933) and Silva et al. (1996), and its presence in the Canary Islands is somewhat doubtful. The first records came from Bory de Saint‐Vincent (1828) as *S. acinaria*, and he clearly stated that he did not refer to the Linnaean *Fucus acinarius* (=*S. linifolium*) from the Mediterranean. Børgesen (1926, p. 108) doubted the old records under the name *S. linifolium*, stating that “the true *S. linifolium*, according to Grunow, is only found in the Mediterranean Sea” when discussing *S. linifolium* var. *amygdalyfolium* collected in the Canary Islands by Vickers (1896), considering it a synonym of *S. vulgare*. Later, Gil‐Rodríguez et al. (1986) commented on the absence of more recent records. The absence of *S. acinarium* collections during the exhaustive field samplings of our study, the lack of herbarium material under this name, and its extremely taxonomic complexity make these early records doubtful and practically unverifiable. From our recent collections, *Sargassum* sp. 3 would be the closest to *S. acinarium* from the Mediterranean (Gómez‐Garreta, 2000; Hamel, 1939; Rodríguez‐Prieto et al., 2013), but the receptacles lack the characteristic long, ramified sterile stalks.

Likewise, *Sargassum vulgare* has a convoluted history. The name is considered illegitimate as it was superfluous when created by C. Agardh (Silva et al., 1996), and a proposal to conserve the name with a neotype from the Mediterranean (Israel) was rejected (Prud'homme van Reine, 2011; Ramon & Gil‐ad, 2007). Its taxonomic status is still unresolved, and the name has been applied to collections from tropical and subtropical coasts of the Atlantic, Indian, and Pacific Oceans (Guiry & Guiry, 2024). Recent works have regarded many records from the Indian and Pacific Oceans as misidentifications of other entities (Dixon & Huisman, 2015; Mattio et al., 2008, 2013, 2015), while Western Atlantic records remain uncertain (Camacho et al., 2015). Most of the material deposited in TFC under this name corresponds to *S. filipendula*, *S. flavifolium*, and *Sargassum* sp. 1 or consists of incomplete and/or young individuals that lack all the diagnostic characters needed to identify them with certainty. Until further studies clarify its circumscription, we have chosen to refrain from using this name.

## DISCUSSION

### Molecular diversity and phylogenetics

Our single‐gene and multi‐region phylogenetic analyses confirmed the low molecular diversity of *Sargassum* section *Sargassum* in the Atlantic Ocean, as all newly generated sequences clustered with sequences from the W Atlantic and the Mediterranean in polytomous clades with very low support and genetic distance, regardless of their morphological species assignation, their geographic distance, or the genetic markers used. Of the 10 genetic markers analyzed, only three (*cox*2, *cox*3, and *mtsp*) showed some nucleotide variation among the sequences from the Canary Islands, yet these differences were not species specific but instead unique or shared by multiple sequences irrespective of the morphological assignation. The phylogenetic results here are congruent with previous observations of *Sargassum* section *Sargassum* from W Atlantic based on the *cox*1, *cox*3, and *rbc*LS genes and ITS rRNA region that have also reported polytomous groupings of sequences from different species (Camacho et al., 2015; González‐Nieto et al., 2020).

Despite the low resolving power of these genetic markers in the Atlantic, some of them have been used in population genetics studies of *Sargassum* in the Pacific and Indian Oceans, in which several haplotypes were observed at local and regional scales for the different species studied (Chan et al., 2014; Cheang et al., 2010; Dumilag et al., 2022; Hu et al., 2017; Li et al., 2017; Ng et al., 2019; Uwai et al., 2009; Watanabe et al., 2019). Low levels of diversity, yet higher than our results, have also been reported in the subgenus *Bactrophycus* section *Halochloa* for Australian and Asian samples, which formed a group of poorly supported, polytomous clade of unclear affinities (Dixon et al., 2014). This extremely low genetic diversity seems restricted to the Atlantic *Sargassum* section *Sargassum* species.

Nevertheless, extremely low resolution in molecular studies of closely related species using single or multigene approaches is not unusual, and it has been reported previously in other algal genera. A single‐gene *cox*1 phylogeny of the Laminariacean genus *Macrocystis* showed extremely low diversity that supported the recognition of a single species (Macaya & Zuccarello, 2010); however, the use of whole‐genome sequencing techniques has uncovered genetic differences that challenge this view, as there might be at least two distinct species (Gonzalez et al., 2023). Likewise, species delimitation in *Fucus* has proven difficult, as species that are easily recognizable have almost identical sequences using traditional genetic markers such as the *cox*1, mt23S and *mtsp* genes and ITS1 and ITS2 rRNA regions (Coyer et al., 2006; Kucera & Saunders, 2008), as it is also the case in Atlantic *Sargassum*. Population genetics analyses have shown differences that support isolation and speciation between species in *Fucus* (Almeida et al., 2022; Bergström et al., 2005; Zardi et al., 2011), and recent studies on *F. vesiculosus* using restricted‐site associated DNA (RAD) sequencing confirmed the presence of a single clone previously considered to be different entities (Pereyra et al., 2023). The low genetic diversity in Atlantic *Sargassum* could be related to an incipient speciation due to the recent colonization of this basin, estimated to be 1.0 million year ago (mya) for the section *Sargassum* and as recent as ca. 0.4–0.2 mya for the clade comprising the Atlantic species (Yip et al., 2020). This recent colonization along with incomplete lineage sorting, gene flow, and potential hybridization among sympatric populations could explain these low levels of molecular diversity despite the high morphological diversity observed.

### Incongruence between morphology and phylogeny

Even though more than 30 species of *Sargassum* are recognized for the N Atlantic based on morphological grounds (Aouissi et al., 2018; John et al., 2004; Wynne, 2017), our results showed that Canarian specimens and almost all Atlantic species cannot be delimited based on the use of traditional genetic markers (*cox*3, *rbc*LS, and *mtsp* genes and the ITS2 rRNA region), nor with additional genes (*cox*2, *nad*6, *psb*C, *clp*C, *atp*B), as they form a single genetic entity. González‐Nieto et al. (2020) proposed that all sequences from 10 W Atlantic morphospecies belonged to the single species *Sargassum* cf. *cymosum*, and later Wynne (2022) corrected this to *S. natans*, adding another five species as synonyms of this extremely morphologically variable entity. Considering *cox*3 and *rbc*LS genes and ITS2 rRNA region sequences of samples from both sides of the Atlantic, varying from as low as 0% (identical) to a maximum of 1.5%, the Canarian morphospecies would fall within the same proposed single Atlantic species (i.e., *S. natans*), further expanding its morphological variability and geographic range to the whole tropical and temperate NE Atlantic, including the Mediterranean. This massive lumping of species would have consequences beyond taxonomy, as this species concept of *S. natans* would include species with clearly endemic distributions (as the NE Atlantic *S. desfontainesii*, *S. flavifolium*, and *S. orotavicum* or the Mediterranean *S. hornschuchii*, *S. ramentaceum*, and *S. trichocarpum*; Aouissi et al., 2018), distinct chemical compositions (Kergosien et al., 2024; Rosado‐Espinosa et al., 2020), and distinct ecology (Faga & Gurgel, 2024). Moreover, this approach could have drastic implications from a management perspective, as it would pool species considered a nuisance (*S. fluitans* and *S. natans*) because of their massive proliferations (Devault et al., 2021) with species that have high ecological relevance. Some of these species are threatened by the decline of their populations and are even under protection figures, for example, all the species within the Mediterranean basin are under the Barcelona Convention (Bernal‐Ibáñez et al., 2021; Gobierno de Canarias, 2010; Thibaut et al., 2016; UN Environment Programme, 2019). Furthermore, our results showed that when analyzing the additional set of genes *cox*2, *mtsp*, and *nad*6, there was some level of differentiation between E Atlantic *Sargassum* and the holopelagic *S. fluitans* and *S. natans*.

Therefore, we call for caution and have opted to take a conservative approach, not lumping the different morphospecies and considering as valid all entities until further molecular data are available. We consider that merging species would create more instability, and we anticipate that nuclear genome data will shed light on the phylogenetic relationships and population genetics of the genus *Sargassum* in the Atlantic Ocean.

## AUTHOR CONTRIBUTIONS

**Daniel Álvarez‐Canali:** Conceptualization (lead); data curation (lead); formal analysis (lead); funding acquisition (lead); investigation (lead); methodology (equal); resources (equal); visualization (lead); writing – original draft (lead); writing – review and editing (lead). **Marta Sansón:** Conceptualization (equal); funding acquisition (equal); investigation (equal); resources (equal); supervision (equal); validation (equal); writing – original draft (equal); writing – review and editing (supporting). **Carlos Sangil:** Conceptualization (supporting); funding acquisition (supporting); investigation (equal); resources (equal); supervision (equal); validation (supporting); writing – original draft (supporting); writing – review and editing (supporting). **Ana Tronholm:** Conceptualization (equal); data curation (supporting); formal analysis (equal); funding acquisition (lead); investigation (equal); methodology (equal); resources (equal); supervision (equal); validation (equal); visualization (supporting); writing – original draft (equal); writing – review and editing (lead).

## FUNDING INFORMATION

This work is part of D.A.‐C. PhD thesis, co‐funded by the Canarian Agency for Research, Innovation and Information Society of the Ministry of Universities, Science and Innovation and Culture (formerly Ministry of Economy, Knowledge and Employment) of the Canarian Government; by the European Social Fund (ESF) Canary Islands Integrated Operational Program 2014–2020, Axis 3, Priority Theme 74 (85%) [grants TESIS2020010084 and EST2022010033]; and by the European Social Fund Plus (ESF+) Canary Islands Integrated Operational Program 2021–2027, Objective 4, Priority 8, Specific Objective 4.6/4f (85%) [grant EST2023010006].

## Supporting information


**Figure S1.** Maximum likelihood phylogenetic tree of *Sargassum* based in *cox*3 sequences. Values at the nodes indicate bootstrap support (left) and posterior probability (right). Values below 60/0.6 are not shown. Asterisk (*) indicates full support. Sequences generated in this study in bold.

**Figure S2.** Maximum likelihood phylogenetic tree of *Sargassum* based in ITS2 rRNA region sequences. Values at the nodes indicate bootstrap support (left) and posterior probability (right). Values below 60/0.6 are not shown. Asterisk (*) indicates full support. Sequences generated in this study in bold.

**Figure S3.** Maximum likelihood phylogenetic tree of *Sargassum* based in *rbc*LS gene sequences. Values at the nodes indicate bootstrap support (left) and posterior probability (right). Values below 60/0.6 are not shown. Asterisk (*) indicates full support. Sequences generated in this study in bold.

**Table S1.** Taxa included in the molecular analyses of the *cox*3 and *rbc*LS genes and ITS rRNA region, with collecting data, references and GenBank Accession No. n.d.: no data available.

**Table S2.** Taxa included in the molecular analyses of the mitochondrial spacer (*mtsp*), with the collecting data, references and GenBank Accession No. n.d.: no data available.

**Table S3.** Taxa included in the molecular analyses of the protein‐coding mitochondrial genes (*cox*2, extended *cox*3, *nad*6), with collecting data, references and GenBank Accession No. n.d.: no data available.

**Table S4.** Taxa included in the molecular analyses of the protein‐coding chloroplastic genes (*atp*B, *clp*C, *psb*C), with collecting data, references and GenBank Accession No. n.d.: no data available.

## Data Availability

Sequence data available on GenBank, accessions OR786495–OR786634 and OR786495–OR786634 (see Tables [Link], [Link]).
