# Supplementary figures and images for: Multigene phylogenetics of Sargassum (Phaeophyceae) revealed low molecular diversity in contrast to high morphological variability in the NE Atlantic Ocean
Source: J Phycol. 2024 Oct 26;60(6):1528–56. doi: 10.1111/jpy.13517 (PMC11670286; doi:10.1111/jpy.13517)

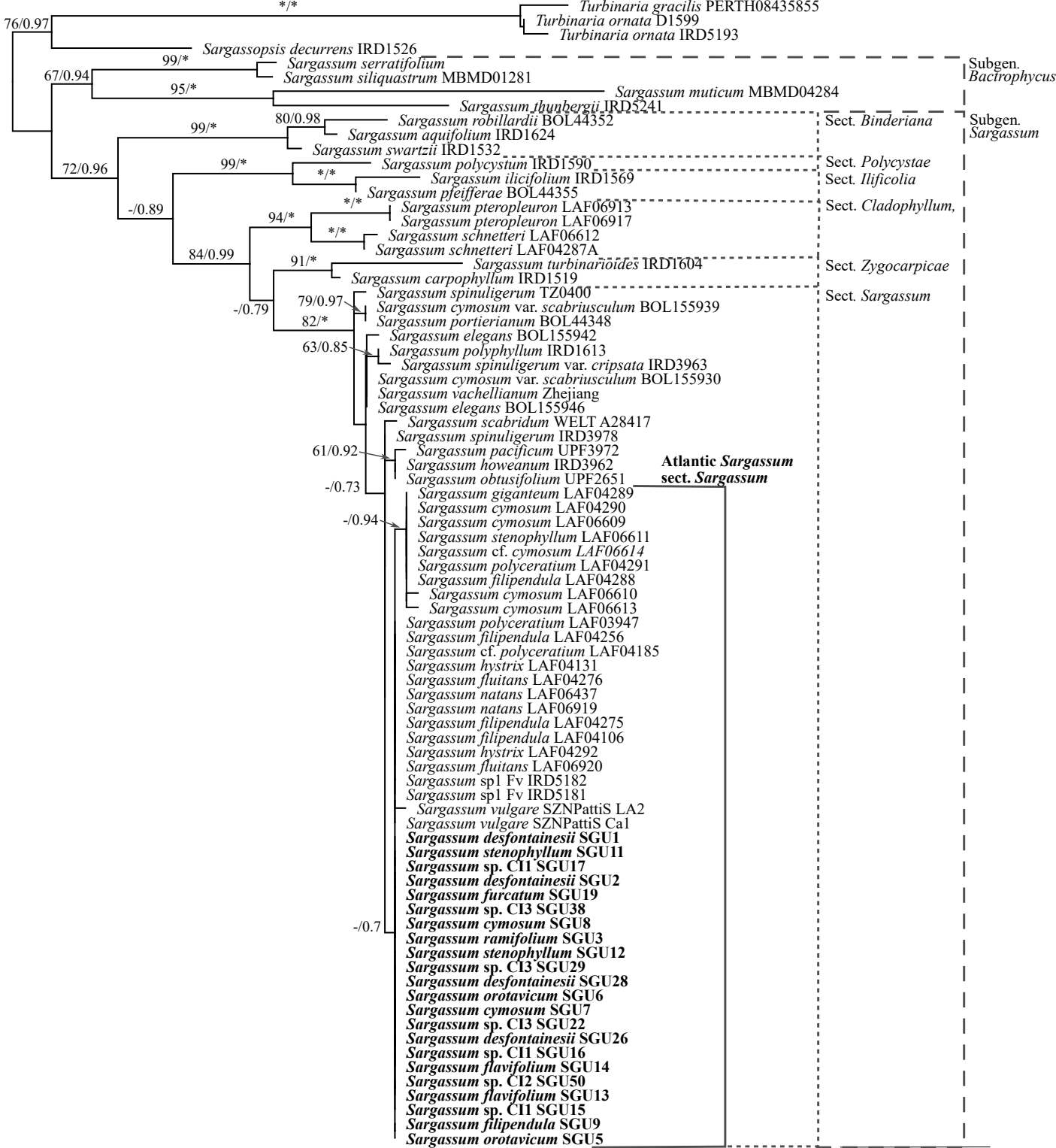

Supplement: Supplementary file 1 — Figure S1. Maximum likelihood phylogenetic tree of Sargassum based in cox3 sequences. Values at the nodes indicate bootstrap support (left) and posterior probability (right). Values below 60/0.6 are not shown. Asterisk (*) indicates full support. Sequences generated in this study in bold. [file JPY-60-1528-s003.pdf]

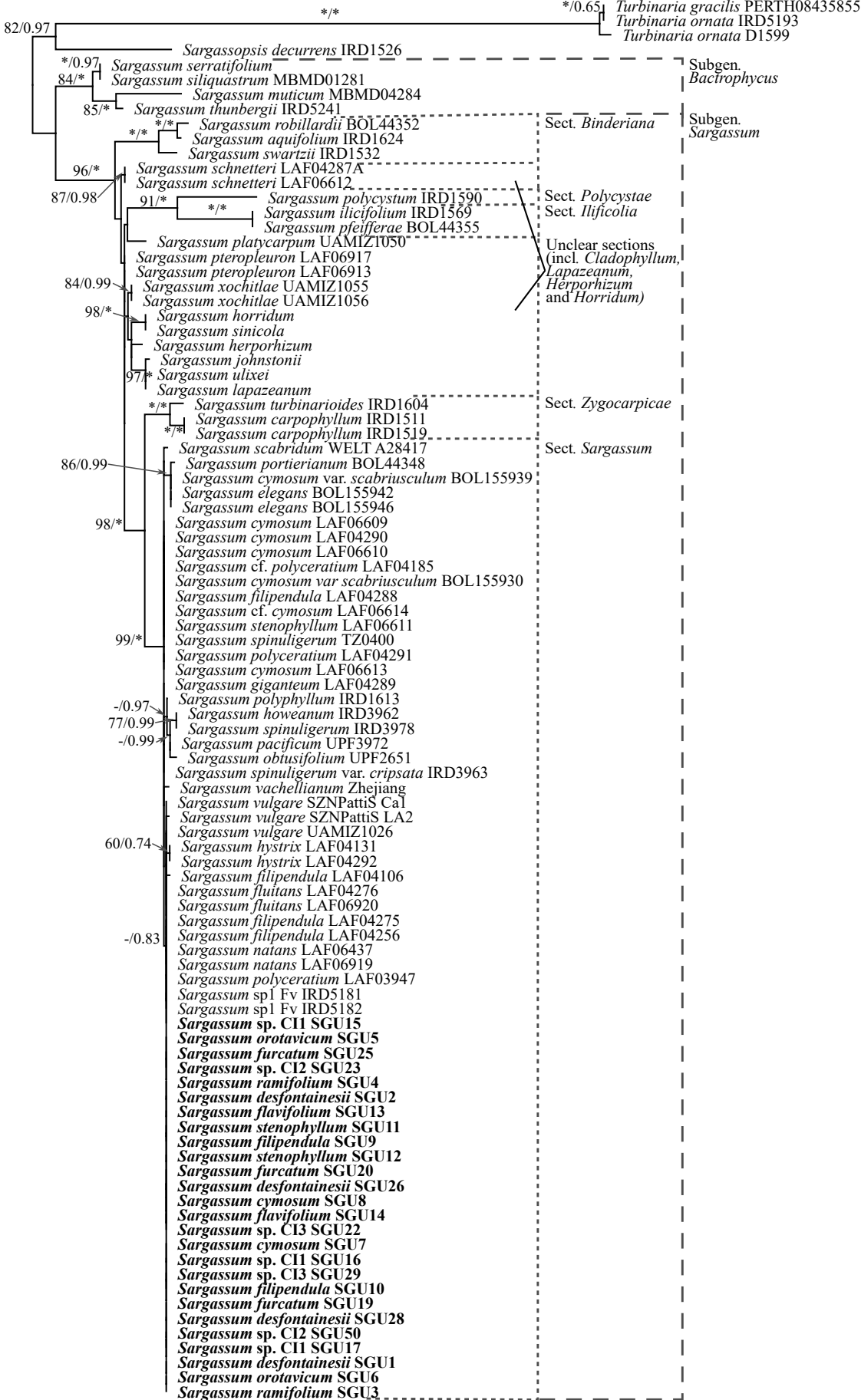

Supplement: Supplementary file 2 — Figure S2. Maximum likelihood phylogenetic tree of Sargassum based in ITS2 rRNA region sequences. Values at the nodes indicate bootstrap support (left) and posterior probability (right). Values below 60/0.6 are not shown. Asterisk (*) indicates full support. Sequences generated in this study in bold. [file JPY-60-1528-s002.pdf]

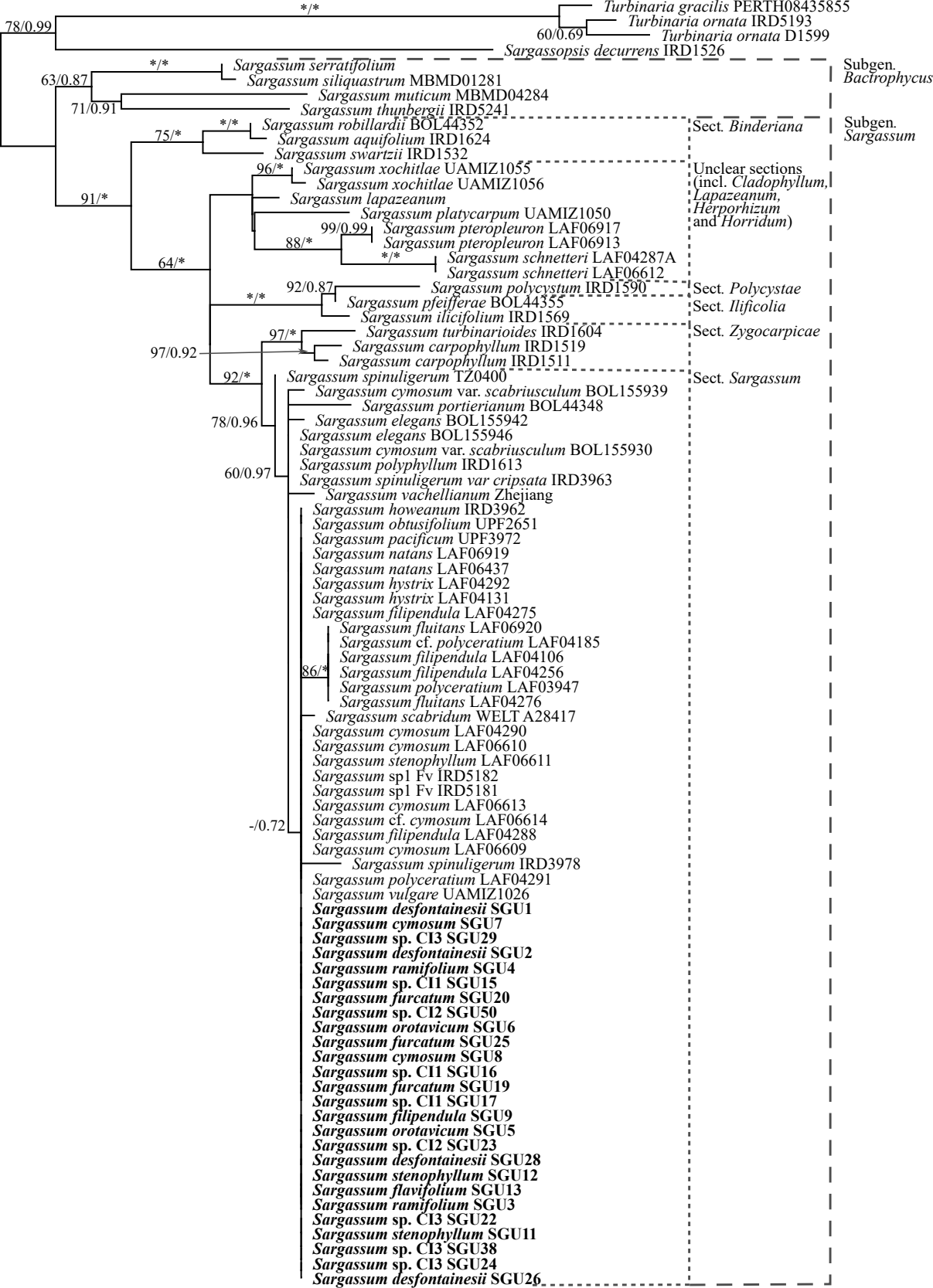

Supplement: Supplementary file 3 — Figure S3. Maximum likelihood phylogenetic tree of Sargassum based in rbcLS gene sequences. Values at the nodes indicate bootstrap support (left) and posterior probability (right). Values below 60/0.6 are not shown. Asterisk (*) indicates full support. Sequences generated in this study in bold. [file JPY-60-1528-s005.pdf]
