# Supplementary material for: Multigene phylogenetics of Sargassum (Phaeophyceae) revealed low molecular diversity in contrast to high morphological variability in the NE Atlantic Ocean
Source: J Phycol. 2024 Oct 26;60(6):1528–56. doi: 10.1111/jpy.13517 (PMC11670286; doi:10.1111/jpy.13517)
Supplement: Supplementary file 4 — Table S1. Taxa included in the molecular analyses of the cox3 and rbcLS genes and ITS rRNA region, with collecting data, references and GenBank Accession No. n.d.: no data available. [file JPY-60-1528-s007.docx]

| **Table S1.** Taxa included in the molecular analyses of the main genes (*cox*3, *rbc*LS genes and ITS2 rRNA region), with collecting data, references and GenBank Accession No. n.d.: no data available. | | | | | | |
| --- | --- | --- | --- | --- | --- | --- |
| **Organism** | **Specimen ID/Voucher** | **Collection site; Collection Date; Collector** | **References** | **GenBank Accession No.** | | |
|  |  |  |  | ***cox*3** | **ITS-2** | ***rbc*LS** |
| *Sargassum cymosum* | SGU7/  TFCPhyc16455 | Spain: Altagay, Punta Hidalgo, Tenerife, Canary Islands; 30-Jan-2022; D. Alvarez-Canali | This study | **OR786517** | **OR799833** | **OR786615** |
| *Sargassum cymosum* | SGU8/  TFCPhyc16456 | Spain: Punta Brava, Tenerife, Canary Islands; 03-Feb-2022; D. Alvarez-Canali | This study | **OR786518** | **OR799834** | **OR786616** |
| *Sargassum desfontainesii* | SGU1/  TFCPhyc16449 | Spain: La Laja, El Hierro, Canary Islands; 09-Feb-2020; D. Alvarez-Canali | This study | **OR786511** | **OR799827** | **OR786609** |
| *Sargassum desfontainesii* | SGU2/  TFCPhyc16450 | Spain: Altagay, Punta Hidalgo, Tenerife, Canary Islands; 30-Jan-2022; D. Alvarez-Canali | This study | **OR786512** | **OR799828** | **OR786610** |
| *Sargassum desfontainesii* | SGU26/  TFCPhyc16474 | Spain: La Salemera, La Palma, Canary Islands; 18-Feb-2022; D. Alvarez-Canali | This study | **OR786531** | **OR799849** | **OR786630** |
| *Sargassum desfontainesii* | SGU28/  TFCPhyc16476 | Spain: Órzola, Lanzarote, Canary Islands; 02-Apr-2022; D. Alvarez-Canali | This study | **OR786532** | **OR799850** | **OR786631** |
| *Sargassum filipendula* | SGU9/  TFCPhyc16457 | Spain: Altagay, Punta Hidalgo, Tenerife, Canary Islands; 30-Jan-2022; D. Alvarez-Canali | This study | **OR786519** | **OR799835** | **OR786617** |
| *Sargassum filipendula* | SGU10/  TFCPhyc16458 | Spain: Punta Hidalgo, Tenerife, Canary Islands; 29-Oct-2019; D. Alvarez-Canali | This study | **OR786520** | **OR799836** | - |
| *Sargassum flavifolium* | SGU13/  TFCPhyc16461 | Spain: Boca Cangrejo, Tenerife, Canary Islands; 25-Jan-2022; D. Alvarez-Canali | This study | **OR786523** | **OR799839** | **OR786620** |
| *Sargassum flavifolium* | SGU14/  TFCPhyc16462 | Spain: La Salemera, La Palma, Canary Islands; 23-Jun-2021; D. Alvarez-Canali | This study | **OR786524** | **OR799840** | - |
| *Sargassum furcatum* | SGU19/  TFCPhyc16467 | Spain: Playa Chica, Lanzarote, Canary Islands; 30-Mar-2022; D. Alvarez-Canali | This study | **OR786528** | **OR799844** | **OR786624** |
| *Sargassum furcatum* | SGU20/  TFCPhyc16468 | Spain: Playa Nogales, La Palma, Canary Islands; 22-Jun-2021; D. Alvarez-Canali | This study | **OR786529** | **OR799845** | **OR786625** |
| *Sargassum furcatum* | SGU25/  TFCPhyc16473 | Spain: La Barranquera, Tenerife, Canary Islands; 31-Jan-2022; D. Alvarez-Canali | This study | - | **OR799848** | **OR786629** |
| *Sargassum orotavicum* | SGU5/  TFCPhyc16453 | Spain: Punta Brava, Tenerife, Canary Islands; 07-Oct-2021; D. Alvarez-Canali | This study | **OR786515** | **OR799831** | **OR786613** |
| *Sargassum orotavicum* | SGU6/  TFCPhyc16454 | Spain: Punta Brava, Tenerife, Canary Islands; 03-Feb-2022; D. Alvarez-Canali | This study | **OR786516** | **OR799832** | **OR786614** |
| *Sargassum ramifolium* | SGU3/  TFCPhyc16451 | Spain: La Laja, El Hierro, Canary Islands; 29-Feb-2020; D. Alvarez-Canali | This study | **OR786513** | **OR799829** | **OR786611** |
| *Sargassum ramifolium* | SGU4/  TFCPhyc16452 | Spain: Altagay, Punta Hidalgo, Tenerife, Canary Islands; 30-Jan-2022; D. Alvarez-Canali | This study | **OR786514** | **OR799830** | **OR786612** |
| *Sargassum* sp. CI1 | SGU15/  TFCPhyc16463 | Spain: Playa Chica, Lanzarote, Canary Islands; 30-Mar-2022; D. Alvarez-Canali | This study | **OR786525** | **OR799841** | **OR786621** |
| *Sargassum* sp. CI1 | SGU16/  TFCPhyc16464 | Spain: La Barranquera, Tenerife, Canary Islands; 31-Jan-2022; D. Alvarez-Canali | This study | **OR786526** | **OR799842** | **OR786622** |
| **Table S1** (continued) |  |  |  |  |  |  |
| *Sargassum* sp. CI1 | SGU17/  TFCPhyc16465 | Spain: Punta Hidalgo, Tenerife, Canary Islands; 24-Jan-2022; D. Alvarez-Canali | This study | **OR786527** | **OR799843** | **OR786623** |
| *Sargassum* sp. CI2 | SGU23/  TFCPhyc16471 | Spain: Cuevas Coloradas, Montaña Clara, Lanzarote, Canary Islands; 22-Sep-2020; D. Alvarez-Canali | This study | - | **OR799847** | **OR786627** |
| *Sargassum* sp. CI2 | SGU50/  TFCPhyc16483 | Spain: Montaña Amarilla, La Graciosa, Lanzarote, Canary Islands; 14-Nov-2019; D. Alvarez-Canali | This study | **OR786535** | **OR799852** | **OR786634** |
| *Sargassum* sp. CI3 | SGU22/  TFCPhyc16470 | Spain: Alegranza, Lanzarote, Canary Islands; 22-Sep-2020; D. Alvarez-Canali | This study | **OR786530** | **OR799846** | **OR786626** |
| *Sargassum* sp. CI3 | SGU24/  TFCPhyc16472 | Spain: Cuevas Coloradas, Montaña Clara, Lanzarote, Canary Islands; 24-Mar-2021; D. Alvarez-Canali | This study | - | - | **OR786628** |
| *Sargassum* sp. CI3 | SGU29/  TFCPhyc16477 | Spain: Órzola, Lanzarote, Canary Islands; 02-Apr-2022; D. Alvarez-Canali | This study | **OR786533** | **OR799851** | **OR786632** |
| *Sargassum* sp. CI3 | SGU38/  TFCPhyc16479 | Spain: Punta Fariones, Lanzarote, Canary Islands; 20-Jul-2020; D. Alvarez-Canali | This study | **OR786534** | - | **OR786633** |
| *Sargassum stenophyllum* | SGU11/  TFCPhyc16459 | Spain: Punta Brava, Tenerife, Canary Islands; 03-Feb-2022; D. Alvarez-Canali | This study | **OR786521** | **OR799837** | **OR786618** |
| *Sargassum stenophyllum* | SGU12/  TFCPhyc16460 | Spain: La Barranquera, Tenerife, Canary Islands; 31-Jan-2022; D. Alvarez-Canali | This study | **OR786522** | **OR799838** | **OR786619** |
| *Sargassum aquifolium* | IRD1624 | New Caledonia: Ile Nouaré; Oct-2005; L. Mattio | Mattio et al., 2010 | EU882242 | EU882253 | EU882263 |
| *Sargassum carpophyllum* | IRD1519 | New Caledonia: Porc Epic Is.; Oct-2005; L. Mattio | Mattio et al., 2008; 2009 | EU833417 | EU100798 | EU100805 |
| *Sargassum carpophyllum* | IRD1511 | New Caledonia: Feycinet Is.; Jul-2005; L. Mattio | Mattio et al., 2008; 2009 | EU833415 | EU100797 | EU100804 |
| *Sargassum* cf. *cymosum* | LAF06614 | Colombia: Playa Blanca, Magdalena: May-2009; O. Camacho | Camacho et al., 2015 | KF437928 | KF437944 | KF437961 |
| *Sargassum* cf. *polyceratium* | LAF04185 | Puerto Rico: Caleta de cabullones, Ponce; Sep-2002; O. Camacho | Camacho et al., 2015 | KP064344 | KM461682 | KP064357 |
| *Sargassum cymosum* | LAF06613 | Colombia: Neguanje (TNNP), Magdalena; May-2009; O. Camacho | Camacho et al., 2015 | KF437927 | KF437943 | KF437960 |
| *Sargassum cymosum* | LAF06610 | Colombia: Concha (TNNP), Magdalena; Apr-2009; O. Camacho | Camacho et al., 2015 | KF437924 | KF437940 | KF437957 |
| *Sargassum cymosum* | LAF06609 | Colombia: Concha (TNNP), Magdalena; Apr-2009; O. Camacho | Camacho et al., 2015 | KF437923 | KF437939 | KF437956 |
| *Sargassum cymosum* | LAF04290 | Colombia: Granate (TNNP), Magdalena; Apr-2009; O. Camacho | Camacho et al., 2015 | KF437920 | KF437936 | KF437953 |
| *Sargassum cymosum* var. *scabriusculum* | BOL155939 | South Africa: Bhanga Nek, KwaZulu-Natal; Oct-2013; L. Mattio, R. Anderson, J.J. Bolton | Mattio et al., 2015 | KP720469 | KP720501 | KP720536 |
| *Sargassum cymosum* var. *scabriusculum* | BOL155930 | South Africa: Kalk Bay, Western Cape; Nov-2010; L. Mattio, R. Anderson, J.J. Bolton | Mattio et al., 2015 | KP720466 | KP720495 | KP720531 |
| *Sargassum elegans* | BOL155942 | South Africa: St. Lucia, KwaZulu Natal; Mar-2011; L. Mattio | Mattio et al., 2015 | KP720472 | KP720504 | KP720539 |
| *Sargassum elegans* | BOL155946 | South Africa: Hluleka, Eastern Cape; Aug-2013; R. Anderson, C. Boothroyd, D. Kemp | Mattio et al., 2015 | KP720474 | KP720506 | KP720540 |
| **Table S1** (continued) |  |  |  |  |  |  |
| *Sargassum filipendula* | LAF04288 | Colombia: Punta La Loma, Magdalena; May-2009; O. Camacho | Camacho et al., 2015 | KF437922 | KF437925 | KF437955 |
| *Sargassum filipendula* | LAF04275 | United States: Masonboro, North Carolina; May-2011; O. Camacho | Camacho et al., 2015 | KP064334 | KM461673 | KP064349 |
| *Sargassum filipendula* | LAF04106 | Panama: Zapatilla Cay; Aug-2010; O. Camacho | Camacho et al., 2015 | KP064332 | KM461671 | KP064347 |
| *Sargassum filipendula* | LAF04256 | Panama: Flat Rock Beach, Bocas del Toro; Aug 2010; S. Fredericq | Camacho et al., 2015 | KP064333 | KM461672 | KP064348 |
| *Sargassum fluitans* | LAF04276 | United States: Fort Fisher, North Carolina; May 2011; O. Camacho | Camacho et al., 2015 | KP064335 | KM461674 | KP064350 |
| *Sargassum fluitans* | LAF06920 | United States: Holly Beach, Gulf of Mexico; Jun-2014; O. Camacho | Camacho et al., 2015 | KP064336 | KM461675 | KP064351 |
| *Sargassum giganteum* | LAF04289 | Colombia: Granate (TNNP), Magdalena; Apr-2009; O. Camacho | Camacho et al., 2015 | KP064337 | KF437947 | - |
| *Sargassum herporhizum* | SPM27 | Mexico: San Pedro Martin Is.; May-2011; n.d. | Andrade-Sorcia et al., 2014 | - | JX560132 | - |
| *Sargassum horridum* | BS3 | Mexico: Piedras Coloradas; Apr-2010; n.d. | Andrade-Sorcia et al., 2014 | - | JX560122 | - |
| *Sargassum howeanum* | IRD3962 | New Caledonia: Ouano; Jun-2005; L. Mattio | Mattio & Payri, 2009 | FJ170411 | FJ170438 | FJ170384 |
| *Sargassum hystrix* | LAF04131 | United States: Geyer Bank, Gulf of Mexico; Sep-2011; E. Hickerson | Camacho et al., 2015 | KP064339 | KM461677 | KP064353 |
| *Sargassum hystrix* | LAF04292 | United States: Geyer Bank, Gulf of Mexico; Sep-2011; E. Hickerson | Camacho et al., 2015 | KP064338 | KM461676 | KP064352 |
| *Sargassum ilicifolium* | IRD1569 | Fiji: Makuluva Is.; Apr-2007; L. Mattio | Mattio et al., 2010 | EU833403 | EU833437 | EU833469 |
| *Sargassum johnstonii* | IPA3 | Mexico: Miramar Is.; May-2010; n.d. | Andrade-Sorcia et al., 2014 | - | JX560129 | - |
| *Sargassum lapazeanum* | 1PGPS4 | Mexico: Punta Mechudo; n.d.; n.d. | Andrade-Sorcia et al., 2014 | - | JX560127 | - |
| *Sargassum lapazeanum* | n.d. | Mexico: Baja; n.d.; R. Scrosati | Phillips et al., 2005 | - | - | AY256965 |
| *Sargassum muticum* | MBMD04284 | China: Nanhuangcheng Is.; Jun-2016; n.d. | Huang et al., 2017 | KY411087 | KY411113 | KY432492 |
| *Sargassum natans* | LAF06919 | United States: Holly Beach, Gulf of Mexico; Jun-2014; O. Camacho | Camacho et al., 2015 | KP064340 | KM461678 | KP064354 |
| *Sargassum natans* | LAF06437 | United States: Offshore LA, Gulf of Mexico; Jun-2014; O. Camacho | Camacho et al., 2015 | KP064341 | KM461679 | KP064355 |
| *Sargassum obtusifolium* | UPF2651 | French Polynesia: Rapa, Australs Is.; Nov-2002; C. Payri | Mattio et al., 2008; 2010 | EU100830 | EU100785 | EU100819 |
| *Sargassum pacificum* | UPF3972 | French Polynesia: Raiatea, Society Is.; Mar-2003; C. Payri, V. Stiger-Pouvreau | Mattio et al., 2008; 2010 | EU100828 | EU100784 | EU100813 |
| *Sargassum pfeifferae* | BOL44355 | Mauritius: Flic en Flac; Jun-2011; L. Mattio | Mattio et al., 2013 | KF413716 | KF413700 | KF413687 |
| *Sargassum platycarpum* | UAMIZ1050 | Mexico: Punta Chiquero, Quintana Roo; May-2013; D. González Nieto & L.E. Mateo-Cid | González-Nieto et al., 2020 |  | MT465426 | MT470867 |
| **Table S1** (continued) |  |  |  |  |  |  |
| *Sargassum polyceratium* | LAF04291 | Colombia: Neguanje (TNNP), Magdalena; May-2009; O. Camacho | Camacho et al., 2015 | KF437926 | KF437942 | KF437959 |
| *Sargassum polyceratium* | LAF03947 | Panama: Rio Cañaveral; Aug-2010; O. Camacho | Camacho et al., 2015 | KP064343 | KM461681 | KP064356 |
| *Sargassum polycystum* | IRD1590 | Fiji: Kiuva Reef; May-2007; L. Mattio | Mattio et al., 2010 | EU833410 | EU833421 | EU833472 |
| *Sargassum polyphyllum* | IRD1613 | New Caledonia: Maître Is.,; Sep-2005; L. Mattio | Mattio et al., 2009 | EU833385 | EU833424 | EU833458 |
| *Sargassum portierianum* | BOL44348 | Mauritius: Flic en Flac; Jun-2011; L. Mattio | Mattio et al., 2013 | KF413715 | KF413704 | KF413685 |
| *Sargassum pteropleuron* | LAF06913 | United States: Newfound Harbor Key, Florida; Apr-2014; O. Camacho | Camacho et al., 2015 | KP064345 | KM461683 | KP064358 |
| *Sargassum pteropleuron* | LAF06917 | United States: Sommerland Key, Florida; Apr-2014; O. Camacho | Camacho et al., 2015 | KP064346 | KM461684 | KP064359 |
| *Sargassum robillardii* | BOL44352 | Mauritius: Flic en Flac; Jun-2011; L. Mattio | Mattio et al., 2013 | KF413711 | KF413697 | KF413684 |
| *Sargassum scabridum* | WELT A28417 | New Zealand: Auckland; Jan-2005; W. Nelson | Mattio & Payri 2009; 2010 | FJ170418 | FJ170451 | FJ170388 |
| *Sargassum schnetteri* | LAF04287a | Colombia: Neguanje (TNNP), Magdalena; May-2009; O. Camacho | Camacho et al., 2015 | KF437932 | KF437950 | KF437963 |
| *Sargassum schnetteri* | LAF06612 | Colombia: Neguanje (TNNP), Magdalena; May-2009; O. Camacho | Camacho et al., 2015 | KF437931 | KF437949 | KF437962 |
| *Sargassum serratifolium* | CNUK PF1439 | South Korea: Seopseom, Jeju; Mar-2010; n.d. | Cho et al., 2012 | - | JF931842 | - |
| *Sargassum serratifolium* | n.d. | South Korea: Seongsan, Jeju; n.d. | Lee et al., 2022 | NC_066463 | - | NC_066459 |
| *Sargassum siliquastrum* | MBMD01281 | China: Zhangzi Is.; Jun-2014; n.d. | Huang et al., 2017 | KY411114 | KY411088 | KY432493 |
| *Sargassum sinicola* | BCE22 | n.d. | Andrade-Sorcia et al., 2014 | - | JX560124 | - |
| *Sargassum* sp. 1 | IRD5181 | Spain: Corralejo, Fuerteventura, Canary Is.; 2010; F. Mineur | Mattio & Payri, 2010 | HQ416139 | HQ416069 | HQ416016 |
| *Sargassum* sp. 1 | IRD5182 | Spain: El Cotillo, Fuerteventura, Canary Is.; 2010; F. Mineur | Mattio & Payri, 2010 | HQ416140 | HQ416070 | HQ416017 |
| *Sargassum spinuligerum* | IRD3978 | New Caledonia: Ile des Pins; Dec-2005; C. Payri | Mattio & Payri, 2009 | FJ170425 | FJ170460 | FJ170397 |
| *Sargassum spinuligerum* | IRD# TZ0400 | Tanzania; n.d.; H. Verbruggen | Mattio & Payri, 2010 | HQ416131 | HQ416072 | HQ416019 |
| *Sargassum spinuligerum* var. *crispata* | IRD3963 | New Caledonia: M'Bo Is.; Jul-2005; L. Mattio | Mattio & Payri, 2009 | FJ170429 | FJ170463 | FJ170402 |
| *Sargassum stenophyllum* | LAF06611 | Colombia: Neguanje (TNNP), Magdalena; Apr-2009; O. Camacho | Camacho et al., 2015 | KF437925 | KF437941 | KF437958 |
| *Sargassum swartzii* | IRD1532 | New Caledonia: Thio; Oct-2006; C. Berthault | Mattio et al., 2008; 2010; 2013 | KF413706 | EU882254 | EU100807 |
| *Sargassum thunbergii* | IRD5241 | Japan: Mitohama, Misaki; Oct-2010; F. Mineur | Dixon et al., 2014 | KF281980 | KF281927 | KF281785 |
| *Sargassum turbinarioides* | IRD1604 | New Caledonia: Ile des Pins; Dec-2005; C. Payri | Mattio et al., 2010 | EU882245 | EU882256 | EU882265 |
| *Sargassum ulixiei* | PF1472 | Mexico: Puerto Peñasco; May-2010; n.d. | Andrade-Sorcia et al., 2014 | - | JX560128 | - |
| *Sargassum vachellianum* | n.d. | China: Gouqi Island, Zhejiang; Apr-2014; n.d. | Bi & Zhou, 2016; Bi et al., 2017 | NC_027508 | - | KT188823 |
| *Sargassum vachellianum* | ITS-N | China: Nanji Is., Zhejiang; n.d.; n.d. | Bi et al., 2014 | - | KJ856000 | - |
| **Table S1** (continued) |  |  |  |  |  |  |
| *Sargassum vulgare* | SZNPattiS LA2 | Italy; 2012; n.d | Chiarore et al., 2012 | KJ572492 | KJ572484 | - |
| *Sargassum vulgare* | SZNPattiS Ca1 | Italy; 2012; n.d | Chiarore et al., 2012 | KJ572493 | KJ572479 | - |
| *Sargassum xochitliae* | UAMIZ1055 | Mexico: Is. Mujeres, Quintana Roo; Mar-2012; D. González Nieto | González-Nieto et al., 2020 | - | MT465425 | MT470854 |
| *Sargassum xochitliae* | UAMIZ1056 | Mexico: Is. Mujeres, Quintana Roo; Mar-2012; D. González Nieto | González-Nieto et al., 2020 | - | MT465424 | MT470853 |
| *Sargassopsis decurrens* | IRD1526 | New Caledonia: Konduyo Is.; Apr-2004; L. Mattio | Mattio et al., 2008 | EU100822 | EU100773 | EU100803 |
| *Turbinaria gracilis* | PERTH08435855 | Australia; 20-Oct-2009; R.R.M. Dixon | Dixon et al., 2014 | KF281803 | KF281840 | KF281999 |
| *Turbinaria ornata* | IRD5193 | Reunion Is.: St. Giles; Mar-2010; M. Zubia | Mattio et al., 2015 | KP720492 | KP720528 | KP720558 |
| *Turbinaria ornata* | D1599 | South Africa: Bhanga Nek, KwaZulu-Natal; Oct-2013; L. Mattio, R. Anderson, J.J. Bolton | Mattio et al., 2015 | KP720493 | KP720529 | KP720559 |
|  |  |  |  |  |  |  |

**References**

Andrade-Sorcia, G., Riosmena-Rodriguez, R., Muñiz-Salazar, R., López-Vivas, J. M., Boo, G. H., Lee, K. M., & Boo, S. M. (2014). Morphological reassessment and molecular assessment of *Sargassum* (Fucales: Phaeophyceae) species from the Gulf of California, Mexico. *Phytotaxa*, *183*(4), 201–223. <https://doi.org/10.11646/phytotaxa.183.4.1>

Bi, Y., Li, J., & Zhou, Z. (2017). Complete sequence of chloroplast genome from *Sargassum vachellianum* (Sargassaceae, Phaeophyceae): Genome structure and comparative analysis. *Aquaculture and Fisheries*, *2*(4), 157–164. <https://doi.org/10.1016/j.aaf.2017.06.006>

Bi, Y., Yang, X., & Zhou, Z. (2014). Characterization and phylogenetic analysis of ITS sequences in three geographic populations of *Sargassum vachellianum* (Fucales, Phaeophyceae). *Journal of Fisheries of China*, *38*(9), 1335–1344. <https://doi.org/10.3724/SP.J.1231.2014.49318>

Bi, Y., & Zhou, Z. (2016). Complete mitochondrial genome of the brown alga *Sargassum vachellianum* (Sargassaceae, Phaeophyceae). *Mitochondrial DNA Part A*, *27*(4), 2796–2797. <https://doi.org/10.3109/19401736.2015.1053071>

Camacho, O., Mattio, L., Draisma, S., Fredericq, S., & Diaz-Pulido, G. (2015). Morphological and molecular assessment of *Sargassum* (Fucales, Phaeophyceae) from Caribbean Colombia, including the proposal of *Sargassum giganteum* sp. nov., *Sargassum schnetteri* comb. nov. and *Sargassum* section *Cladophyllum* sect. nov. *Systematics and Biodiversity*, *13*(2), 105–130. <https://doi.org/10.1080/14772000.2014.972478>

Chiarore, A., Patti, F. P., & Buia, M. C. (2012). Variabilità morfologica e genetica di *Sargassum vulgare*. Studio pilota di una popolazione nell’area acidificata del “Castello Aragonese” (Ischia, Napoli). *Biologia Marina Mediterranea*, *19*(1), 57–58.

Cho, S. M., Lee, S. M., Ko, Y. D., Mattio, L., & Boo, S. M. (2012). Molecular systematic reassessment of *Sargassum* (Fucales, Phaeophyceae) in Korea using four gene regions. *Botanica Marina*, *55*(5), 473–484. <https://doi.org/10.1515/bot-2012-0109>

Dixon, R. R. M., Mattio, L., Huisman, J. M., Payri, C. E., Bolton, J. J., & Gurgel, C. F. D. (2014). North meets south – Taxonomic and biogeographic implications of a phylogenetic assessment of *Sargassum* subgenera *Arthrophycus* and *Bactrophycus* (Fucales, Phaeophyceae). *Phycologia*, *53*(1), 15–22. <https://doi.org/10.2216/13-173.1>

González-Nieto, D., Oliveira, M. C., Resendiz, M. L. N., Dreckmann, K. M., Mateo-Cid, L. E., & Senties, A. (2020). Molecular assessment of the genus *Sargassum* (Fucales, Phaeophyceae) from the Mexican coasts of the Gulf of Mexico and Caribbean, with the description of *S. xochitlae* sp. nov. *Phytotaxa*, *461*(4), 254–274. <https://doi.org/10.11646/phytotaxa.461.4.3>

Huang, C., Sun, Z., Gao, D., Yao, J., Hu, Z., Li, Y., Wang, Y., Xu, K., & Chen, W. (2017). Molecular analysis of *Sargassum* from the northern China seas. *Phytotaxa*, *319*(1), 71–83. <https://doi.org/10.11646/phytotaxa.319.1.3>

Lee, Y. J., Kim, Y. D., Uh, Y. R., Kim, Y. M., Seo, T.-H., Choi, S.-J., & Jang, C. S. (2022). Complete organellar genomes of six *Sargassum* species and development of species-specific markers. *Scientific Reports*, *12*(1), Article 1. <https://doi.org/10.1038/s41598-022-25443-4>

Mattio, L., Anderson, R. J., & Bolton, J. J. (2015). A revision of the genus *Sargassum* (Fucales, Phaeophyceae) in South Africa. *South African Journal of Botany*, *98*, 95–107. <https://doi.org/10.1016/j.sajb.2015.02.008>

Mattio, L., & Payri, C. E. (2009). Taxonomic revision of *Sargassum* species (Fucales, Phaeophyceae) from New Caledonia based on morphological and molecular analyses. *Journal of Phycology*, *45*(6), 1374–1388. <https://doi.org/10.1111/j.1529-8817.2009.00760.x>

Mattio, L., & Payri, C. E. (2010). Assessment of five markers as potential barcodes for identifying *Sargassum* subgenus *Sargassum* species (Phaeophyceae, Fucales). *Cryptogamie, Algologie*, *31*(4), 467–485.

Mattio, L., Payri, C. E., & Stiger-Pouvreau, V. (2008). Taxonomic revision of *Sargassum* (Fucales, Phaeophyceae) from French Polynesia based on morphological and molecular analyses. *Journal of Phycology*, *44*(6), 1541–1555. <https://doi.org/10.1111/j.1529-8817.2008.00597.x>

Mattio, L., Payri, C. E., & Verlaque, M. (2009). Taxonomic revision and geographic distribution of the subgenus *Sargassum* (Fucales, Phaeophyceae) in the western and central Pacific Islands based on morphological and molecular analyses. *Journal of Phycology*, *45*(5), 1213–1227. <https://doi.org/10.1111/j.1529-8817.2009.00737.x>

Mattio, L., Payri, C. E., Verlaque, M., & de Reviers, B. (2010). Taxonomic revision of *Sargassum* sect. *Acanthocarpicae* (Fucales, Phaeophyceae). *TAXON*, *59*(3), 896–904. <https://doi.org/10.1002/tax.593017>

Mattio, L., Zubia, M., Loveday, B., Crochelet, E., Duong, N., Payri, C. E., Bhagooli, R., & Bolton, J. J. (2013). *Sargassum* (Fucales, Phaeophyceae) in Mauritius and Réunion, western Indian Ocean: Taxonomic revision and biogeography using hydrodynamic dispersal models. *Phycologia*, *52*(6), 578–594. <https://doi.org/10.2216/13-150.1>

Phillips, N. E., Smith, C. M., & Morden, C. W. (2005). Testing systematic concepts of *Sargassum* (Fucales, Phaeophyceae) using portions of the *rbc*LS operon. *Phycological Research*, *53*(1), 1–10. <https://doi.org/10.1111/j.1440-183.2005.00368.x>
