# Supplementary material for: Multigene phylogenetics of Sargassum (Phaeophyceae) revealed low molecular diversity in contrast to high morphological variability in the NE Atlantic Ocean
Source: J Phycol. 2024 Oct 26;60(6):1528–56. doi: 10.1111/jpy.13517 (PMC11670286; doi:10.1111/jpy.13517)
Supplement: Supplementary file 5 — Table S2. Taxa included in the molecular analyses of the mitochondrial spacer (mtsp), with the collecting data, references and GenBank Accession No. n.d.: no data available. [file JPY-60-1528-s006.docx]

| **Table S2.** Taxa included in the molecular analyses of the mitochondrial spacer (*mtsp*), with the collecting data, references and GenBank Accession No. n.d.: no data available. | | | | |
| --- | --- | --- | --- | --- |
| **Organism** | **Specimen ID/Voucher** | **Collection site; Collection Date; Collector** | **References** | **Genbank Accn. No.** |
| *Sargassum cymosum* | SGU7/  TFCPhyc16455 | Spain: Altagay, Punta Hidalgo, Tenerife, Canary Islands; 30-Jan-2022; D. Alvarez-Canali | This study | **OR786554** |
| *Sargassum cymosum* | SGU8/  TFCPhyc16456 | Spain: Punta Brava, Tenerife, Canary Islands; 03-Feb-2022; D. Alvarez-Canali | This study | **OR786555** |
| *Sargassum desfontainesii* | SGU1/  TFCPhyc16449 | Spain: La Laja, El Hierro, Canary Islands; 09-Feb-2020; D. Alvarez-Canali | This study | **OR786548** |
| *Sargassum desfontainesii* | SGU2/  TFCPhyc16450 | Spain: Altagay, Punta Hidalgo, Tenerife, Canary Islands; 30-Jan-2022; D. Alvarez-Canali | This study | **OR786549** |
| *Sargassum desfontainesii* | SGU26/  TFCPhyc16474 | Spain: La Salemera, La Palma, Canary Islands; 18-Feb-2022; D. Alvarez-Canali | This study | **OR786573** |
| *Sargassum desfontainesii* | SGU28/  TFCPhyc16476 | Spain: Órzola, Lanzarote, Canary Islands; 02-Apr-2022; D. Alvarez-Canali | This study | **OR786575** |
| *Sargassum filipendula* | SGU9/  TFCPhyc16457 | Spain: Altagay, Punta Hidalgo, Tenerife, Canary Islands; 30-Jan-2022; D. Alvarez-Canali | This study | **OR786556** |
| *Sargassum filipendula* | SGU10/  TFCPhyc16458 | Spain: Punta Hidalgo, Tenerife, Canary Islands; 29-Oct-2019; D. Alvarez-Canali | This study | **OR786557** |
| *Sargassum filipendula* | SGU53/  TFCPhyc16484 | Spain: Montaña Amarilla, La Graciosa, Lanzarote, Canary Islands; 14-Nov-2019; D. Alvarez-Canali | This study | **OR786582** |
| *Sargassum flavifolium* | SGU13/  TFCPhyc16461 | Spain: Boca Cangrejo, Tenerife, Canary Islands; 25-Jan-2022; D. Alvarez-Canali | This study | **OR786560** |
| *Sargassum flavifolium* | SGU14/  TFCPhyc16462 | Spain: La Salemera, La Palma, Canary Islands; 23-Jun-2021; D. Alvarez-Canali | This study | **OR786561** |
| *Sargassum furcatum* | SGU19/  TFCPhyc16467 | Spain: Playa Chica, Lanzarote, Canary Islands; 30-Mar-2022; D. Alvarez-Canali | This study | **OR786566** |
| *Sargassum furcatum* | SGU20/  TFCPhyc16468 | Spain: Playa Nogales, La Palma, Canary Islands; 22-Jun-2021; D. Alvarez-Canali | This study | **OR786567** |
| *Sargassum furcatum* | SGU25/  TFCPhyc16473 | Spain: La Barranquera, Tenerife, Canary Islands; 31-Jan-2022; D. Alvarez-Canali | This study | **OR786572** |
| *Sargassum furcatum* | SGU40/  TFCPhyc16480 | Spain: Punta Fariones, Lanzarote, Canary Islands; 20-Jul-2020; D. Alvarez-Canali | This study | **OR786579** |
| *Sargassum orotavicum* | SGU5/  TFCPhyc16453 | Spain: Punta Brava, Tenerife, Canary Islands; 07-Oct-2021; D. Alvarez-Canali | This study | **OR786552** |
| *Sargassum orotavicum* | SGU6/  TFCPhyc16454 | Spain: Punta Brava, Tenerife, Canary Islands; 03-Feb-2022; D. Alvarez-Canali | This study | **OR786553** |
| *Sargassum ramifolium* | SGU3/  TFCPhyc16451 | Spain: La Laja, El Hierro, Canary Islands; 29-Feb-2020; D. Alvarez-Canali | This study | **OR786550** |
| *Sargassum ramifolium* | SGU4/  TFCPhyc16452 | Spain: Altagay, Punta Hidalgo, Tenerife, Canary Islands; 30-Jan-2022; D. Alvarez-Canali | This study | **OR786551** |
| **Table S2** (continued) |  |  |  |  |
| *Sargassum ramifolium* | SGU44/  TFCPhyc16481 | Spain: Punta Fariones, Lanzarote, Canary Islands; 20-Jul-2020; D. Alvarez-Canali | This study | **OR786580** |
| *Sargassum* sp. CI1 | SGU15/  TFCPhyc16463 | Spain: Playa Chica, Lanzarote, Canary Islands; 30-Mar-2022; D. Alvarez-Canali | This study | **OR786562** |
| *Sargassum* sp. CI1 | SGU16/  TFCPhyc16464 | Spain: La Barranquera, Tenerife, Canary Islands; 31-Jan-2022; D. Alvarez-Canali | This study | **OR786563** |
| *Sargassum* sp. CI1 | SGU17/  TFCPhyc16465 | Spain: Punta Hidalgo, Tenerife, Canary Islands; 24-Jan-2022; D. Alvarez-Canali | This study | **OR786564** |
| *Sargassum* sp. CI1 | SGU18/  TFCPhyc16466 | Spain: La Caleta (Güimar), Tenerife, Canary Islands; 09-Feb-2021; D. Alvarez-Canali | This study | **OR786565** |
| *Sargassum* sp. CI1 | SGU27/  TFCPhyc16475 | Spain: La Salemera, La Palma, Canary Islands; 18-Feb-2022; D. Alvarez-Canali | This study | **OR786574** |
| *Sargassum* sp. CI1 | SGU30/  TFCPhyc16478 | Spain: Punta Hidalgo, Tenerife, Canary Islands; 13-Oct-2019; D. Alvarez-Canali | This study | **OR786577** |
| *Sargassum* sp. CI2 | SGU23/  TFCPhyc16471 | Spain: Cuevas Coloradas, Montaña Clara, Lanzarote, Canary Islands; 22-Sep-2020; D. Alvarez-Canali | This study | **OR786570** |
| *Sargassum* sp. CI2 | SGU50/  TFCPhyc16483 | Spain: Montaña Amarilla, La Graciosa, Lanzarote, Canary Islands; 14-Nov-2019; D. Alvarez-Canali | This study | **OR786581** |
| *Sargassum* sp. CI3 | SGU21/  TFCPhyc16469 | Spain: Cuevas Coloradas, Montaña Clara, Lanzarote, Canary Islands; 24-Mar-2021; D. Alvarez-Canali | This study | **OR786568** |
| *Sargassum* sp. CI3 | SGU22/  TFCPhyc16470 | Spain: Alegranza, Lanzarote, Canary Islands; 22-Sep-2020; D. Alvarez-Canali | This study | **OR786569** |
| *Sargassum* sp. CI3 | SGU24/  TFCPhyc16472 | Spain: Cuevas Coloradas, Montaña Clara, Lanzarote, Canary Islands; 24-Mar-2021; D. Alvarez-Canali | This study | **OR786571** |
| *Sargassum* sp. CI3 | SGU29/  TFCPhyc16477 | Spain: Órzola, Lanzarote, Canary Islands; 02-Apr-2022; D. Alvarez-Canali | This study | **OR786576** |
| *Sargassum* sp. CI3 | SGU38/  TFCPhyc16479 | Spain: Punta Fariones, Lanzarote, Canary Islands; 20-Jul-2020; D. Alvarez-Canali | This study | **OR786578** |
| *Sargassum stenophyllum* | SGU11/  TFCPhyc16459 | Spain: Punta Brava, Tenerife, Canary Islands; 03-Feb-2022; D. Alvarez-Canali | This study | **OR786558** |
| *Sargassum stenophyllum* | SGU12/  TFCPhyc16460 | Spain: La Barranquera, Tenerife, Canary Islands; 31-Jan-2022; D. Alvarez-Canali | This study | **OR786559** |
| *Sargassum aquifolium* | n.d. | China: Dadonghai Bay, Sanya, Hainan Province; 1-Apr-2014; n.d. | Liu et al., 2017 | KT266809 |
| *Sargassum carpophyllum* | IRD1511 | New Caledonia: Feycinet Is.; Jul-2005; L. Mattio | Mattio & Payri, 2010 | HQ416091 |
| *Sargassum confusum* | n.d. | South Korea: Chujado Is., Jeju; n.d.; n.d. | Lee et al., 2022 | NC_066460 |
| *Sargassum desfontainesii* | L 0609351 | Canary Islands: Punta del Hidalgo, Tenerife; 25-Jan-2007; W.F. Prud'homme van Reine | Draisma et al., 2010 | FM993075 |
| *Sargassum elegans* | L SGAD1303 | South Africa: Palm Beach, KwaZulu Natal; 22-Aug-2005; S. Draisma | Draisma et al., 2010 | FM993074 |
| *Sargassum feldmannii* | n.d. | n.d. | Unpublished | NC_063979 |
| *Sargassum fluitans* III | C256-039-NT_38 | Atlantic Ocean: 14.9502778 -49.468611; 4-Dec-2014; n.d. | Amaral-Zettler et al., 2016 | NC_033385 |
| **Table S2** (continued) |  |  |  |  |
| *Sargassum fulvellum* | n.d. | South Korea: Jeopdo Is., Jeollanamdo; n.d.; n.d. | Lee et al., 2022 | NC_066461 |
| *Sargassum fusiforme* | n.d. | China: Nanji Is., Zhejiang; n.d.; n.d. | Liu, Pang & Luo 2016 | NC_024655 |
| *Sargassum graminifolium* | n.d. | n.d. | Unpublished | NC_063976 |
| *Sargassum hemiphyllum* | n.d. | China: Shen'ao Bay, Guangdong; n.d.; n.d. | Liu, Pang & Chen 2016 | NC_024861 |
| *Sargassum henslowianum* | n.d. | n.d. | Unpublished | NC_063981 |
| *Sargassum horneri* | n.d. | Japan: Hiranai, Aomori, Tohoku; n.d.; n.d. | Unpublished | MG774890 |
| *Sargassum howeanum* | IRD3962 | New Caledonia: Ouano; Jun-2005; L. Mattio | Mattio & Payri, 2010 | HQ416093 |
| *Sargassum ilicifolium* | n.d. | China: Dadonghai Bay, Sanya, Hainan Province; 1-Apr-2014.; n.d. | Liu et al., 2017 | KT272403 |
| *Sargassum kjellmanianum* | FIO2020059703 | China: Lidao Bay, Shandong Province; n.d.; n.d. | Xu et al., 2022 | NC_063522 |
| *Sargassum macrocarpum* | n.d. | South Korea: Geumodo Is., Jeollanamdo; n.d.; n.d. | Lee et al., 2022 | NC_066462 |
| *Sargassum mcclurei* | n.d. | n.d. | Unpublished | NC_063980 |
| *Sargassum muticum* | n.d. | China: Sanggou Bay; n.d.; n.d. | Liu & Pang 2016a | NC_024614 |
| *Sargassum natans* I | C241-025-NT_1 | Atlantic Ocean: 31.65 -64.261667; 27-May-2012; n.d. | Amaral-Zettler et al., 2016 | NC_033384 |
| *Sargassum natans* VIII | C256-039-NT_32 | Atlantic Ocean: 14.9502778 -49.468611; 4-Dec-2014; n.d. | Amaral-Zettler et al., 2016 | KY084908 |
| *Sargassum nigrifolium* | n.d. | n.d. | Unpublished | NC_036707 |
| *Sargassum obtusifolium* | UPF2651 | French Polynesia: Rapa, Australs Is.; Nov-2002; C. Payri | Mattio & Payri, 2010 | HQ416105 |
| *Sargassum pacificum* | UPF3972 | French Polynesia: Raiatea, Society Is.; Mar-2003; C. Payri, V. Stiger-Pouvreau | Mattio & Payri, 2010 | HQ416111 |
| *Sargassum patens* | MBM286786 | China: Bailong Is., Guangxi Province; 20-Jan-2020; n.d. | Li & Bi 2020 | NC_052831 |
| *Sargassum phyllocystum* | n.d. | n.d. | Unpublished | NC_063978 |
| *Sargassum plagiophyllum* | M03-2 | Malaysia: Penang; 1-Oct-2019; S. Draisma | Zhang et al., 2022 | NC_064731 |
| *Sargassum polycystum* | IRD# TZ0049 | Tanzania; n.d.; H. Verbruggen | Mattio & Payri 2010 | HQ416112 |
| *Sargassum polycystum* | IRD1590 | Fiji: Kiuva Reef; May-2007; L. Mattio | Mattio & Payri 2010 | HQ416113 |
| *Sargassum polycystum* | G6-10 | Thailand: Trat; 7-Jan-2019; S. Draisma | Zhang et al., 2022 | NC_064729 |
| *Sargassum scabridum* | WELT A28417 | New Zealand: Auckland; Jan-2005; W. Nelson | Mattio & Payri, 2010 | HQ416114 |
| *Sargassum scabridum* | WELT A28412 | New Zealand: Meyer Is., Kermadec; 2005; C. Duffy | Mattio & Payri, 2010 | HQ416115 |
| *Sargassum serratifolium* | n.d. | South Korea: Seongsan, Jeju; n.d.; n.d. | Lee et al., 2022 | NC_066463 |
| *Sargassum siliquastrum* | MBM286789 | China: Nanghuangcheng Is.; 13-Jan-2020 | Li et al., 2020 | NC_050651 |
| *Sargassum sinclairii* | L SGAD1076 | New Zealand: Princess Bay, Wellington; Jan-2006; C. Bödeker | Draisma et al., 2010 | FM993056 |
| *Sargassum* sp. | L SGAD1005 | Australia; 05-Dec-2004; L.McIvor | Draisma et al., 2010 | FM993055 |
| *Sargassum* sp. | L SGAD0509434 | Indonesia: Thousand Is., Pulau Air; 17-Sep-2005; S. Draisma & W.F. Prud'homme van Reine | Draisma et al., 2010 | FM993073 |
| *Sargassum* sp. 1 | IRD5181 | Spain: Corralejo, Fuerteventura, Canary Is.; 2010; F. Mineur | Mattio & Payri, 2010 | HQ416116 |
| *Sargassum* sp. 1 | IRD5182 | Spain: El Cotillo, Fuerteventura, Canary Is.; 2010; F. Mineur | Mattio & Payri, 2010 | HQ416117 |
| *Sargassum* sp. 2 | IRD1634 | Vanuatu: Port Vila; 2006; C. Payri | Mattio & Payri 2010 | HQ416118 |
| *Sargassum* sp. 2 | IRD1609 | New Caledonia: Rocher á la voile; 2005; L. Mattio | Mattio & Payri 2010 | HQ416119 |
| *Sargassum spinuligerum* | IRD# TZ0400 | Tanzania; n.d.; H. Verbruggen | Mattio & Payri, 2010 | HQ416124 |
| *Sargassum spinuligerum* | n.d. | China: Dadonghai Bay, Sanya, Hainan Province; 1-Apr-2014.; n.d. | Liu et al., 2017 | NC_034272 |
| *Sargassum thunbergii* | n.d. | China: Sanggou Bay; n.d.; n.d. | Liu & Pang 2016b | NC_026700 |
| **Table S2** (continued) |  |  |  |  |
| *Sargassum vachellianum* | n.d. | China: Gouqi Island, Zhejiang; Apr-2014; n.d. | Bi & Zhou 2016 | NC_027508 |
| *Sargassum yezoense* | AL00070893 | South Korea: Uljin, Gyeongsangbuk-do; 5-Apr-2017; n.d. | Kim et al., 2018 | NC_038156 |
| *Sargassopsis decurrens* | SAP073149 | New Caledonia: Larégnère Is.; May-1999; C. Payri | Draisma et al., 2010 | FM993077 |
| *Turbinaria ornata* | n.d. | China: Dadonghai Bay, Sanya, Hainan Province; 1-Apr-2014.; n.d. | Liu & Pang 2015 | NC_0274113 |
|  |  |  |  |  |

**References**

Amaral-Zettler, L. A., Dragone, N. B., Schell, J., Slikas, B., Murphy, L. G., Morrall, C. E., & Zettler, E. R. (2017). Comparative mitochondrial and chloroplast genomics of a genetically distinct form of *Sargassum* contributing to recent “Golden Tides” in the Western Atlantic. *Ecology and Evolution*, *7*(2), 516–525. <https://doi.org/10.1002/ece3.2630>

Bi, Y., & Zhou, Z. (2016). Complete mitochondrial genome of the brown alga *Sargassum vachellianum* (Sargassaceae, Phaeophyceae). *Mitochondrial DNA Part A*, *27*(4), 2796–2797. <https://doi.org/10.3109/19401736.2015.1053071>

Draisma, S. G. A., Ballesteros, E., Rousseau, F., & Thibaut, T. (2010). DNA sequence data demonstrate the polyphyly of the genus *Cystoseira* and other Sargassaceae genera (Phaeophyceae). *Journal of Phycology*, *46*(6), 1329–1345. <https://doi.org/10.1111/j.1529-8817.2010.00891.x>

Kim, K. M., Choi, J. W., Yoon, H. S., Jang, H. S., & Hong, J. W. (2018). Complete mitochondrial genome of *Sargassum yezoense* (Sargassaceae, Phaeophyceae). *Mitochondrial DNA Part B*, *3*(1), 424–425. <https://doi.org/10.1080/23802359.2018.1457993>

Lee, Y. J., Kim, Y. D., Uh, Y. R., Kim, Y. M., Seo, T.-H., Choi, S.-J., & Jang, C. S. (2022). Complete organellar genomes of six *Sargassum* species and development of species-specific markers. *Scientific Reports*, *12*(1), 20981. <https://doi.org/10.1038/s41598-022-25443-4>

Li, J., & Bi, Y. (2020). Phylogenetic analysis of the complete mitochondrial genome of *Sargassum patens* C. Agardh (Phaeophyceae). *Mitochondrial DNA Part B*, *5*(4), 3827–3828. <https://doi.org/10.1080/23802359.2020.1841580>

Li, J., Li, H., & Bi, Y. (2020). The complete mitochondrial genome of *Sargassum siliquastrum* (Phaeophyceae) and its phylogenetic analysis. *Mitochondrial DNA Part B*, *5*(3), 3565–3566. <https://doi.org/10.1080/23802359.2020.1829134>

Liu, F., Li, X., & Che, Z. (2017). Mitochondrial genome sequences uncover evolutionary relationships of two *Sargassum* subgenera, *Bactrophycus* and *Sargassum*. *Journal of Applied Phycology*, *29*(6), 3261–3270. <https://doi.org/10.1007/s10811-017-1143-1>

Liu, F., & Pang, S. (2015). Mitochondrial genome of *Turbinaria ornata* (Sargassaceae, Phaeophyceae): Comparative mitogenomics of brown algae. *Current Genetics*, *61*(4), 621–631. <https://doi.org/10.1007/s00294-015-0488-8>

Liu, F., & Pang, S. (2016a). Complete mitochondrial genome of the invasive brown alga *Sargassum muticum* (Sargassaceae, Phaeophyceae). *Mitochondrial DNA Part A*, *27*(2), 1129–1130. <https://doi.org/10.3109/19401736.2014.933333>

Liu, F., & Pang, S. (2016b). Mitochondrial genome of *Sargassum thunbergii*: Conservation and variability of mitogenomes within the subgenus *Bactrophycus*. *Mitochondrial DNA Part A*, *27*(5), 3186–3188. <https://doi.org/10.3109/19401736.2015.1007328>

Liu, F., Pang, S., & Chen, W. (2016). Complete mitochondrial genome of the brown alga *Sargassum hemiphyllum* (Sargassaceae, Phaeophyceae): Comparative analyses. *Mitochondrial DNA Part A*, *27*(2), 1468–1470. <https://doi.org/10.3109/19401736.2014.953096>

Liu, F., Pang, S., & Luo, M. (2016). Complete mitochondrial genome of the brown alga *Sargassum fusiforme* (Sargassaceae, Phaeophyceae): Genome architecture and taxonomic consideration. *Mitochondrial DNA Part A*, *27*(2), 1158–1160. <https://doi.org/10.3109/19401736.2014.936417>

Mattio, L., & Payri, C. E. (2010). Assessment of five markers as potential barcodes for identifying *Sargassum* subgenus *Sargassum* species (Phaeophyceae, Fucales). *Cryptogamie, Algologie*, *31*(4), 467–485.

Xu, H., Wang, X., Zhang, L., He, Y., Zhang, Y., Qu, C., & Miao, J. (2022). The complete mitochondrial genome of *Sargassum kjellmanianum* (Sargassaceae) and phylogenetic analysis of *Sargassum kjellmanianum* (Sargassaceae). *Mitochondrial DNA Part B*, *7*(10), 1785–1786. <https://doi.org/10.1080/23802359.2022.2127335>

Zhang, S., Liang, Y., Zhang, J., Draisma, S. G. A., & Duan, D. (2022). Organellar genome comparisons of *Sargassum polycystum* and *S. plagiophyllum* (Fucales, Phaeophyceae) with other *Sargassum* species. *BMC Genomics*, *23*, 629. <https://doi.org/10.1186/s12864-022-08862-5>
