# Supplementary material for: Multigene phylogenetics of Sargassum (Phaeophyceae) revealed low molecular diversity in contrast to high morphological variability in the NE Atlantic Ocean
Source: J Phycol. 2024 Oct 26;60(6):1528–56. doi: 10.1111/jpy.13517 (PMC11670286; doi:10.1111/jpy.13517)
Supplement: Supplementary file 6 — Table S3. Taxa included in the molecular analyses of the protein‐coding mitochondrial genes (cox2, extended cox3, nad6), with collecting data, references and GenBank Accession No. n.d.: no data available. [file JPY-60-1528-s004.docx]

| **Table S3.** Taxa included in the molecular analyses of the protein-coding mitochondrial genes (*cox*2, extended *cox*3, *nad*6), with collecting data, references and GenBank Accession No. n.d.: no data available. | | | | | | |
| --- | --- | --- | --- | --- | --- | --- |
| **Organism** | **Specimen ID/Voucher** | **Collection site; Collection Date; Collector** | **References** | **GenBank Accession No.** | | |
|  |  |  |  | ***cox*2** | ***cox*3** | ***nad*6** |
| *Sargassum desfontainesii* | SGU1/  TFCPhyc16449 | Spain: La Laja, El Hierro, Canary Islands; 09-Feb-2020; D. Alvarez-Canali | This study | **OR786536** | **OR786511** | **OR786583** |
| *Sargassum desfontainesii* | SGU2/  TFCPhyc16450 | Spain: Altagay, Punta Hidalgo, Tenerife, Canary Islands; 30-Jan-2022; D. Alvarez-Canali | This study | **OR786537** | **OR786512** | **OR786584** |
| *Sargassum filipendula* | SGU9/  TFCPhyc16457 | Spain: Altagay, Punta Hidalgo, Tenerife, Canary Islands; 30-Jan-2022; D. Alvarez-Canali | This study | **OR786542** | **OR786519** | **OR786589** |
| *Sargassum filipendula* | SGU10/  TFCPhyc16458 | Spain: Punta Hidalgo, Tenerife, Canary Islands; 29-Oct-2019; D. Alvarez-Canali | This study | **OR786543** | **OR786520** | **OR786590** |
| *Sargassum furcatum* | SGU19/  TFCPhyc16467 | Spain: Playa Chica, Lanzarote, Canary Islands; 30-Mar-2022; D. Alvarez-Canali | This study | **OR786546** | **OR786528** | **OR786593** |
| *Sargassum furcatum* | SGU20/  TFCPhyc16468 | Spain: Playa Nogales, La Palma, Canary Islands; 22-Jun-2021; D. Alvarez-Canali | This study | **OR786547** | **OR786529** | **OR786594** |
| *Sargassum orotavicum* | SGU5/  TFCPhyc16453 | Spain: Punta Brava, Tenerife, Canary Islands; 07-Oct-2021; D. Alvarez-Canali | This study | **OR786540** | **OR786515** | **OR786587** |
| *Sargassum orotavicum* | SGU6/  TFCPhyc16454 | Spain: Punta Brava, Tenerife, Canary Islands; 03-Feb-2022; D. Alvarez-Canali | This study | **OR786541** | **OR786516** | **OR786588** |
| *Sargassum ramifolium* | SGU3/  TFCPhyc16451 | Spain: La Laja, El Hierro, Canary Islands; 29-Feb-2020; D. Alvarez-Canali | This study | **OR786538** | **OR786513** | **OR786585** |
| *Sargassum ramifolium* | SGU4/  TFCPhyc16452 | Spain: Altagay, Punta Hidalgo, Tenerife, Canary Islands; 30-Jan-2022; D. Alvarez-Canali | This study | **OR786539** | **OR786514** | **OR786586** |
| *Sargassum* sp. CI1 | SGU15/  TFCPhyc16463 | Spain: Playa Chica, Lanzarote, Canary Islands; 30-Mar-2022; D. Alvarez-Canali | This study | **OR786544** | **OR786525** | **OR786591** |
| *Sargassum* sp. CI1 | SGU16/  TFCPhyc16464 | Spain: La Barranquera, Tenerife, Canary Islands; 31-Jan-2022; D. Alvarez-Canali | This study | **OR786545** | **OR786526** | **OR786592** |
| *Sargassum aquifolium* | n.d. | China: Dadonghai Bay, Sanya, Hainan Province; 1-Apr-2014.; n.d. | Liu et al., 2017 | KT266809 | KT266809 | KT266809 |
| *Sargassum confusum* | n.d. | South Korea: Chujado Is., Jeju; n.d.; n.d. | Lee et al., 2022 | NC_066460 | NC_066460 | NC_066460 |
| *Sargassum feldmannii* | n.d. | n.d. | Unpublished | NC_063979 | NC_063979 | NC_063979 |
| *Sargassum fluitans* III | C256-039-NT_38 | Atlantic Ocean: 14.9502778 -49.468611; 4-Dec-2014; n.d. | Amaral-Zettler et al., 2016 | NC_033385 | NC_033385 | NC_033385 |
| *Sargassum fulvellum* | n.d. | South Korea: Jeopdo Is., Jeollanamdo; n.d.; n.d. | Lee et al., 2022 | NC_066461 | NC_066461 | NC_066461 |
| *Sargassum fusiforme* | n.d. | China: Nanji Is., Zhejiang; n.d.; n.d. | Liu, Pang & Luo 2016 | NC_024655 | NC_024655 | NC_024655 |
| *Sargassum graminifolium* | n.d. | n.d. | Unpublished | NC_063976 | NC_063976 | NC_063976 |
| *Sargassum hemiphyllum* | n.d. | China: Shen'ao Bay, Guangdong; n.d.; n.d. | Liu, Pang & Chen 2016 | NC_024861 | NC_024861 | NC_024861 |
| *Sargassum henslowianum* | n.d. | n.d. | Unpublished | NC_063981 | NC_063981 | NC_063981 |
| *Sargassum horneri* | n.d. | Japan: Hiranai, Aomori, Tohoku; n.d.; n.d. | Unpublished | MG774890 | MG774890 | MG774890 |
| *Sargassum ilicifolium* | n.d. | China: Dadonghai Bay, Sanya, Hainan Province; 1-Apr-2014.; n.d. | Liu et al., 2017 | KT272403 | KT272403 | KT272403 |
| **Table S3** (continued) |  |  |  |  |  |  |
| *Sargassum kjellmanianum* | FIO2020059703 | China: Lidao Bay, Shandong Province; n.d.; n.d. | Xu et al., 2022 | NC_063522 | NC_063522 | NC_063522 |
| *Sargassum macrocarpum* | n.d. | South Korea: Geumodo Is., Jeollanamdo; n.d.; n.d. | Lee et al., 2022 | NC_066462 | NC_066462 | NC_066462 |
| *Sargassum mcclurei* | n.d. | n.d. | Unpublished | NC_063980 | NC_063980 | NC_063980 |
| *Sargassum muticum* | n.d. | China: Sanggou Bay; n.d.; n.d. | Liu & Pang 2016a | NC_024614 | NC_024614 | NC_024614 |
| *Sargassum natans* I | C241-025-NT_1 | Atlantic Ocean: 31.65 -64.261667; 27-May-2012; n.d. | Amaral-Zettler et al., 2016 | NC_033384 | NC_033384 | NC_033384 |
| *Sargassum natans* VIII | C256-039-NT_32 | Atlantic Ocean: 14.9502778 -49.468611; 4-Dec-2014; n.d. | Amaral-Zettler et al., 2016 | KY084908 | KY084908 | KY084908 |
| *Sargassum nigrifolium* | n.d. | n.d. | Unpublished | NC_036707 | NC_036707 | NC_036707 |
| *Sargassum patens* | MBM286786 | China: Bailong Is., Guangxi Province; 20-Jan-2020 | Li & Bi 2020 | NC_052831 | NC_052831 | NC_052831 |
| *Sargassum phyllocystum* | n.d. | n.d. | Unpublished | NC_063978 | NC_063978 | NC_063978 |
| *Sargassum plagiophyllum* | M03-2 | Malaysia: Penang; 1-Oct-2019; S. Draisma | Zhang et al., 2022 | NC_064731 | NC_064731 | NC_064731 |
| *Sargassum polycystum* | n.d. | China: Dadonghai Bay, Sanya, Hainan Province; 1-Apr-2014.; n.d. | Liu et al., 2017 | KT280278 | KT280278 | KT280278 |
| *Sargassum serratifolium* | n.d. | South Korea: Seongsan, Jeju; n.d.; n.d. | Lee et al., 2022 | NC_066463 | NC_066463 | NC_066463 |
| *Sargassum siliquastrum* | MBM286789 | China: Nanghuangcheng Is.; 13-Jan-2020; n.d. | Li et al., 2020 | NC_050651 | NC_050651 | NC_050651 |
| *Sargassum spinuligerum* | n.d. | China: Dadonghai Bay, Sanya, Hainan Province; 1-Apr-2014.; n.d. | Liu et al., 2017 | NC_034272 | NC_034272 | NC_034272 |
| *Sargassum thunbergii* | n.d. | China: Sanggou Bay; n.d.; n.d. | Liu & Pang 2016b | NC_026700 | NC_026700 | NC_026700 |
| *Sargassum vachellianum* | n.d. | China: Gouqi Island, Zhejiang; Apr-2014; n.d. | Bi & Zhou 2016 | NC_027508 | NC_027508 | NC_027508 |
| *Sargassum yezoense* | AL00070893 | South Korea: Uljin, Gyeongsangbuk-do; 5-Apr-2017; n.d. | Kim et al., 2018 | NC_038156 | NC_038156 | NC_038156 |
| *Turbinaria ornata* | n.d. | China: Dadonghai Bay, Sanya, Hainan Province; 1-Apr-2014.; n.d. | Liu & Pang 2015 | NC_027413 | NC_027413 | NC_027413 |
|  |  |  |  |  |  |  |

**References**

Amaral-Zettler, L. A., Dragone, N. B., Schell, J., Slikas, B., Murphy, L. G., Morrall, C. E., & Zettler, E. R. (2017). Comparative mitochondrial and chloroplast genomics of a genetically distinct form of *Sargassum* contributing to recent “Golden Tides” in the Western Atlantic. *Ecology and Evolution*, *7*(2), 516–525. <https://doi.org/10.1002/ece3.2630>

Bi, Y., & Zhou, Z. (2016). Complete mitochondrial genome of the brown alga *Sargassum vachellianum* (Sargassaceae, Phaeophyceae). *Mitochondrial DNA Part A*, *27*(4), 2796–2797. <https://doi.org/10.3109/19401736.2015.1053071>

Kim, K. M., Choi, J. W., Yoon, H. S., Jang, H. S., & Hong, J. W. (2018). Complete mitochondrial genome of *Sargassum yezoense* (Sargassaceae, Phaeophyceae). *Mitochondrial DNA Part B*, *3*(1), 424–425. <https://doi.org/10.1080/23802359.2018.1457993>

Lee, Y. J., Kim, Y. D., Uh, Y. R., Kim, Y. M., Seo, T.-H., Choi, S.-J., & Jang, C. S. (2022). Complete organellar genomes of six *Sargassum* species and development of species-specific markers. *Scientific Reports*, *12*(1), 20981. <https://doi.org/10.1038/s41598-022-25443-4>

Li, J., & Bi, Y. (2020). Phylogenetic analysis of the complete mitochondrial genome of *Sargassum patens* C. Agardh (Phaeophyceae). *Mitochondrial DNA Part B*, *5*(4), 3827–3828. <https://doi.org/10.1080/23802359.2020.1841580>

Li, J., Li, H., & Bi, Y. (2020). The complete mitochondrial genome of *Sargassum siliquastrum* (Phaeophyceae) and its phylogenetic analysis. *Mitochondrial DNA Part B*, *5*(3), 3565–3566. <https://doi.org/10.1080/23802359.2020.1829134>

Liu, F., Li, X., & Che, Z. (2017). Mitochondrial genome sequences uncover evolutionary relationships of two *Sargassum* subgenera, *Bactrophycus* and *Sargassum*. *Journal of Applied Phycology*, *29*(6), 3261–3270. <https://doi.org/10.1007/s10811-017-1143-1>

Liu, F., & Pang, S. (2015). Mitochondrial genome of *Turbinaria ornata* (Sargassaceae, Phaeophyceae): Comparative mitogenomics of brown algae. *Current Genetics*, *61*(4), 621–631. <https://doi.org/10.1007/s00294-015-0488-8>

Liu, F., & Pang, S. (2016a). Complete mitochondrial genome of the invasive brown alga *Sargassum muticum* (Sargassaceae, Phaeophyceae). *Mitochondrial DNA Part A*, *27*(2), 1129–1130. <https://doi.org/10.3109/19401736.2014.933333>

Liu, F., & Pang, S. (2016b). Mitochondrial genome of *Sargassum thunbergii*: Conservation and variability of mitogenomes within the subgenus *Bactrophycus*. *Mitochondrial DNA Part A*, *27*(5), 3186–3188. <https://doi.org/10.3109/19401736.2015.1007328>

Liu, F., Pang, S., & Chen, W. (2016). Complete mitochondrial genome of the brown alga *Sargassum hemiphyllum* (Sargassaceae, Phaeophyceae): Comparative analyses. *Mitochondrial DNA Part A*, *27*(2), 1468–1470. <https://doi.org/10.3109/19401736.2014.953096>

Liu, F., Pang, S., & Luo, M. (2016). Complete mitochondrial genome of the brown alga *Sargassum fusiforme* (Sargassaceae, Phaeophyceae): Genome architecture and taxonomic consideration. *Mitochondrial DNA Part A*, *27*(2), 1158–1160. <https://doi.org/10.3109/19401736.2014.936417>

Mattio, L., & Payri, C. E. (2010). Assessment of five markers as potential barcodes for identifying *Sargassum* subgenus *Sargassum* species (Phaeophyceae, Fucales). *Cryptogamie, Algologie*, *31*(4), 467–485.

Xu, H., Wang, X., Zhang, L., He, Y., Zhang, Y., Qu, C., & Miao, J. (2022). The complete mitochondrial genome of *Sargassum kjellmanianum* (Sargassaceae) and phylogenetic analysis of *Sargassum kjellmanianum* (Sargassaceae). *Mitochondrial DNA Part B*, *7*(10), 1785–1786. <https://doi.org/10.1080/23802359.2022.2127335>

Zhang, S., Liang, Y., Zhang, J., Draisma, S. G. A., & Duan, D. (2022). Organellar genome comparisons of *Sargassum polycystum* and *S. plagiophyllum* (Fucales, Phaeophyceae) with other Sargassum species. *BMC Genomics*, *23*, 629. <https://doi.org/10.1186/s12864-022-08862-5>
