# Supplementary material for: Multigene phylogenetics of Sargassum (Phaeophyceae) revealed low molecular diversity in contrast to high morphological variability in the NE Atlantic Ocean
Source: J Phycol. 2024 Oct 26;60(6):1528–56. doi: 10.1111/jpy.13517 (PMC11670286; doi:10.1111/jpy.13517)
Supplement: Supplementary file 7 — Table S4. Taxa included in the molecular analyses of the protein‐coding chloroplastic genes (atpB, clpC, psbC), with collecting data, references and GenBank Accession No. n.d.: no data available. [file JPY-60-1528-s001.docx]

| **Table S4.** Taxa included in the molecular analyses of the protein-coding chloroplastic genes (*atp*B, *clp*C, *psb*C), with collecting data, references and GenBank Accession No. n.d.: no data available. | | | | | | |
| --- | --- | --- | --- | --- | --- | --- |
| **Organism** | **Specimen ID/Voucher** | **Collection site; Collection Date; Collector** | **References** | **GenBank Accession No.** | | |
|  |  |  |  | ***atp*B** | ***clp*C** | ***psb*C** |
| *Sargassum cymosum* | SGU7/  TFCPhyc16455 | Spain: Altagay, Punta Hidalgo, Tenerife, Canary Islands; 30-Jan-2022; D. Alvarez-Canali | This study | **-** | **-** | **OR786596** |
| *Sargassum desfontainesii* | SGU1/  TFCPhyc16449 | Spain: La Laja, El Hierro, Canary Islands; 09-Feb-2020; D. Alvarez-Canali | This study | **OR786495** | **OR786503** | **-** |
| *Sargassum desfontainesii* | SGU2/  TFCPhyc16450 | Spain: Altagay, Punta Hidalgo, Tenerife, Canary Islands; 30-Jan-2022; D. Alvarez-Canali | This study | **OR786496** | **OR786504** | **OR786595** |
| *Sargassum desfontainesii* | SGU26/  TFCPhyc16474 | Spain: La Salemera, La Palma, Canary Islands; 18-Feb-2022; D. Alvarez-Canali | This study | **-** | **-** | **OR786607** |
| *Sargassum desfontainesii* | SGU28/  TFCPhyc16476 | Spain: Órzola, Lanzarote, Canary Islands; 02-Apr-2022; D. Alvarez-Canali | This study | **-** | **-** | **OR786608** |
| *Sargassum filipendula* | SGU9/  TFCPhyc16457 | Spain: Altagay, Punta Hidalgo, Tenerife, Canary Islands; 30-Jan-2022; D. Alvarez-Canali | This study | **OR786498** | **OR786507** | **OR786599** |
| *Sargassum flavifolium* | SGU13  /TFCPhyc16461 | Spain: Boca Cangrejo, Tenerife, Canary Islands; 25-Jan-2022; D. Alvarez-Canali | This study | **-** | **-** | **OR786602** |
| *Sargassum furcatum* | SGU19/  TFCPhyc16467 | Spain: Playa Chica, Lanzarote, Canary Islands; 30-Mar-2022; D. Alvarez-Canali | This study | **OR786501** | **OR786510** | **OR786605** |
| *Sargassum furcatum* | SGU20/  TFCPhyc16468 | Spain: Playa Nogales, La Palma, Canary Islands; 22-Jun-2021; D. Alvarez-Canali | This study | **OR786502** | **-** | **-** |
| *Sargassum orotavicum* | SGU5/  TFCPhyc16453 | Spain: Punta Brava, Tenerife, Canary Islands; 07-Oct-2021; D. Alvarez-Canali | This study | **-** | **OR786505** | **-** |
| *Sargassum orotavicum* | SGU6/  TFCPhyc16454 | Spain: Punta Brava, Tenerife, Canary Islands; 03-Feb-2022; D. Alvarez-Canali | This study | **OR786497** | **OR786506** | **OR786595** |
| *Sargassum ramifolium* | SGU3/  TFCPhyc16451 | Spain: La Laja, El Hierro, Canary Islands; 29-Feb-2020; D. Alvarez-Canali | This study | **-** | **-** | **OR786596** |
| *Sargassum* sp. CI1 | SGU15/  TFCPhyc16463 | Spain: Playa Chica, Lanzarote, Canary Islands; 30-Mar-2022; D. Alvarez-Canali | This study | **OR786499** | **OR786508** | **OR786603** |
| *Sargassum* sp. CI1 | SGU16/  TFCPhyc16464 | Spain: La Barranquera, Tenerife, Canary Islands; 31-Jan-2022; D. Alvarez-Canali | This study | **OR786500** | **OR786509** | **OR786604** |
| *Sargassum* sp. CI2 | SGU23/  TFCPhyc16471 | Spain: Cuevas Coloradas, Montaña Clara, Lanzarote, Canary Islands; 22-Sep-2020; D. Alvarez-Canali | This study | **-** | **-** | **OR786606** |
| *Sargassum stenophyllum* | SGU11/  TFCPhyc16459 | Spain: Punta Brava, Tenerife, Canary Islands; 03-Feb-2022; D. Alvarez-Canali | This study | **-** | **-** | **OR786600** |
| *Sargassum stenophyllum* | SGU12/  TFCPhyc16460 | Spain: La Barranquera, Tenerife, Canary Islands; 31-Jan-2022; D. Alvarez-Canali | This study | **-** | **-** | **OR786601** |
| *Sargassum confusum* | n.d. | South Korea: Chujado Is., Jeju; n.d.; n.d. | Lee et al., 2022 | NC_066050 | NC_066050 | NC_066050 |
| *Sargassum feldmannii* | 2017030072 | China: Sanya, Hainan; 2-Mar-2017; T. Liu | Li et al., 2021 | MW784167 | MW784167 | MW784167 |
| *Sargassum fluitans* III | C241-023-NT_1 | Atlantic Ocean; 26-May-2012; n.d. | Amaral-Zettler et al., 2016 | KY206015 | KY206020 | KY206033 |
| *Sargassum fulvellum* | n.d. | South Korea: Jeopdo Is., Jeollanamdo; n.d.; n.d. | Lee et al., 2022 | NC_066457 | NC_066457 | NC_066457 |
| **Table S4** (continued) |  |  |  |  |  |  |
| *Sargassum fusiforme* | 2015040102 | China: Wenzhou, Zhejiang Province; n.d.; n.d. | Liu et al., 2020 | NC_048511 | NC_048511 | NC_048511 |
| *Sargassum graminifolium* | 2016030038 | China: Shenzhen, Guangdong; 9-Mar-2016; T. Liu | Li et al., 2021 | MW784163 | MW784163 | MW784163 |
| *Sargassum hemiphyllum* var. *chinense* | n.d. | n.d. | Unpublished | MT873582 | MT873582 | MT873582 |
| *Sargassum henslowianum* | 2017050197 | China: Pitian, Fujian; 29-May-2017; T. Liu | Li et al., 2021 | MW784169 | MW784169 | MW784169 |
| *Sargassum horneri* | n.d. | China: Xiaohuyu, Nanji Is., Wenzhou, Zejiang Province; Apr-2007; n.d. | Liu & Pang 2016 | NC_029856 | NC_029856 | NC_029856 |
| *Sargassum kjellmanianum* | n.d. | n.d. | Unpublished | OK323194 | OK323194 | OK323194 |
| *Sargassum macrocarpum* | n.d. | South Korea: Geumodo Is., Jeollanamdo; n.d.; n.d. | Lee et al., 2022 | NC_066458 | NC_066458 | NC_066458 |
| *Sargassum mcclurei* | 2017030085 | China: Changjiang, Hainan; 3-Mar-2017; T. Liu | Li et al., 2021 | MW784168 | MW784168 | MW784168 |
| *Sargassum muticum* | 2017020007 | China: Heishijiao, Dalian; 25-Feb-2017; T Liu | Li et al., 2021 | MW784166 | MW784166 | MW784166 |
| *Sargassum natans* I | C241-025-NT_1 | Atlantic Ocean: 31.65 -64.261667; 27-May-2012; n.d. | Amaral-Zettler et al., 2016 | KY206571 | KY206585 | KY206615 |
| *Sargassum natans* VIII | C256-039-NT_32 | Atlantic Ocean: 14.9502778 -49.468611; 4-Dec-2014; n.d. | Amaral-Zettler et al., 2016 | KY206138 | KY206138 | KY206179 |
| *Sargassum phyllocystum* | 2016050141 | China: Qionghai, Hainan; 22-May-2016; T. Liu | Li et al., 2021 | MW784165 | MW784165 | MW784165 |
| *Sargassum plagiophyllum* | M03-2 | Malaysia: Penang; 1-Oct-2019; S. Draisma | Zhang et al., 2022 | NC_064732 | NC_064732 | NC_064732 |
| *Sargassum polycystum* | G6-10 | Thailand: Trat; 7-Jan-2019; S. Draisma | Zhang et al., 2022 | NC_064730 | NC_064730 | NC_064730 |
| *Sargassum serratifolium* | n.d. | South Korea: Seongsan, Jeju; n.d.; n.d. | Lee et al., 2022 | NC_066459 | NC_066459 | NC_066459 |
| *Sargassum siliquastrum* | n.d. | South Korea: Sikdo Is., Jeju; n.d.; n.d. | Lee et al., 2022 | NC_064337 | NC_064337 | NC_064337 |
| *Sargassum thunbergii* | n.d. | South Korea: Sangjokam, Doegmyeong-ri, Jeju; n.d.; n.d. | Yang et al., 2016 | NC_029134 | NC_029134 | NC_029134 |
| *Sargassum vachellianum* | n.d. | China: Gouqi Island, Zhejiang; Apr-2014; n.d. | Bi et al., 2017 | KT188823 | KT188823 | KT188823 |
|  |  |  |  |  |  |  |

**References**

Amaral-Zettler, L. A., Dragone, N. B., Schell, J., Slikas, B., Murphy, L. G., Morrall, C. E., & Zettler, E. R. (2017). Comparative mitochondrial and chloroplast genomics of a genetically distinct form of *Sargassum* contributing to recent “Golden Tides” in the Western Atlantic. *Ecology and Evolution*, *7*(2), 516–525. <https://doi.org/10.1002/ece3.2630>

Bi, Y., Li, J., & Zhou, Z. (2017). Complete sequence of chloroplast genome from *Sargassum vachellianum* (Sargassaceae, Phaeophyceae): Genome structure and comparative analysis. *Aquaculture and Fisheries*, *2*(4), 157–164. <https://doi.org/10.1016/j.aaf.2017.06.006>

Lee, Y. J., Kim, Y. D., Uh, Y. R., Kim, Y. M., Seo, T.-H., Choi, S.-J., & Jang, C. S. (2022). Complete organellar genomes of six *Sargassum* species and development of species-specific markers. *Scientific Reports*, *12*(1), Article 1. <https://doi.org/10.1038/s41598-022-25443-4>

Li, R., Jia, X., Zhang, J., Jia, S., Liu, T., Qu, J., & Wang, X. (2021). The complete plastid genomes of seven Sargassaceae species and their phylogenetic analysis. *Frontiers in Plant Science*, *12*, 747036. <https://doi.org/10.3389/fpls.2021.747036>

Liu, F., & Pang, S. (2016). Chloroplast genome of *Sargassum horneri* (Sargassaceae, Phaeophyceae): Comparative chloroplast genomics of brown algae. *Journal of Applied Phycology*, *28*(2), 1419–1426. <https://doi.org/10.1007/s10811-015-0609-2>

Liu, T., Cui, Y., Jia, X., Chen, B., Ma, Z., Zou, H., Wang, S., & Wu, M. (2020). The complete chloroplast genome of *Sargassum fusiforme*. *Mitochondrial DNA Part B*, *5*(1), 576–577. <https://doi.org/10.1080/23802359.2019.1710296>

Yang, J. H., Graf, L., Cho, C. H., Jeon, B. H., Kim, J. H., & Yoon, H. S. (2016). Complete plastid genome of an ecologically important brown alga *Sargassum thunbergii* (Fucales, Phaeophyceae). *Marine Genomics*, *28*, 17–20. <https://doi.org/10.1016/j.margen.2016.03.003>

Zhang, S., Liang, Y., Zhang, J., Draisma, S. G. A., & Duan, D. (2022). Organellar genome comparisons of *Sargassum polycystum* and *S. plagiophyllum* (Fucales, Phaeophyceae) with other *Sargassum* species. *BMC Genomics*, *23*, 629. <https://doi.org/10.1186/s12864-022-08862-5>
